# Influence of Backbone Curvature on the Organic Electrochemical Transistor Performance of Glycolated Donor–Acceptor Conjugated Polymers

**DOI:** 10.1002/anie.202106084

**Published:** 2021-08-06

**Authors:** Bowen Ding, Gunwoo Kim, Youngseok Kim, Flurin D. Eisner, Edgar Gutiérrez‐Fernández, Jaime Martín, Myung‐Han Yoon, Martin Heeney

**Affiliations:** ^1^ Department of Chemistry and Centre for Processable Electronics Imperial College London Molecular Sciences Research Hub (White City Campus) 80 Wood Lane Shepherd's Bush London W12 0BZ UK; ^2^ School of Materials Science and Engineering Gwangju Institute of Science and Technology 123 Cheomdangwagi-ro, Buk-gu Gwangju 61005 Republic of Korea; ^3^ Department of Physics and Centre for Processable Electronics Imperial College London South Kensington Campus London SW7 2AZ UK; ^4^ POLYMAT and Polymer Science and Technology Department Faculty of Chemistry University of the Basque Country UPV/EHU Manuel de Lardizabal 3 Donostia—San Sebastián Spain; ^5^ Grupo de Polímeros Departamento de Física e Ciencias da Terra Universidade da Coruña Centro de Investigacións Tecnolóxicas (CIT) Esteiro 15471 Ferrol Spain

**Keywords:** bioelectronics, conjugated backbones, organic electrochemical transistor, polymers, semiconductors

## Abstract

Two new glycolated semiconducting polymers **PgBT(F)2gT** and **PgBT(F)2gTT** of differing backbone curvatures were designed and synthesised for application as p‐type accumulation mode organic electrochemical transistor (OECT) materials. Both polymers demonstrated stable and reversible oxidation, accessible within the aqueous electrochemical window, to generate polaronic charge carriers. OECTs fabricated from **PgBT(F)2gT** featuring a curved backbone geometry attained a higher volumetric capacitance of 170 F cm^−3^. However, **PgBT(F)2gTT** with a linear backbone displayed overall superior OECT performance with a normalised peak transconductance of 3.00×10^4^ mS cm^−1^, owing to its enhanced order, expediting the charge mobility to 0.931 cm^2^ V^−1^ s^−1^.

## Graphical abstract

The targeted design of backbone geometries is shown to influence the p‐type accumulation mode organic electrochemical transistor (OECT) performance of two glycolated donor–acceptor conjugated polymers, **PgBT(F)2gT** and **PgBT(F)2gTT**.

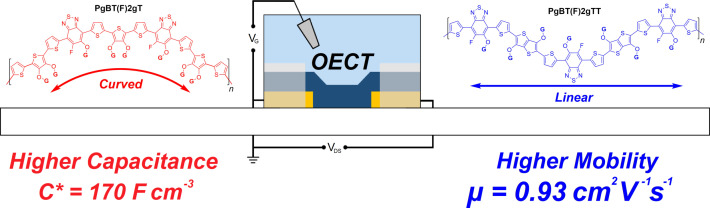

Current device research within the emergent field of organic bioelectronics is centred around the organic electrochemical transistor (OECT),^[1]^ which is recognised as a functional amplifier for biosensing^[2]^ as well as a materials testbed device from which we can springboard to other bioelectronic functionalities.[^3^, ^4^, ^5^] Unlike organic field‐effect transistors (OFETs), the modulation of charge carrying polarons/bipolarons in an OECT active material is achieved throughout the bulk of the film by gate potential induced electrochemical oxidation or reduction, giving rise to its superior volumetric capacitance.^[6]^ The electrochemical redox switching of OECTs necessitates volumetric and stoichiometric active material counterion accessibility, raising unique challenges associated with the design of OECT active materials.^[7]^

Earlier OECT conjugated polymers integrated ionic components either onto the sidechains (e.g. poly(6‐(thiophene‐3‐yl)hexane‐1‐sulfonate); PTHS)^[8]^ or within separate but intimately mixed domains (e.g. poly(3,4‐ethylenedioxythiophene):poly(styrenesulfonate); PEDOT:PSS),[^9^, ^10^] to engender mixed ionic and electronic conductivity. Aqueous solubility was a drawback of these ionic OECT materials, requiring performance diminishing cross‐linkers to be implemented into the active layer.[^11^, ^12^] Thus, OECT active material designs pivoted towards the incorporation of oligomeric glycol sidechains, as these facilitate ionic diffusion without conferring aqueous solubility.[^2^, ^13^, ^14^] Several all donor thiophene‐centric glycolated conjugated polymers have been reported for p‐type OECT applications.^[15]^

Recently, developments in OECT polymer designs have progressed towards donor–acceptor (D–A) conjugated backbones.[^16^, ^17^, ^18^, ^19^] Refined energy level tuning is an important advantage of applying D–A backbones, which can be exploited to improve electrochemical/OECT stability by avoidance of undesirable redox side‐reactions,[^20^, ^21^] as well as to ensure OECT operation in favourable accumulation mode, where channel conductivity is negligible at resting gate potential, and grows with increased gate bias (c.f. depletion mode with vice versa operational characteristics).^[22]^ However, these advantages have come at the cost of lower OECT transconductances than those observed for (less stable) all‐donor polymers. The transconductance is a key figure of merit relating to the product of the active material volumetric capacitance and charge mobility,[^13^, ^15^, ^17^] highlighting the delicate balance that must be achieved, between charge and ionic mobility, in the design of OECT active materials. Further material improvements therefore rely on better understanding the interplay between ion and charge conduction in OECT active materials. Here, for the first time, we investigate the influence of backbone curvature on the OECT performance of two p‐type D–A glycolated polymers **PgBT(F)2gT** and **PgBT(F)2gTT**, that perform contrastingly as excellent active materials.

The design of **PgBT(F)2gT** and **PgBT(F)2gTT** as p‐type accumulation mode OECT active materials combines several design facets (Figure 1). Each repeating unit of both polymers feature three triethyleneglycol monomethylether sidechains, to strike a workable balance between ion and charge transport, as well as solution processability. Attachment of the glycol sidechains via aryl‐ether linkages to either a thiophene or thieno[3,2‐*b*]thiophene affords building blocks with an angular or linear template, resulting in curved or linear conjugated backbones, respectively. The mesomeric electron donating effect of the aryl ethers is paired against the presence of an electron deficient, fluorinated, glycolated benzothiadiazole (BT) acceptor, tuning the electrochemical oxidation potentials of both polymers to within the aqueous electrochemical window, as required for p‐type OECT operation.^[23]^ The BT acceptor also serves to bridge a procession of non‐covalent S⋅⋅⋅O and S⋅⋅⋅F planarising interactions along the backbones of **PgBT(F)2gT** and **PgBT(F)2gTT**, to promote backbone planarity, stronger orbital hybridisation/delocalisation and enhanced charge transport.^[24]^

**Figure 1:**
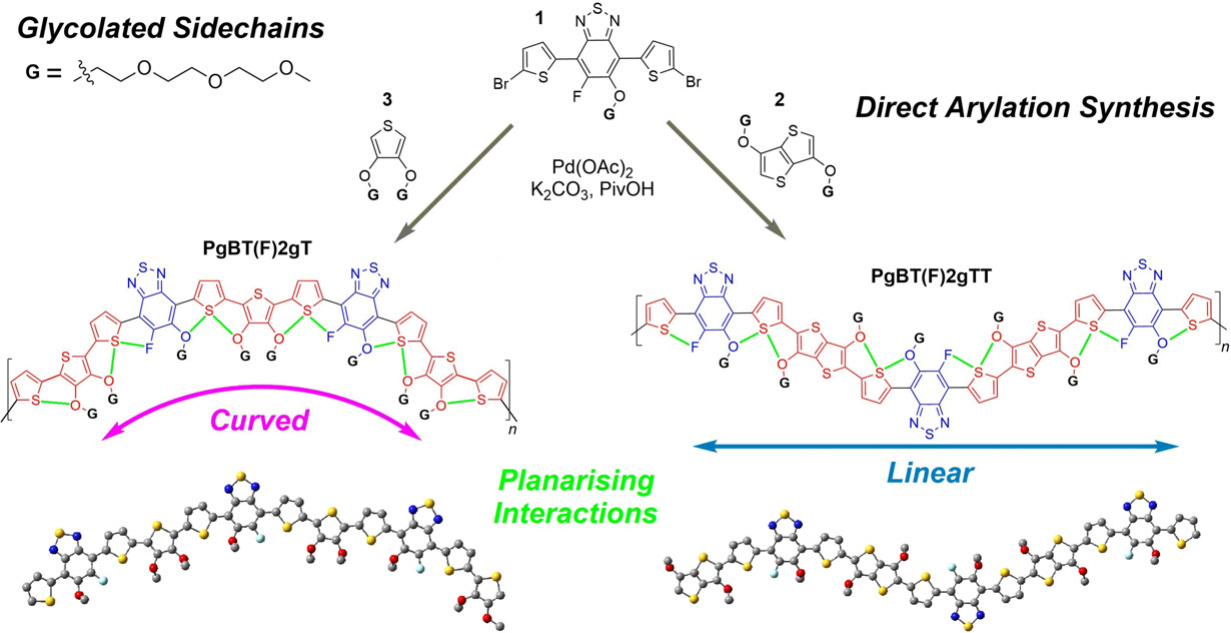
Synthesis and design of **PgBT(F)2gT** and **PgBT(F)2gTT**, with DFT optimised representations (bottom).

To investigate the role of non‐covalent interactions along backbones of **PgBT(F)2gT** and **PgBT(F)2gTT**, DFT calculations of trimeric models at the B3LYP/6‐31G(d,p) level were performed (Figures 1, S1–S8).^[25]^ Potential energy scans were also performed on 3,4‐dimethoxythiophene‐thiophene as well as 3,6‐dimethoxythienothiophene‐thiophene components, revealing an optimal *anti* arrangement, with a small dihedral angle of 6° between the two heterocycles in both systems and an approximate 5 kJ mol^−1^ lower energy than the *syn* conformer (Figures S9,S10). The preference for *anti* over *syn* is in agreement with crystal structures of related methoxy substituted oligothiophenes,[^26^, ^27^] as well as earlier studies examining the role of non‐covalent S⋅⋅⋅O interactions in bithiophenes.[^28^, ^29^] The planarity and preferred conformation (thiophenes *trans* with respect to thiadiazole) of the dithienoBT unit common to both **PgBT(F)2gT** and **PgBT(F)2gTT** has been established previously.[^30^, ^31^] Starting from preferred conformations, energy minimisations of trimeric species revealed both backbones are almost fully coplanar. The positioning of the planarising interactions and sidechains on one side of the conjugated backbone of **PgBT(F)2gT** forces it to adopt a curved backbone geometry, whereas even distribution of sidechains and planarising interactions about both sides of the **PgBT(F)2gTT** backbone template its more linear geometry. This differing curvature is maintained regardless of the regiochemistry of the asymmetric BT within both backbones. In OFET applications, backbone curvature of conjugated polymers has been found to significantly influence their charge transport properties.[^32^, ^33^] We observed good orbital mixing in **PgBT(F)2gT** and **PgBT(F)2gTT** as evidenced by their highly delocalised highest occupied molecular orbitals (HOMOs).

Regiorandom **PgBT(F)2gT** and **PgBT(F)2gTT** were synthesised by direct arylation polymerisation of dibrominated monomer **1** with glycolated thienothiophene **2** and thiophene **3** (Figure 1), bypassing the necessity for toxic organometallic reagents.^[34]^ Direct arylation polymerisations were performed at 80 °C to suppress crosslinking.^[35]^ Both polymers were isolated in high yield (>80 %) following precipitation and sequential solvent washing to remove impurities and low weight material. As with many reported glycolated conjugated polymers, the analysis of molecular weight was complicated by the tendency of both polymers to aggregate in solution.[^36^, ^37^] Examining different concentrations of **PgBT(F)2gT** by GPC revealed an estimated *M_n_* =10 kDa and *Đ*=1.7 against polystyrene standards, whereas the main peak of **PgBT(F)2gTT** exhibited unrealistically high values irrespective of concentration with a small peak also apparent at *M_n_* =3.8 kDa and *Đ*=1.2 (Figures S11–S13). The structures of both polymers were confirmed by ^1^H and ^19^F NMR, as well as MALDI‐ToF analysis (Figures S23–S28).

Solution state UV/Vis spectra in CHCl_3_ (Figures S29–32) of **PgBT(F)2gT** and **PgBT(F)2gTT** revealed evidence of aggregation. Both polymers exhibited a main absorption around 600 nm with a longer wavelength shoulder which dissipated at increased temperatures. UV/Vis of **PgBT(F)2gT** and **PgBT(F)2gTT** thin films (Figures S33,S34) revealed red‐shifted onsets of absorption and broadened S0‐S1 transitions compared to the solution state, which can be explained by solid state planarisation and packing. The optical band gaps of **PgBT(F)2gT** and **PgBT(F)2gTT** were calculated to be 1.8 eV from the intersection of their solution state UV/Vis and fluorescence traces (Figures S35,S36).

According to both solid state cyclic voltammetry (CV) and square‐wave voltammetry (SQW) electrochemical data in 0.1 M KCl/H_2_O (Figure 2), the onset of **PgBT(F)2gT** thin film oxidation occurs at 0.4 V vs. Ag/AgCl (HOMO=−4.8 eV^[38]^), which lies well within the aqueous electrochemical window but sufficiently anodic to avoid material ambient auto‐oxidation. Cycling CV experiments suggested excellent electrochemical stability and reversibility of **PgBT(F)2gT** oxidation (Figure 2 e). Scan rate dependence CV data were collected to demonstrate the volumetric penetration of counterions into **PgBT(F)2gT** thin films upon bulk electrochemical oxidation, which revealed the diffusion limited nature of thin film oxidation in accordance with the Randles–Sevcik equation.^[39]^ Similarly, the onset of **PgBT(F)2gTT** thin film oxidation was observed at a felicitously accessible potential of 0.3 V during CV and SQW experiments (HOMO=−4.7 eV^[38]^), with volumetric counterion diffusion upon oxidation, as well as its electrochemical stability and reversibility, confirmed by scan rate dependence and cycling CV studies, respectively. Thin film electrochemistry data for **PgBT(F)2gT** and **PgBT(F)2gTT** were also recorded in 0.1 M [*n*‐Bu_4_N]PF_6_/MeCN to compliment aqueous electrolyte results (Figures S37–S44), revealing similar behaviours.

**Figure 2:**
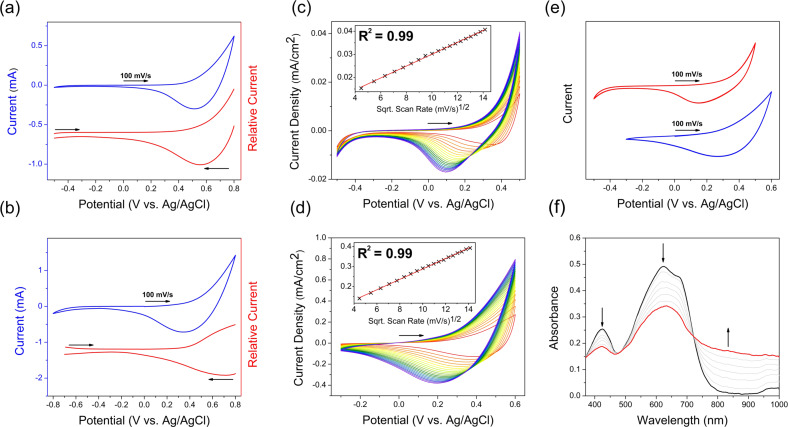
Thin film electrochemistry in 0.1 M KCl/H_2_O of a) **PgBT(F)2gT** and b) **PgBT(F)2gTT** showing CV (blue) and SQW (red); 20–200 mV s^−1^ scan rate dependence CV of c) **PgBT(F)2gT** and d) **PgBT(F)2gTT** with insets showing peak currents at 0.50 and 0.45 V vs. Ag/AgCl (respectively) against the square roots of scan rates (linear regression in red); e) 36 scan cycling CV for **PgBT(F)2gT** (red) and **PgBT(F)2gTT** (blue); and f) **PgBT(F)2gT** UV/Vis spectroelectrochemistry, at applied potentials of 0 V (black), 0.8 V (red) and intermittent values (grey). Arrows indicate scan directions and spectral progression.

Solid state thin film UV/Vis spectroelectrochemistry (SEC) in 0.1 M KCl/H_2_O was applied to identify the electrochemically oxidised state of **PgBT(F)2gT** (Figure 2 f). Upon applying an oxidative potential of 0.4 V vs. Ag/AgCl, which was incremented up to 0.8 V, gradual quenching of the **PgBT(F)2gT** ground state transitions was observed, concurrent with the appearance and intensification of a broad polaron band at 850 nm. Subsequently returning the applied potential to 0 V resulted in a restoration of the **PgBT(F)2gT** ground state UV/Vis spectrum, evidencing electrochemical reversibility (Figure S46). Similarly, in the reversible UV/Vis SEC of **PgBT(F)2gTT** thin films (Figure S47), a broad polaron band (and a bleaching of ground state transitions) transpired at an oxidative potential of 0.3 V, peaking in intensity at an apogean anodic potential of 0.8 V. Thus, UV/Vis SEC confirms reversible generation of mobile charge carrying hole polarons on **PgBT(F)2gT** and **PgBT(F)2gTT** by electrochemical oxidation.

The *p*‐type accumulation mode OECT performance of devices fabricated using **PgBT(F)2gT** and **PgBT(F)2gTT** were interpreted using the transconductance expression [Eq. 1, Table 1, and Figures 3, S48–S51; *I_D_* represents source‐drain current and *V_G_* represents gate voltage].^[6]^

(1) $$g_{m}=\frac{\partial I_{D}}{\partial V_{G}}=\mu C{}^{*}\frac{Wd}{L}\left(V_{Th}-V_{G}\right)$$

**Figure 3:**
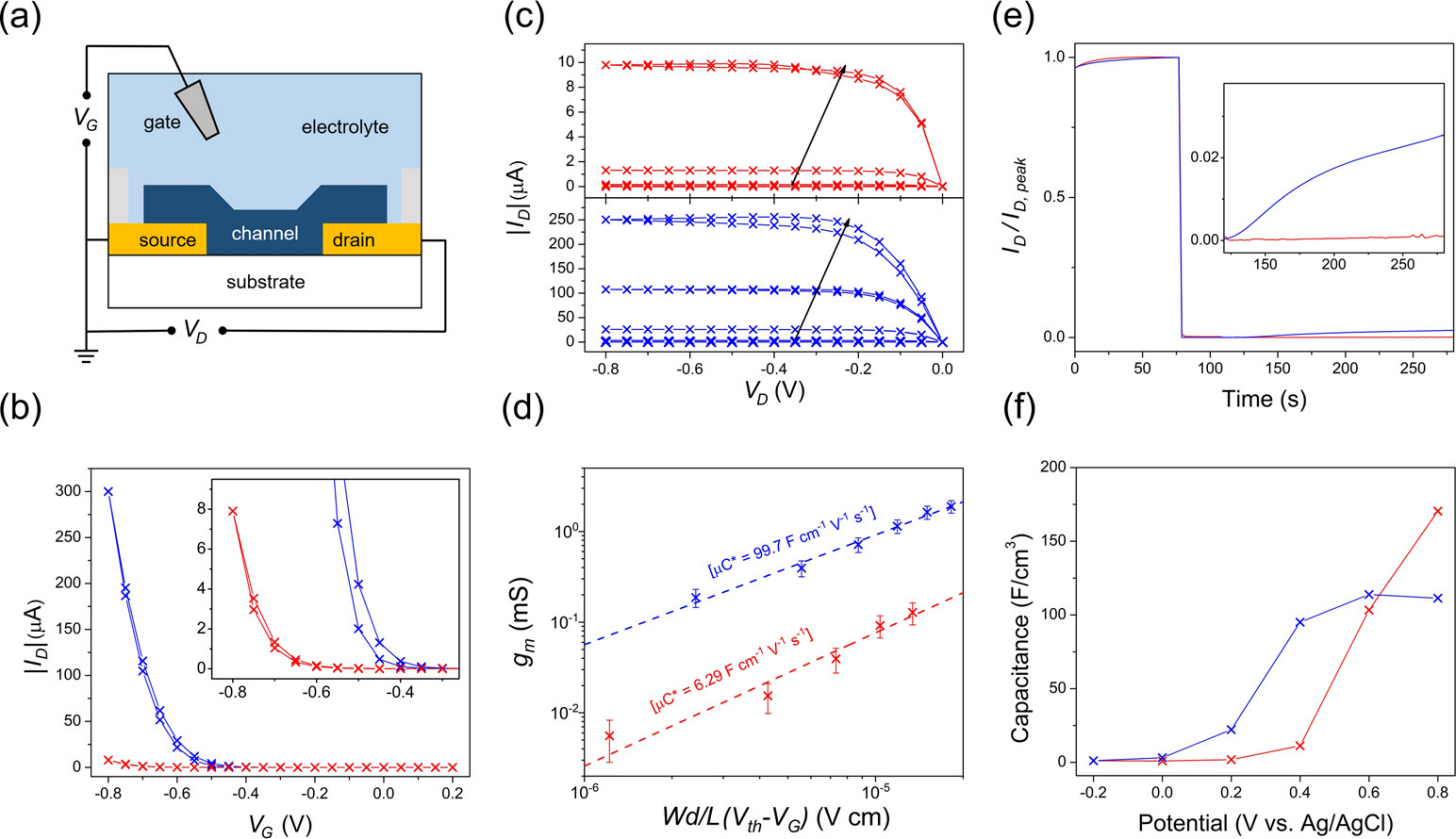
OECT performance of **PgBT(F)2gT** (red) and **PgBT(F)2gTT** (blue), showing (a) device architecture; b) transfer curves at *V_D_* =−0.60 V, with inset magnifying onto **PgBT(F)2gT** data; c) OECT output curves at stepped *V_G_* from 0 to −0.8 V in 0.1 V intervals (arrows indicate data at increased *V_G_*); d) plot of transconductance against channel dimensions and operational parameters, to extract μC*; e) device *I_D_* stabilities in air upon switching gate potential OFF; and f) volumetric capacitances measured by EIS.

**Table 1:** OECT performance metrics.

Material	*W* [μm]^[a]^	*L* [μm]^[a]^	*d* [nm]^[a]^	*V_Th_* [V]^[b]^	*I_ON_/I_OFF_* ^[c]^	*g_m_* [mS]^[d]^	C* [F cm^−3^, EIS]^[e]^	*μ* [cm^2^ V^−1^ s^−1^]^[f]^
**PgBT(F)2gT**	100	20	122	−0.58 ±0.01	10^3^	0.133 ±0.062	170	0.060 ±0.029
**PgBT(F)2gTT**	100	20	126	−0.51 ±0.01	10^5^	1.89 ±0.302	111	0.931 ±0.149

[a] *W*/*d*/*L* are channel dimensions. [b] Threshold voltage. [c] ON/OFF ratio. [d] Transconductance. [e] Volumetric capacitance; measured by EIS (electrochemical impedance spectroscopy, using a conventional 3 electrode system). [f] Charge mobility.

OECTs fabricated using **PgBT(F)2gT** exhibited a higher volumetric capacitance of 170 F cm^−3^ at *V_G_* =−0.8 V, where channel mobility was calculated at 0.060 cm^2^ V^−1^ s^−1^, corresponding to a (channel dimension) normalised peak transconductance of 2.18×10^3^ mS cm^−1^. A higher normalised peak transconductance of 3.00×10^4^ mS cm^−1^ at *V_G_* =−0.8 V was recorded for OECTs employing **PgBT(F)2gTT**, which was ascribed to its superior channel mobility of 0.931 cm^2^ V^−1^ s^−1^, despite an inferior volumetric capacitance of 111 F cm^−3^. Note that both materials show volume‐dependent capacitance increase (Figures S52,S53), within a thickness range up to 200 nm (typical for devices). The OECT ON/OFF ratio of **PgBT(F)2gTT** was much higher at 10^5^ than **PgBT(F)2gT** at 10^3^, and is comparable with state‐of‐the‐art devices.^[22]^ Furthermore, there is no significant difference in operational cut‐off frequency between **PgBT(F)2gT** and **PgBT(F)2gTT** (Figure S54).

During cyclic switching of OECT devices, both materials show good operational stability in *V_G_* =−0.6 V up to 100 cycles, but exhibit slight degradation under an excessive gating voltage of −0.8 V (Figure S55). The reduced stability during cycling up to *V_G_* =−0.8 V maybe attributed to the negative effects of repeated volume expansions on the hopping dominated interchain transport occurring in both polymers.^[40]^ In terms of the redox resilience of both polymers under ambient conditions, OFF‐current rising of **PgBT(F)2gT** was lower than **PgBT(F)2gTT** due to its lower HOMO level (Figure 3 e).^[20]^

The structural features of **PgBT(F)2gT** and **PgBT(F)2gTT** thin films were investigated to understand the influence of materials design on their differing OECT performances. Grazing‐incidence wide‐angle X‐ray scattering (GIWAXS, Figure 4) revealed varying structural order in both materials. **PgBT(F)2gTT** thin films exhibited an approximate 1:1 mixture of face‐on and edge‐on crystallite orientations, as deduced from “lamellar” peaks (100) (at *q*=3.49 nm^−1^, *d‐spacing*=1.8 nm) and a π–π stacking peak (at *q*=17.3 nm^−1^, *d‐spacing*=0.36 nm) showing up along both *q_r_* and *q_z_* directions. On the other hand, **PgBT(F)2gT** thin films exhibited preferred face‐on orientation and an increased *d‐spacing* of 2.16 nm, with the (100) and π–π stacking peaks oriented along the *q_r_* and *q_z_* directions, respectively. These structural differences can be correlated to backbone geometries; linear **PgBT(F)2gTT** has greater macromolecular symmetry, facilitating bimodal crystallinity, whereas curved **PgBT(F)2gT** is of lower backbone symmetry, which limits the directions of stackable crystalline propagation.^[41]^ Atomic‐force microscopy (AFM, Figures S56–S59) images supported these findings, with **PgBT(F)2gTT** exhibiting a microfibrillar structure comprising of fibrils 10–20 nm wide and 30–50 nm long. AFM images of **PgBT(F)2gT** appeared relatively featureless, in agreement with its less pronounced crystallinity.

**Figure 4:**
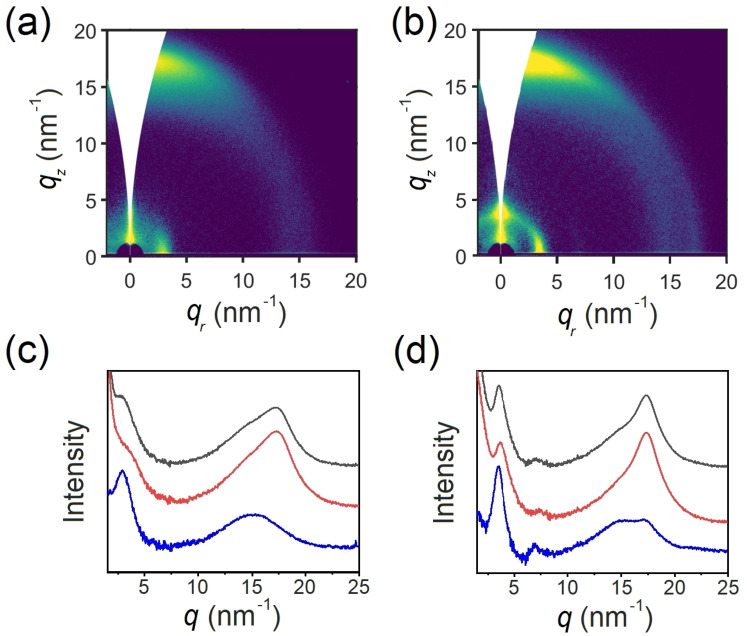
GIWAXS of a) **PgBT(F)2gT** and b) **PgBT(F)2gTT**, with isotropic integrations of scattering (black), and integrations centred about the *q_z_* (red, out of plane) and *q_r_* (blue, in plane) directions shown for c) **PgBT(F)2gT** and d) **PgBT(F)2gTT**.

The higher OECT channel mobility of **PgBT(F)2gTT** is thus explained by its bimodal crystallinity, enabling effective source‐drain charge transport. The limited geometric capacity for **PgBT(F)2gT** to stack only in the face‐on orientation hinders charge transport in the source‐drain direction of the OECT channel. However, ease of counterion diffusion along pervasive amorphous channels in **PgBT(F)2gT** enables its larger volumetric capacitance.^[42]^

Fundamental characterisation and device performance of **PgBT(F)2gT** and **PgBT(F)2gTT** conclusively demonstrate the success of their design as state‐of‐the‐art OECT active materials. **PgBT(F)2gT** and **PgBT(F)2gTT** exhibit highly reversible and stable electrochemical oxidation enabling their application as *p*‐type accumulation mode OECT materials, as shown by their CV, SQW and SEC behaviour. OECTs constructed with **PgBT(F)2gT** performed with a superior volumetric capacitance of up to 170 F cm^−3^, ascribed to its low symmetry, curved backbone design templating comparatively amorphous (ion‐diffusive) films. In contrast, OECTs employing **PgBT(F)2gTT** attained a higher charge mobility of 0.931 cm^2^ V^−1^ s^−1^, owing to its linear backbone design facilitating bimodal crystallinity. Overall, OECTs featuring **PgBT(F)2gTT** displayed the best performance, attaining a normalised peak transconductance of 3.00×10^4^ mS cm^−1^. To fine‐tune OECT performance, future work will focus on further backbone functionalisation of both polymers by the nucleophilic aromatic subsitution of the fluorine atoms on BT.^[43]^

## Conflict of interest

The authors declare no conflict of interest.

## Supporting information

As a service to our authors and readers, this journal provides supporting information supplied by the authors. Such materials are peer reviewed and may be re‐organized for online delivery, but are not copy‐edited or typeset. Technical support issues arising from supporting information (other than missing files) should be addressed to the authors.

Supporting Information
