# Supplementary material for: Influence of Backbone Curvature on the Organic Electrochemical Transistor Performance of Glycolated Donor–Acceptor Conjugated Polymers
Source: Angew Chem Int Ed Engl. 2021 Aug 6;60(36):19679–84. doi: 10.1002/anie.202106084 (PMC8457089; doi:10.1002/anie.202106084)
Supplement: Supplementary file 1 — Supporting Information [file ANIE-60-19679-s001.pdf]

Angewandte Chemie
Eine Zeitschrift der Gesellschaft Deutscher Chemiker

Supporting Information

Influence of Backbone Curvature on the Organic Electrochemical Transistor Performance of Glycolated Donor–Acceptor Conjugated Polymers

Bowen Ding+, Gunwoo Kim+, Youngseok Kim+, Flurin D. Eisner, Edgar Gutiérrez-Fernández, Jaime Martín, Myung-Han Yoon, and Martin Heeney**

anie_202106084_sm_miscellaneous_information.pdf

Supplementary Information

EXPERIMENTAL DETAILS

Materials

All reagents and solvents were obtained commercially and used without further purification unless otherwise stated. Pellets of K_2CO_3 were ground to fine powder form and dried in an oven before use. KCl and [*n*-Bu_4N]PF_6 for electrochemistry were recrystallised twice in H_2O and EtOH respectively. All substrates (including 2 cm^2 FTO slides for electrochemistry and spectroelectrochemistry, 2.25 cm^2 glass slides for general thin film analyses, 2.25 cm^2 glass substrates for OECT fabrication and 2.25 cm^2 Si wafer pieces for GIWAXS) were cleaned thoroughly by sonication before use; first in soapy water, then thrice each in distilled water, acetone and finally propan-2-ol. Si wafer substrates for GIWAXS and glass substrates for AFM were further treated with a UV-ozone cleaner prior to spin-coating. 4,7-Bis(thien-2-yl)-5,6-difluoro-2,1,3-benzothiadiazole was synthesised according to literature procedure.[1]

General Methods

Solution state ^1H, ^{19}F and $^{13}C\{^1H\}$ NMR spectra of precursor compounds were collected on either a Bruker AVANCE 400 or 500 MHz spectrometer at a temperature of 298 K. Solution state ^1H and ^{19}F NMR spectra of polymers were collected on a Bruker AVANCE 400 MHz spectrometer at an elevated temperature of 328 K. Deuterated solvents used for NMR were obtained from Sigma-Aldrich, and their solvent residual signals were used as internal references for chemical shifts (δ). Solid state total reflectance ATR-IR spectra were obtained on an Agilent CARY 630 FTIR spectrometer. High resolution mass spectrometry data were collected using a Thermo Scientific Q-Exactive/Dionex Ultimate 3000 operated in either ES or APCI ionisation modes. Matrix-Assisted Laser Desorption/Ionisation Time-of-Flight (MALDI-ToF) mass spectrometry data were collected on a Micromass MALDI-ToF analyser. Samples were cast in a 2,2′:5′,2″-terthiophene matrix from THF solutions, with collections operated in positive mode along mass ranges (*m/z*) tuned according to sample. The M_n, M_w and Đ of polymers dissolved in $CHCl_3$ (against polystyrene standards) were determined using an Agilent 1260 Infinity GPC running at an oven temperature of 40 °C, employing the 7.5 mm diameter PLgel 5 µm Mixed-B column. Differential scanning calorimetry (DSC) traces of polymers between 40 – 300 °C were collected on a Mettler DSC822e differential scanning calorimeter, at a heating rate of 10 °C/min under a N_2 environment. UV-Vis data were collected using either a Shimadzu UV-1601 spectrometer interfaced to Shimadzu software, or an Agilent CARY 60 UV-Vis spectrometer interfaced to SCAN software.

Spectroscopic grade DCM and $CHCl_3$ were used for solution state measurements of precursor compounds and polymers respectively, with molar extinction coefficients of polymers in solution estimated using molar masses of repeating units. Solid state UV-Vis measurements were collected using thin films drop-cast onto pre-cleaned FTO slides. Drop-casting of thin films were conducted using standard solutions containing 2.5 mg of sample dissolved in 1 mL $CHCl_3$. No thermal annealing of thin films was performed, in accordance with OECT fabrication protocols. An Agilent Eclipse Fluorescence spectrophotometer was used for the collection of fluorescence data of polymers dissolved in spectroscopic grade $CHCl_3$. Thin film X-ray diffraction (XRD) data were collected with a Bruker D2 Phaser Diffractometer producing Cu-K_α (λ = 1.5418 Å) radiation, fitted with an SSD 160 detector. Polymer thin films for XRD were drop-cast (without thermal annealing) from $CHCl_3$ solutions at concentrations of 10 mg/mL, onto pre-cleaned glass substrates. Atomic force microscopy (AFM) images of polymer thin films spun-coated onto glass substrates were captured using an Agilent 5500 operated in tapping mode. Grazing-incidence wide angle X-ray scattering (GIWAXS) patterns were collected at the ALBA synchrotron Spain, on polymer thin films spun-coated onto Si wafer substrates. For details of spin-coating conditions, please refer to the section discussing OECT fabrication. Thicknesses of thin films were measured using a Dektak profilometer.

Synthesis

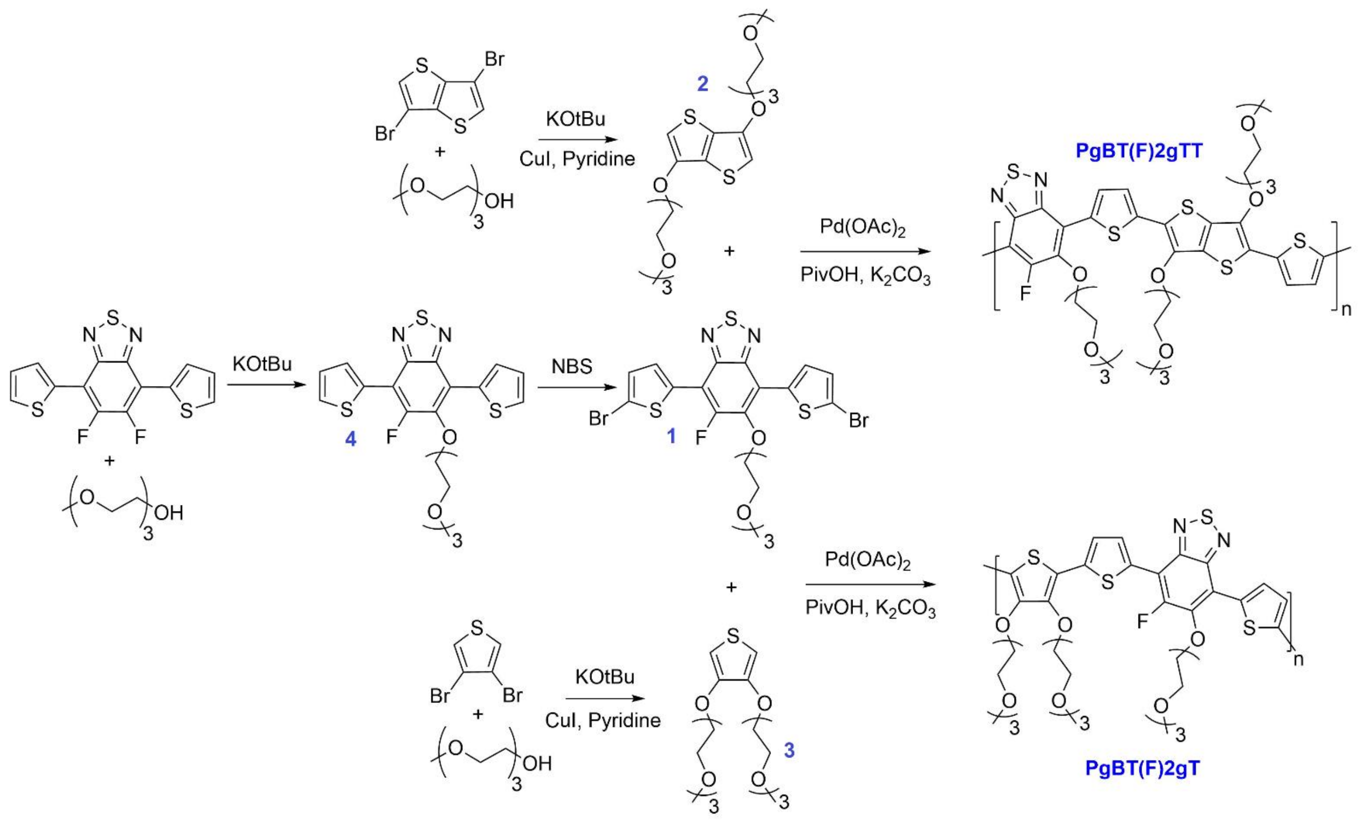

Scheme S1: Synthetic summary of polymers **PgBT(F)2gT** and **PgBT(F)2gTT**, with numberings for precursor compounds also shown in blue.

4,7-Bis(thien-2-yl)-5-triethyleneglycolmonomethylether-6-fluoro-2,1,3-benzothiadiazole (**4**)

4,7-Bis(thien-2-yl)-5,6-difluoro-2,1,3-benzothiadiazole (0.401 g, 1.19 mmol) and KOtBu (0.134 g, 1.19 mmol) were weighed into a dried 2-neck 100 mL RBF and purged under a stream of N_2 for 15 mins. Dry THF (50 mL) was added to the reaction, followed by triethyleneglycol monomethylether (187 µL, 0.191 g, 1.07 mmol). The resulting deep orange mixture was heated to reflux in the dark under N_2 for 60 h. Upon completion, the reaction was cooled to RT, diluted into DCM (200 mL) and washed with brine (4 × 200 mL). The organic fraction was collected, dried with $MgSO_4$, and the solvent removed by rotary evaporation to give a red oil as the crude product. This was purified by silica gel column chromatography eluting 3:1 hexane/EtOAc (R_f = 0.25) to give the title compound as an orange-red oil (0.262 g, 44%). ^{1}H NMR ($CDCl_3$, 400 MHz, 298 K): δ 8.52 (dd, $^3J_{H\text{-}H}$ = 4 Hz, $^4J_{H\text{-}H}$ = 1 Hz, 1H), 8.28 (d, $^3J_{H\text{-}H}$ = 4 Hz, 1H), 7.59 (dd, $^3J_{H\text{-}H}$ = 5 Hz, $^4J_{H\text{-}H}$ = 1 Hz, 1H), 7.54 (dd, $^3J_{H\text{-}H}$ = 5 Hz, $^4J_{H\text{-}H}$ = 1 Hz, 1H), 7.28 – 7.23 (m, 2H), 4.36 (t, $^3J_{H\text{-}H}$ = 5 Hz, 2H), 3.94 (t, $^3J_{H\text{-}H}$ = 5 Hz, 2H), 3.71 – 3.68 (m, 2H), 3.66 – 3.60 (m, 4H), 3.54 – 3.50 (m, 2H), 3.36 (s, 3H) ppm. ^{13}C{^{1}H} NMR ($CDCl_3$, 125 MHz, 298 K): δ 156.0, 153.9, 150.3, 149.8, 149.7, 146.6, 146.5, 133.6, 133.5, 132.3, 132.2, 131.3, 130.7, 130.6, 128.4, 128.33, 128.25, 127.4, 127.1, 117.57, 117.55, 111.7, 111.6, 73.55, 73.50, 72.06, 70.90, 70.79, 70.74, 70.45, 59.19 ppm (individual C-F splitting could not be assigned). ^{19}F NMR ($CDCl_3$, 400 MHz, 298 K): δ -120.0 (s, 1F) ppm. HRMS (APCI$^+$): calculated for $C_{21}H_{22}N_2O_4S_3F$ $[M+H]^+$, 481.0720; found, 481.0701.

4,7-Bis(5-bromo-thien-2-yl)-5-triethyleneglycolmonomethylether-6-fluoro-2,1,3-benzothiadiazole (**1**)

Compound **1** (0.146 g, 0.304 mmol) was charged into a 2-neck 25 mL RBF as an orange solution in dry THF (11 mL). This was then purged under N_2 for 15 mins, upon which *N*-bromosuccinimide (0.122 g, 0.685 mmol) was portion-wise. The reaction was left to stir in the dark at RT under N_2 for 21 h. Upon completion, the reaction mixture was diluted into DCM (100 mL) and washed with brine (4 × 200 mL). The organic fraction was collected, dried with $MgSO_4$, and the solvent removed by rotary evaporation to give a deep red oil that solidified upon cooling (0.188 g, 97%). This was used immediately for polymerisations without further purification. ^{1}H NMR ($CDCl_3$, 400 MHz, 298 K): δ 8.50 (d, $^3J_{H\text{-}H}$ = 4 Hz, 1H), 8.03 (d, $^3J_{H\text{-}H}$ = 4 Hz, 1H), 7.22 – 7.18 (m, 2H), 4.40 (t, $^3J_{H\text{-}H}$ = 5 Hz, 2H), 3.95 (t, $^3J_{H\text{-}H}$ = 5 Hz, 2H),

3.72 – 3.67 (m, 2H), 3.66 – 3.60 (m, 4H), 3.54 – 3.50 (m, 2H), 3.37 (s, 3H) ppm. ^{19}F NMR ($CDCl_3$, 400 MHz, 298 K): δ -119.8 (s, 1F) ppm. HRMS (ES$^+$): calculated for $C_{21}H_{19}N_2O_4S_3FBr_2Na$ [M+Na]$^+$, 658.8755; found, 658.8759.

3,6-Bis(triethyleneglycolmonomethylether)-thieno[3,2]thiophene (**2**)

CuI (1.28 g, 6.72 mmol) and KOtBu (5.66 g, 50.4 mmol) were added to a dried 100 mL 2-neck RBF and purged under N_2 for 20 mins, upon which toluene (55 mL), pyridine (5.5 mL) and triethyleneglycol monomethylether (8.2 mL, 8.4 g, 47 mmol) were added in that order. The resulting brown suspension was left to stir at RT under N_2 for 1 h, upon which 3,6-dibromothieno[3,2]thiophene (5.00 g, 16.8 mmol) was added to the reaction. This was heated to reflux in the dark under N_2 for 15 h. Upon cooling to RT, the supernatant black liquid was collected by decanting and concentrated to <10 mL by rotary evaporation. This black residue was then diluted into DCM (400 mL) and washed with HCl (5M, 4 × 400 mL). The organic phase was collected, dried with $MgSO_4$ and the solvent removed by rotary evaporation to give a black residue. This was purified twice by silica gel column chromatography eluting 1:1 hexane/EtOAc followed by EtOAc (R_f = 0.39 in EtOAc) to give the title compound as a light brown oil that solidified to an off-white solid upon chilling in a freezer (1.18 g, 15%). ^{1}H NMR ($CDCl_3$, 400 MHz, 298 K): δ 6.28 (s, 2H), 4.23 (t, $^3J_{H\text{-}H}$ = 5 Hz, 4H), 3.88 (t, $^3J_{H\text{-}H}$ = 5 Hz, 4H), 3.77 – 3.73 (m, 4H), 3.71 – 3.64 (m, 8H), 3.57 – 3.53 (m, 4H), 3.38 (s, 6H) ppm. ^{13}C{^{1}H} NMR ($CDCl_3$, 100 MHz, 298 K): δ 149.9, 128.8, 98.68, 72.10, 71.08, 70.83, 70.75, 70.14, 69.69, 59.20 ppm. HRMS (APCI$^+$): calculated for $C_{20}H_{33}O_8S_2$ [M+H]$^+$, 465.1611; found, 465.1595.

3,4-Bis(triethyleneglycolmonomethylether)thiophene (**3**)

CuI (0.316 g, 1.66 mmol) and KOtBu (1.39 g, 12.4 mmol) were added to a dried 50 mL 2-neck RBF and purged under N_2 for 20 mins, upon which toluene (14 mL), pyridine (1.4 mL) and triethyleneglycol monomethylether (2.0 mL, 2.0 g, 11.2 mmol) were added in that order. The resulting brown suspension was stirred under N_2 at RT for 1 h, after which 3,4-dibromothiophene was added (457 µL, 1.00 g, 4.13 mmol). The reaction was then heated to reflux in the dark under N_2 for 20 h. Upon completion and cooling to RT, the supernatant black liquid was collected by decanting and concentrated to <2 mL by rotary evaporation. This black residue was diluted into DCM (150 mL) and washed with HCl (5 M, 4 × 200 mL). The organic fraction was collected, dried with $MgSO_4$ and the solvent removed by rotary evaporation to give a black-brown residue. This was purified by silica gel column chromatography eluting 1:1 hexane/EtOAc followed by EtOAc (R_f = 0.20 in EtOAc) to give the title compound as a yellow oil (0.698 g, 41%). ^{1}H

NMR ($CDCl_3$, 400 MHz, 298 K): δ 6.23 (s, 2H), 4.14 (t, $^3J_{H-H}$ = 5 Hz, 4H), 3.85 (t, $^3J_{H-H}$ = 5 Hz, 4H), 3.74 – 3.70 (m, 4H), 3.68 – 3.63 (m, 8H), 3.58 – 3.53 (m, 4H), 3.38 (s, 6H) ppm. $^{13}C\{^1H\}$ NMR ($CDCl_3$, 100 MHz, 298 K): δ 147.3, 98.09, 72.10, 70.96, 70.82, 70.72, 70.00, 69.66, 59.19 ppm. HRMS ($APCI^+$): calculated for $C_{18}H_{33}O_8S$ $[M+H]^+$, 409.1891; found, 409.1877.

PgBT(F)2gT

Compound **2** (108 mg, 0.169 mmol) was dissolved in a solution of compound **4** (69.5 mg, 0.170 mmol) in dry DMAc (2 mL). The resulting dark orange solution was deoxygenated by N_2 bubbling for 45 mins. In a separate dried 5 mL microwave vial, K_2CO_3 (58.6 mg, 0.424 mmol), pivalic acid (5.2 mg, 0.051 mmol) and $Pd(OAc)_2$ (0.68 mg, 0.0030 mmol) were combined, sealed, and purged under N_2 for at least 25 mins. After deoxygenation, the DMAc solution of compounds **2** and **4** was transferred into the sealed microwave vial *via* syringe, and the entire reaction was heated to 80 ˚C in a covered oil bath. After 16 h of heating, the reaction formed into a dark blue gel, and was stopped by cooling to RT. This blue gel was precipitated into MeOH (100 mL) and the resulting suspension was sonicated for 30 mins. The solids were filtered into a Soxhlet thimble and washed by Soxhlet extraction with MeOH, hexane, acetone and EtOAc in that order. The desired product was then recovered by Soxhlet extraction with $CHCl_3$. This $CHCl_3$ fraction was concentrated to <5 mL by rotary evaporation and re-precipitated into MeOH (200 mL). The suspended solids were isolated by centrifuge and dried under high vacuum for several days to give the title polymer as a blue-black solid (144 mg, 96%). GPC ($CHCl_3$, 313 K): M_n = 10 KDa, M_w = 18 KDa and Đ = 1.7. ^1H NMR ($CDCl_3$, 400 MHz, 328 K): δ 8.52 (br s, 2H), 8.28 (br s, 2H), 4.44 (br, 6H), 4.25 – 3.45 (br m, 30H), 3.35 (br, 9H) ppm. ^{19}F NMR ($CDCl_3$, 400 MHz, 328 K): δ -119.7 (s) ppm.

PgBT(F)2gTT

Compound **2** (176 mg, 0.276 mmol) was dissolved in a solution of compound **3** (128 mg, 0.276 mmol) in dry DMAc (2 mL) and deoxygenated by N_2 bubbling for 45 mins. K_2CO_3 (97.2 mg, 0.703 mmol), pivalic acid (8.65 mg, 0.0847 mmol) and $Pd(OAc)_2$ (1.18 mg, 0.00526 mmol) were combined in a separate dried 5 mL microwave vial, which was then sealed and purged under N_2 for at least 25 mins. After deoxygenation, the dark orange solution of compounds **2** and **3** was transferred into the sealed microwave vial containing the solid phase. The reaction was then heated to 80 ˚C in a covered oil bath. The reaction turned dark blue in 15 mins and viscous after 22 h, upon which it was cooled to RT and precipitated into MeOH (100 mL). This suspension was sonicated for 30 mins and filtered into a Soxhlet thimble.

The solids were washed by Soxhlet extraction with MeOH, hexane, acetone and EtOAc, after which the polymer was extracted with $CHCl_3$. This dark blue $CHCl_3$ fraction was concentrated to <5 mL by rotary evaporation and re-precipitated into MeOH (200 mL). The suspended solids were isolated by centrifuge and dried under high vacuum for several days to give the title polymer as a blue-black solid (216 mg, 83%). GPC ($CHCl_3$, 313 K): M_n = 3.8 KDa, M_w = 4.5 KDa and Đ = 1.2. ^{1}H NMR ($CDCl_3$, 400 MHz, 328 K): δ 8.60 – 7.70 (br, 4H), 4.45 (br, 6H), 4.30 – 3.44 (br m, 30H), 3.35 (br, 9H) ppm. ^{19}F NMR ($CDCl_3$, 400 MHz, 328 K): δ -119.7 (s) ppm.

Electrochemistry

Solid state thin film cyclic voltammetry (CV) and square wave voltammetry (SQW) experiments were performed with a Metrohm Autolab PGSTAT101 Electrochemical Analyser interfaced to NOVA software. A custom made one compartment three electrode electrochemical cell was used for all measurements, featuring a 2 cm^2 FTO slide working electrode (WE) and a Pt mesh counter electrode. FTO substrates were connected to the WE assembly by a copper crocodile clip from RS electronics. The crocodile clip was suspended at a sufficient height to prevent contact with the electrolyte. An Ag/AgCl aqueous reference electrode was applied for measurements in 0.1 M KCl/H_2O. An Ag/Ag^+ non-aqueous reference electrode was applied for measurements in 0.1 M [*n*-Bu_4N]PF_6/MeCN, with the addition of ferrocene as an internal reference. Saturation of electrolyte with dried N_2 by bubbling for 20 mins was performed to deoxygenate before measurements were taken. Solid state thin film samples were drop-cast (without thermal annealing) onto the conductive side of pre-cleaned FTO slides from standard solutions containing 2.5 mg of polymer dissolved in 1 mL $CHCl_3$. Solid state thin film samples were subject to the "break in" process before electrochemical measurements were recorded. The process of "breaking in" was achieved by repeated CV cycling until stabilisation of current-voltage behaviour.

Electrochemical Impedance Spectroscopy (EIS)

An Electrochemical Analyser (Metrohm Autolab PGSTAT 302N) was employed for EIS, using 0.1 M NaCl/H_2O electrolyte, an Ag/AgCl reference electrode and a Pt counter electrode. Single sinusoidal signals ranging from 0.1 to 100 kHz (E_{AC} = 25 mV) was applied to the working electrode with E_{DC} varied from -0.2 to +0.8 V at 0.2 V intervals. EIS results were further analysed using NOVA software, by employing the equivalent circuit model consisting of one series resistor, one parallel resistor, and one parallel capacitor. Volumetric capacitance was calculated by normalizing the extracted parallel capacitance with the corresponding volume of thin films applied to the working electrode.

UV-Vis Spectroelectrochemistry

Solid state UV-Vis spectroelectrochemistry (SEC) data of **PgBT(F)2gT** and **PgBT(F)2gTT** thin films in deoxygenated 0.1 M KCl/H_2O electrolyte were collected using an Agilent CARY 60 UV-Vis spectrometer interfaced to SCAN software. A semi-compartmentalised three electrode spectroelectrochemical cell featuring quartz optical windows was used, employing a 2 cm^2 FTO slide WE which was clamped into position covering the spectrometer beamline, an Ag/AgCl aqueous reference electrode and a Pt mesh counter electrode. The applied potential was controlled incrementally using a Metrohm Autolab PGSTAT101 Electrochemical Analyser interfaced to NOVA software. Continuous UV-Vis absorbance scans were collected at various applied potentials. Thin films of **PgBT(F)2gT** and **PgBT(F)2gTT** for UV-Vis SEC were spun-coat onto FTO slides under the same conditions as outlined in the OECT fabrication section.

OECT Device Fabrication and Characterisation

The key aspects of the procedure for OECT fabrication are summarised in Scheme S2. First, positive photoresist (PPR) AZ GXR-601 was spun-cast onto the 2.25 cm^2 Corning Eagle glass substrate for source and drain electrode patterning. This PPR film was patterned by exposure to mask aligned UV light, followed by application of AZ 300 MIF developer. Then, Cr and Au electrode layers were deposited to 5 and 45 nm thicknesses respectively by thermal evaporation. Dynamic spin-coating at 4000 rpm of the OECT active layer onto the electrode patterned substrate was then performed, using 10 mg/mL polymer solutions in $CHCl_3$. A non-reactive fluoropolymer (CYTOP) was then spun-coat directly on top of the active layer at 2000 rpm, to protect the OECT channel from potential damage inflicted by organic solvents used in subsequent photolithography processes. To dry this protective layer, the CYTOP was annealed at 100 °C for 1 hour. The OECT active layer was patterned using the same aforementioned PPR photolithography method, as was applied for source and drain electrodes. Negative photoresist SU-8 2002 was then coated onto the device, patterned by mask alignment, and developed using PGMEA to form a passivation layer. Lastly, the protective CYTOP layer was removed by device immersion into a fluorinated solvent (HFE-7300), followed by overnight development.

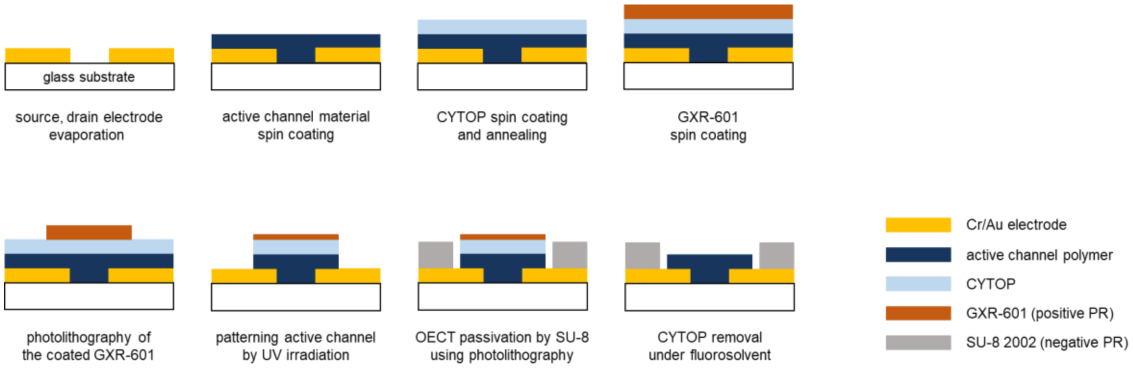

Scheme S2: Summary of OECT fabrication procedure.

OECT characterisations were performed using two Keithley 2400 source meters, for controlling source-drain and source-gate voltages, respectively. 0.1 M NaCl/H_2O electrolyte and an aqueous Ag/AgCl reference electrode from Metrohm were applied to complete each device. For transfer curve measurements, V_G was swept from +0.2 to -0.8 V at the fixed V_D of -0.6 V. For output curve measurements, V_D was swept from 0 to -0.8 V with V_G stepped from +0.2 to -0.8 V at 0.1 V intervals. V_{Th} was extracted from the *x*-intercept of a linearly fitted curve of V_G *vs.* $I_{D1/2}$, and transconductance was extracted according to the equation ($g_m = \frac{\delta I_D}{\delta V_G}$) at the saturation regime. Charge mobilities were calculated from the first differential slopes of the transfer curves. All OECT characterisations were conducted in a nitrogen glove box.

Frequency response behaviour of OECTs were characterized by sweeping the frequency (from 1 to 1000 Hz) of gate signal (sine wave of 20 mV with an offset voltage of -0.8 V) by function generator (Tektronix, AFG3021b), under the V_D of -0.6 V. The modulated drain current was acquired using a current preamplifier and data acquisition board, and transconductance at each frequency was calculated with MATLAB and ORIGIN software.

Computational Details

All computational modelling was carried out in Gaussian 09 using density functional theory (DFT) applied at the B3LYP/6-31G(d,p) level, with relevant triethyleneglycol monomethylether sidechain positions being replaced by methoxy groups for computational feasibility. Geometry optimisation and natural bond orbital population analyses were carried out on trimeric units of both reported polymers. Potential energy surface scans were carried out on locally relevant sub-repeating unit fragments. GaussView 5.0 was used for structural and orbital visualisations.

TABLES AND FIGURES

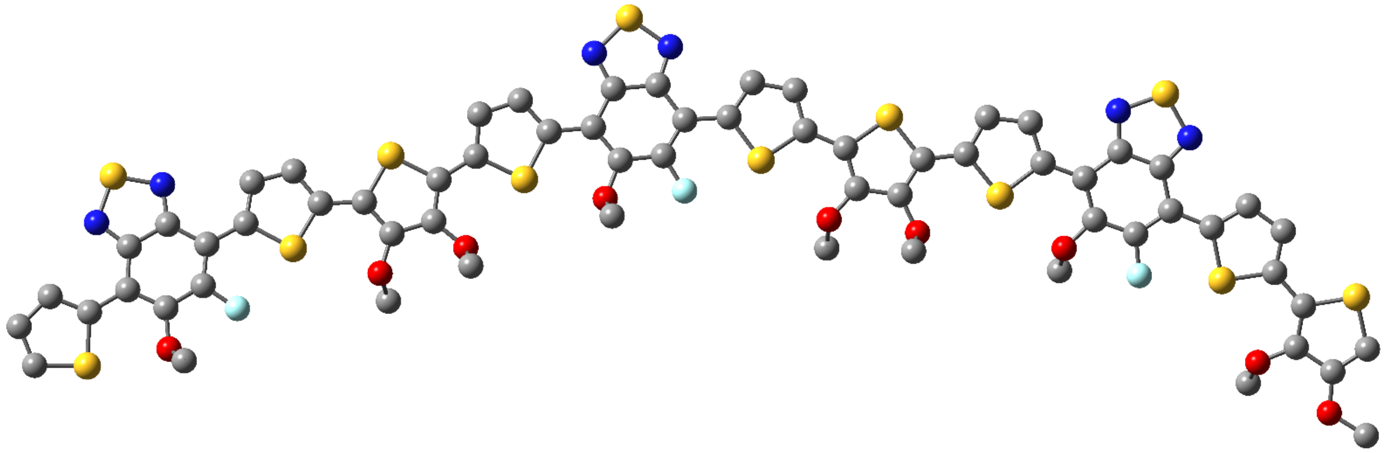

Figure S1: DFT optimisation of a trimeric model of **PgBT(F)2gT** at the B3LYP/6-31G(d,p) level showing overall backbone geometry. Hydrogen atoms omitted for clarity.

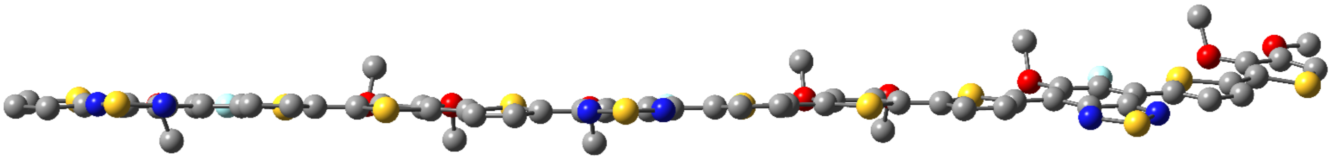

Figure S2: DFT optimisation of a trimeric model of **PgBT(F)2gT** at the B3LYP/6-31G(d,p) level showing backbone planarity. Hydrogen atoms omitted for clarity.

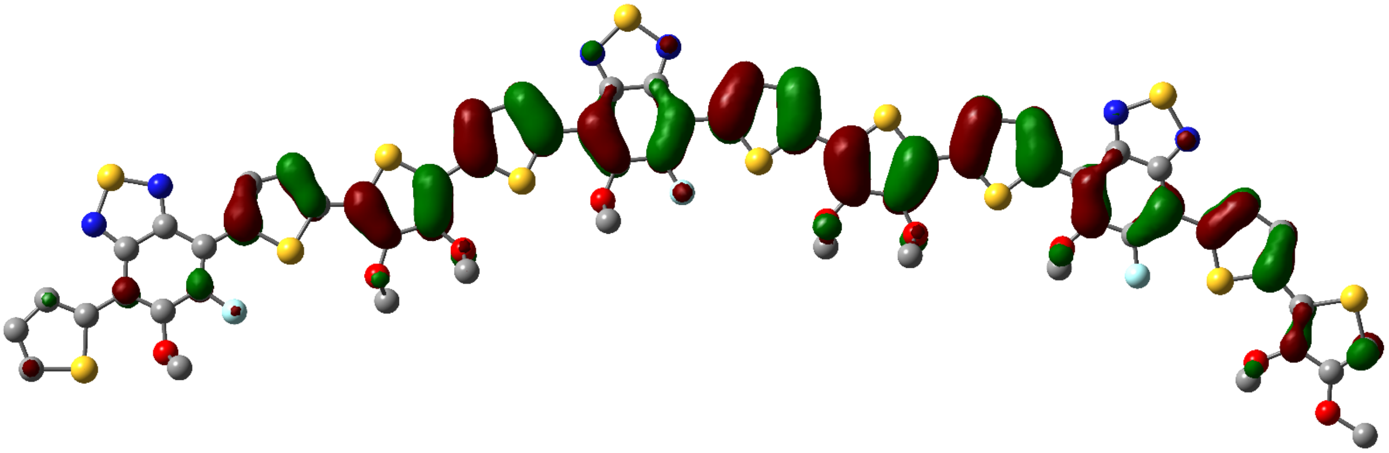

Figure S3: DFT optimisation of a trimeric model of **PgBT(F)2gT** at the B3LYP/6-31G(d,p) level showing HOMO distribution along backbone. Hydrogen atoms omitted for clarity. **PgBT(F)2gT** HOMO = -4.61 eV.

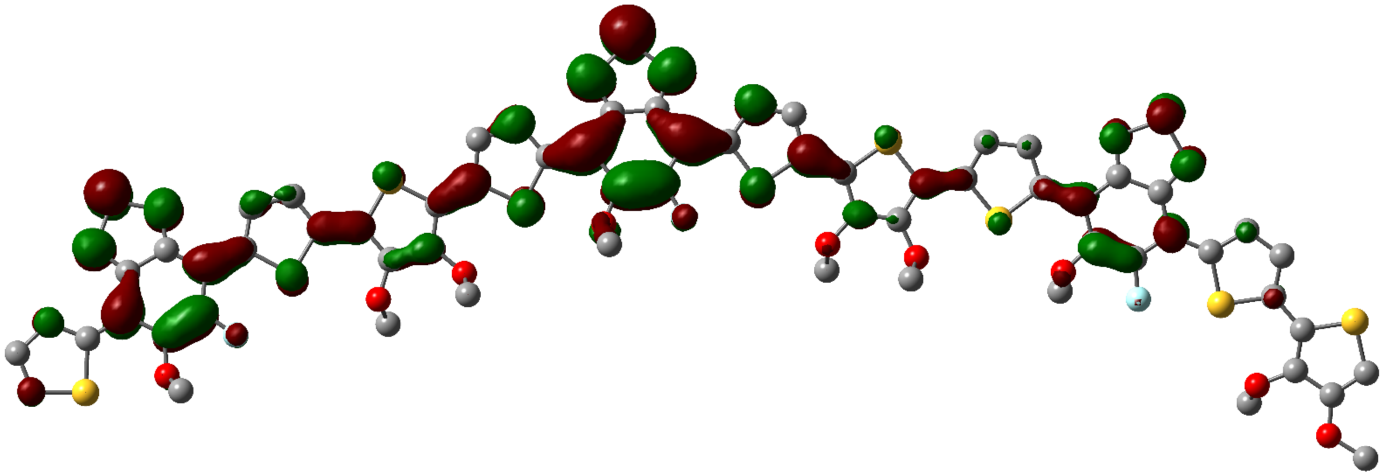

Figure S4: DFT optimisation of a trimeric model of **PgBT(F)2gT** at the B3LYP/6-31G(d,p) level showing LUMO distribution along backbone. Hydrogen atoms omitted for clarity. **PgBT(F)2gT** LUMO = -2.71 eV.

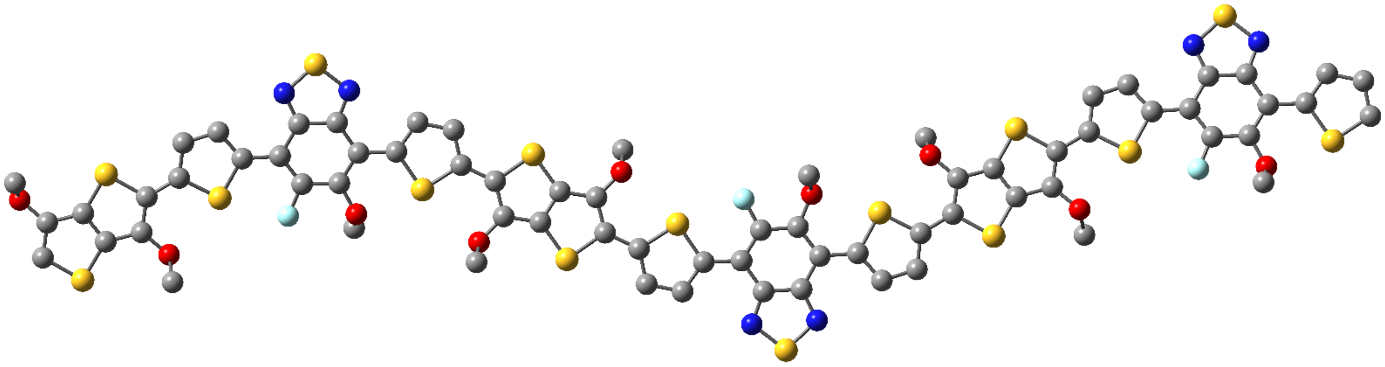

Figure S5: DFT optimisation of a trimeric model of **PgBT(F)2gTT** at the B3LYP/6-31G(d,p) level showing overall backbone geometry. Hydrogen atoms omitted for clarity.

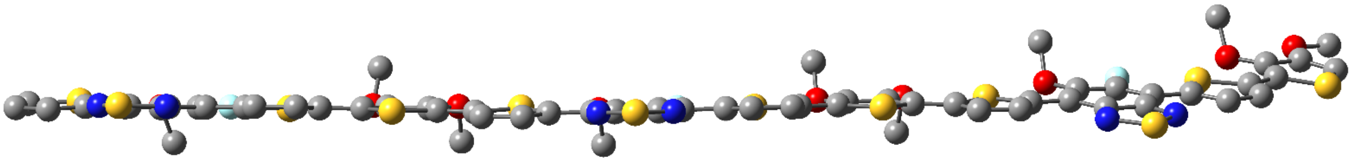

Figure S6: DFT optimisation of a trimeric model of **PgBT(F)2gTT** at the B3LYP/6-31G(d,p) level showing backbone planarity. Hydrogen atoms omitted for clarity.

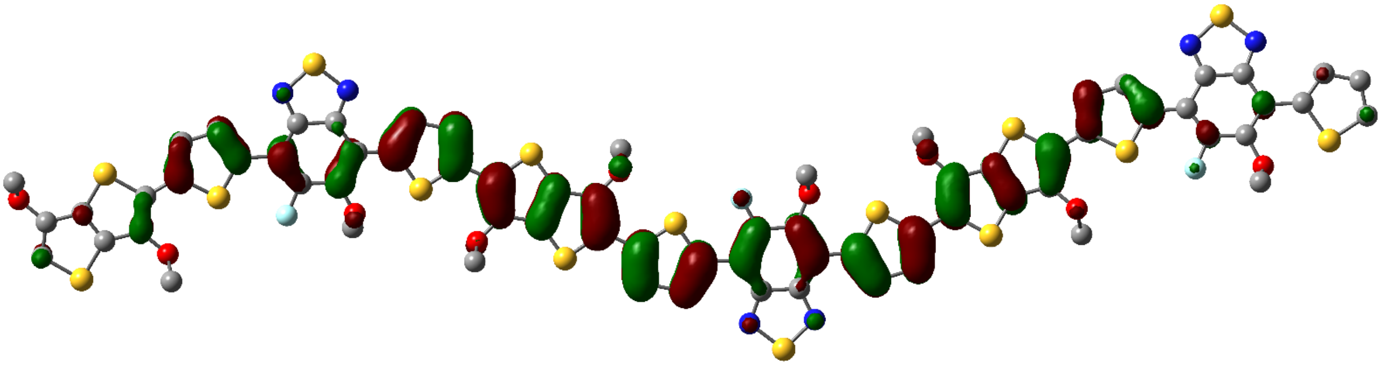

Figure S7: DFT optimisation of a trimeric model of **PgBT(F)2gTT** at the B3LYP/6-31G(d,p) level showing HOMO distribution along backbone. Hydrogen atoms omitted for clarity. **PgBT(F)2gTT** HOMO = -4.65 eV.

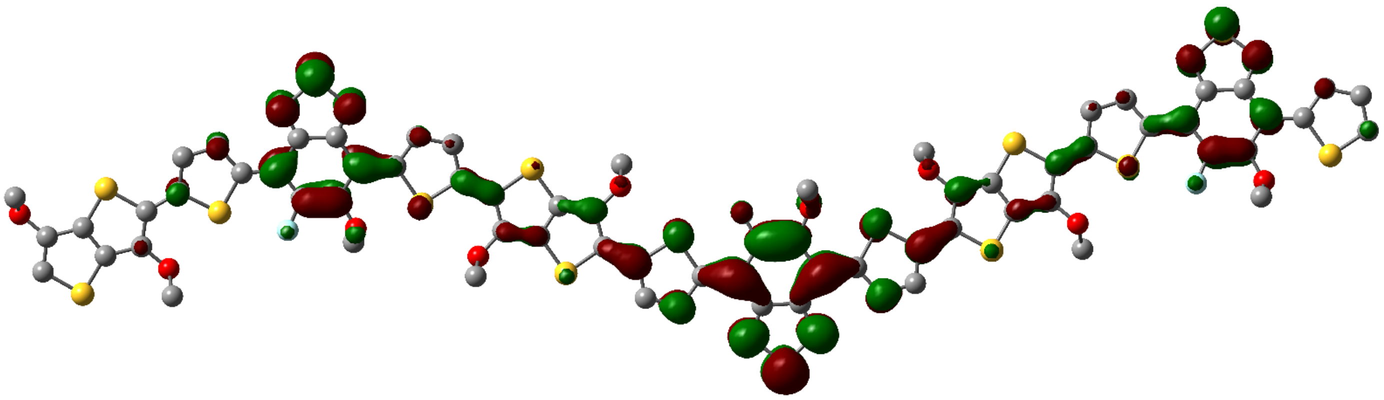

Figure S8: DFT optimisation of a trimeric model of **PgBT(F)2gTT** at the B3LYP/6-31G(d,p) level showing LUMO distribution along backbone. Hydrogen atoms omitted for clarity. **PgBT(F)2gTT** LUMO = -2.74 eV.

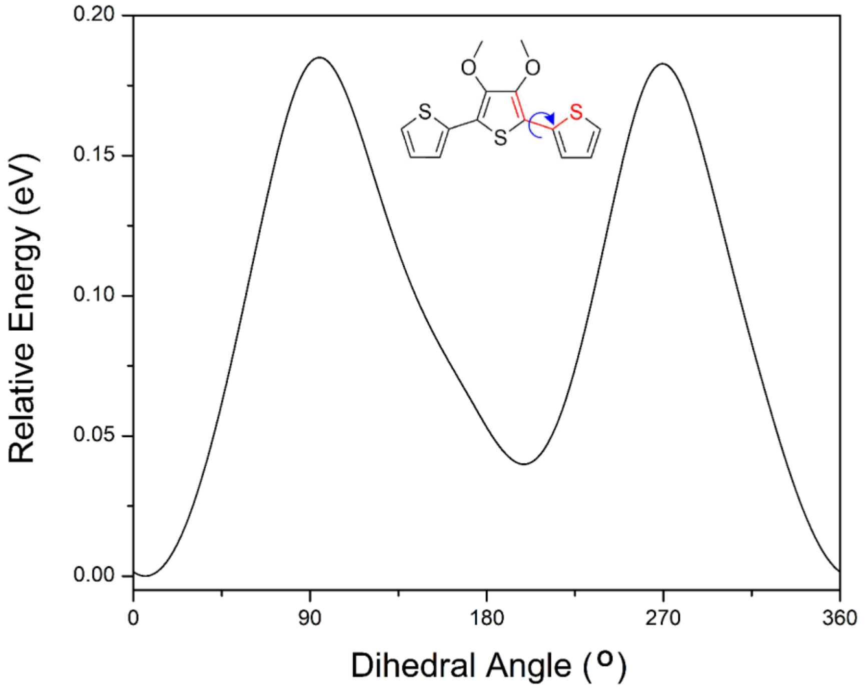

Figure S9: DFT calculated (B3LYP/6-31G(d,p) level) dihedral angle scanning potential energy surface plot of a bisthiophene-dimethoxythiophene segment found in **PgBT(F)2gT**. Dihedral angle was scanned at 1° intervals for one bond connecting the central dimethoxythiophene with a flanking thiophene heterocycle (see inset diagram). Lowest overall potential energy was found to occur at a dihedral angle of 6°, with thiophenes configured *anti*.

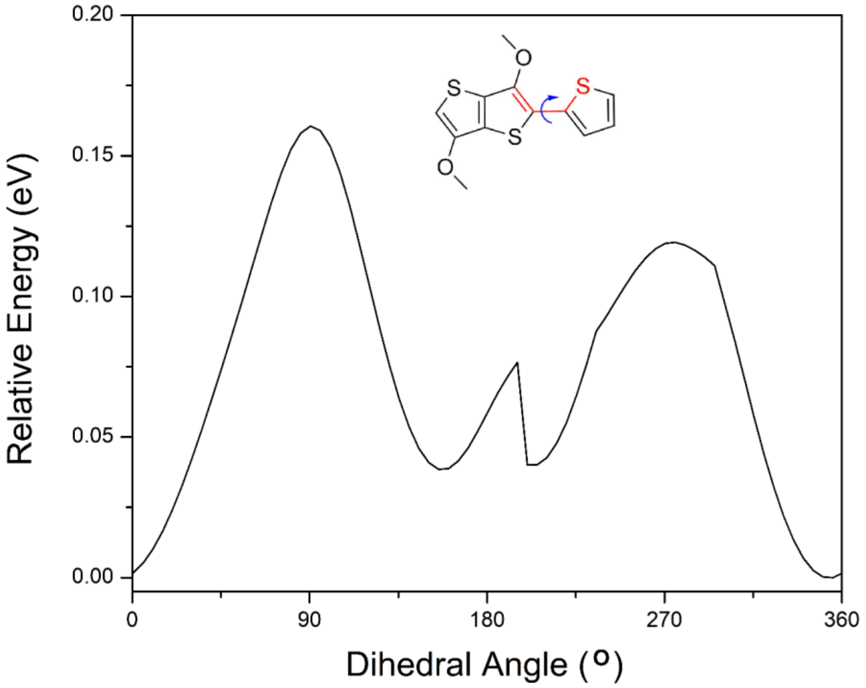

Figure S10: DFT calculated (B3LYP/6-31G(d,p) level) dihedral angle scanning potential energy surface plot of a thiophene-dimethoxythienothiophene segment found in **PgBT(F)2gTT**. Dihedral angle was scanned at 3° intervals for bond connecting dimethoxythienothiophene with thiophene (see inset diagram). Lowest overall potential energy was found to occur at a dihedral angle of 6°, with an *anti* thiophene-thienothiophene configuration.

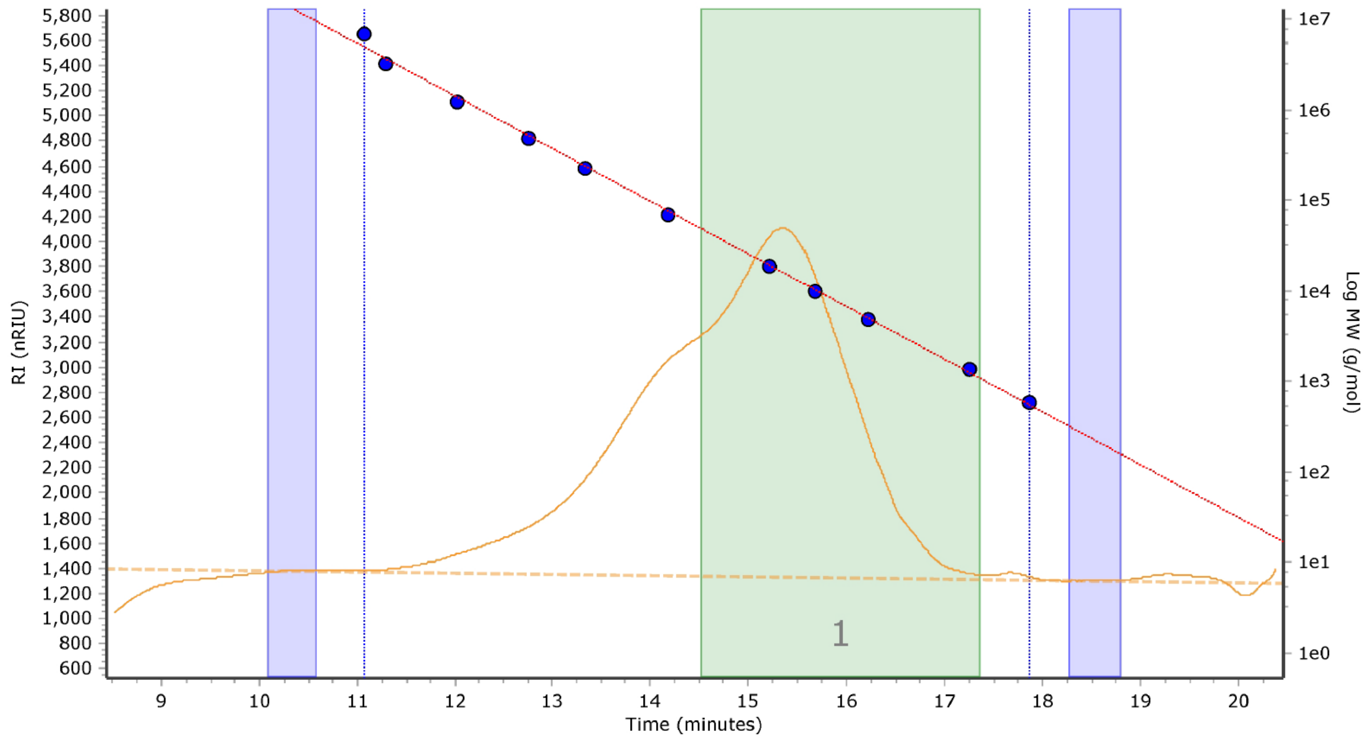

Figure S11: GPC trace of **PgBT(F)2gT** at 313 K in $CHCl_3$ at a sample concentration of 1 mg/mL. Note the bimodal elution peak attributed to aggregation (green highlighted area integrated to estimate M_n, M_w and Đ). Aggregation of **PgBT(F)2gT** in $CHCl_3$ was confirmed by re-running GPC analysis at lower sample concentrations (Figure S12) and solution state UV-Vis.

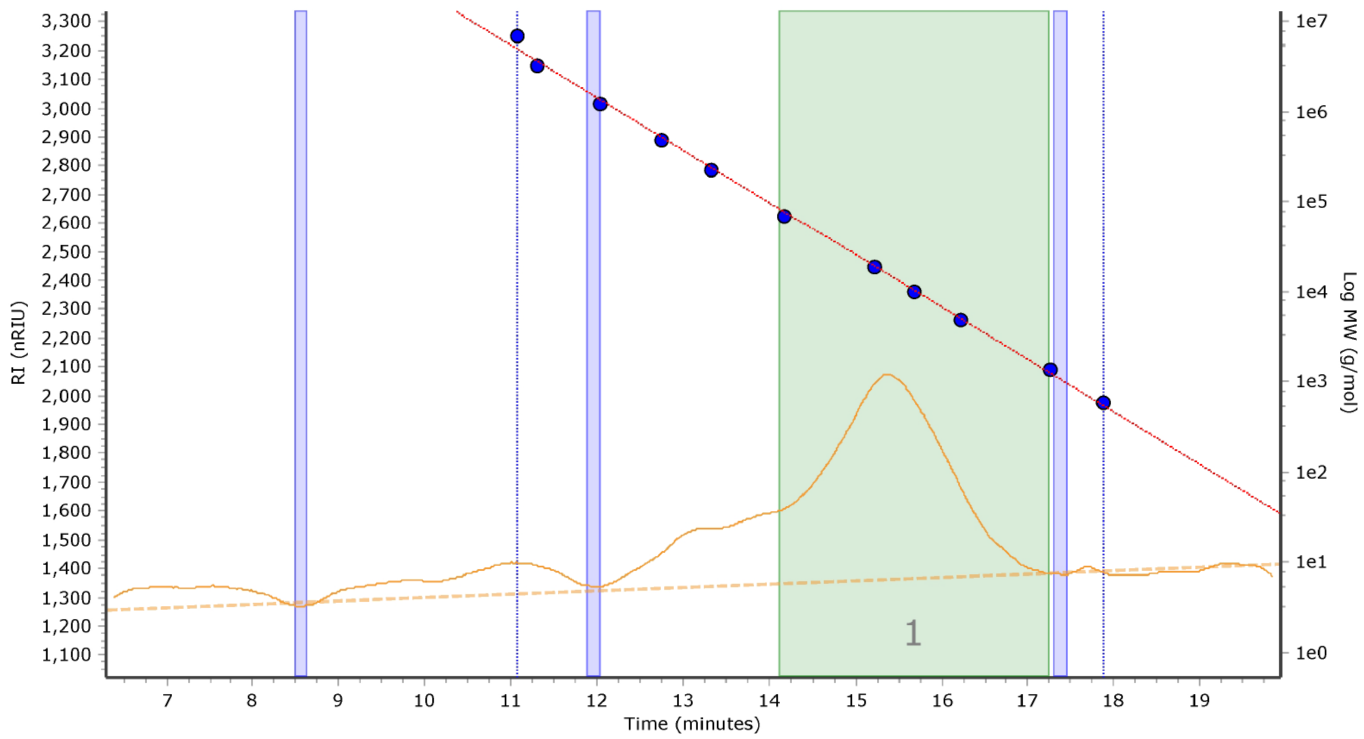

Figure S12: GPC trace of **PgBT(F)2gT** at 313 K in $CHCl_3$ at a sample concentration of 0.4 mg/mL. Aggregation of **PgBT(F)2gT** was confirmed by the change in bimodal peak ratio, compared to the GPC trace shown in Figure S11 corresponding to 1 mg/mL sample concentration.

(a)

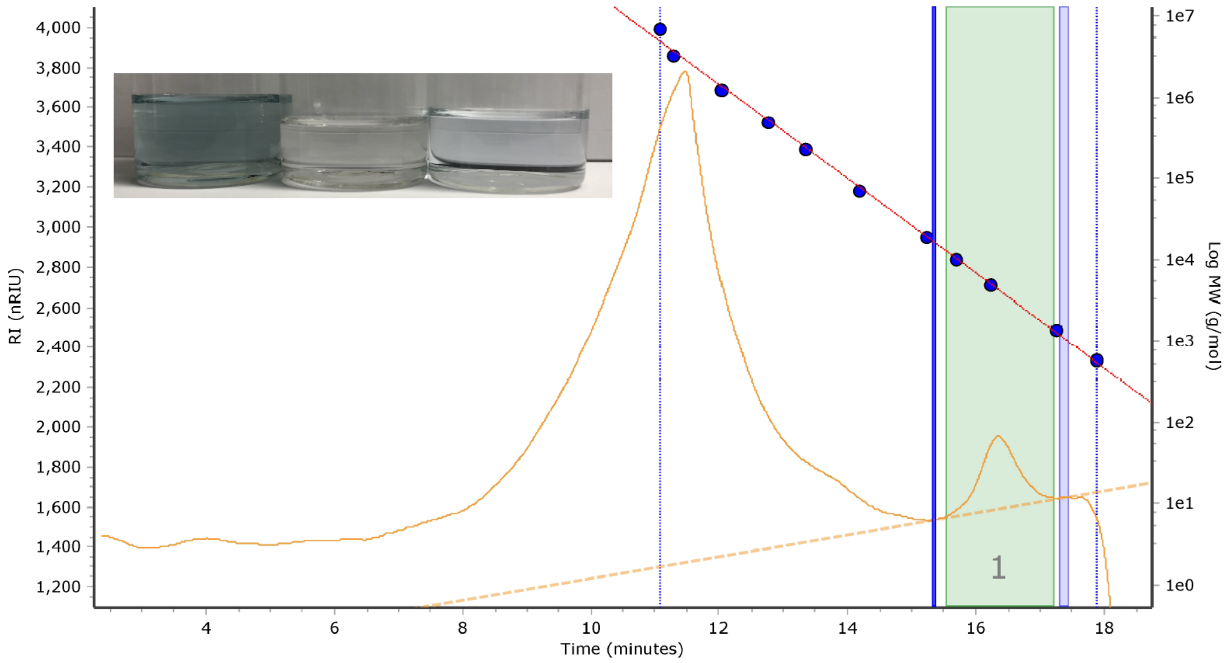

(b)

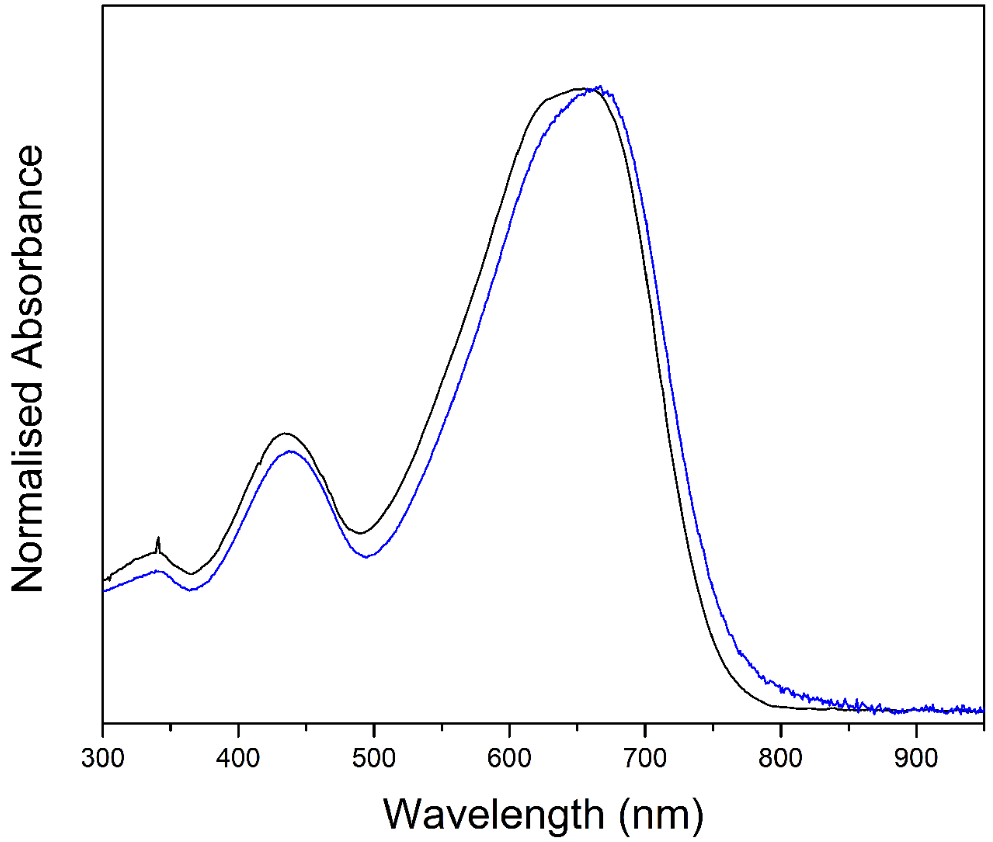

Figure S13: (a) GPC trace of **PgBT(F)2gTT** at 313 K in $CHCl_3$ at a sample concentration of 1 mg/mL (green highlighted area integrated to estimate M_n, M_w and Đ). The large peak at 12 mins was attributed to aggregated **PgBT(F)2gTT** in $CHCl_3$, which was (b) confirmed by its $CHCl_3$ solution state UV-Vis (blue), as compared to that of a stock solution of **PgBT(F)2gTT** (black). The inset image in (a) shows fractions collected from the GPC; the blue

fraction on the left correspond to the large peak at 12 min, the clear middle fraction correspond to blank elute at 4 min and the light blue fraction on the right correspond to the peak at 17 min.

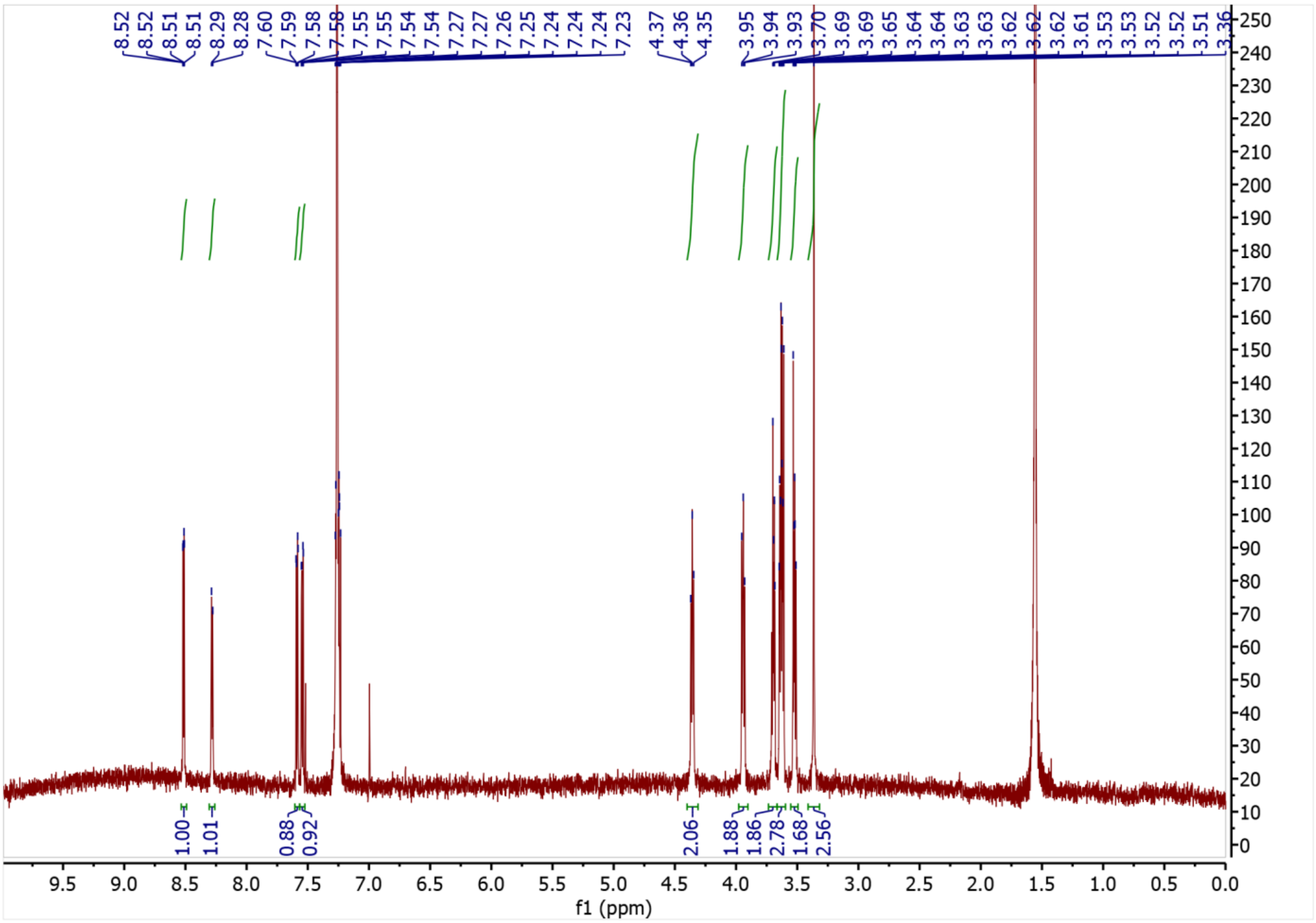

Figure S14: ^{1}H NMR of compound **4** at 298 K.

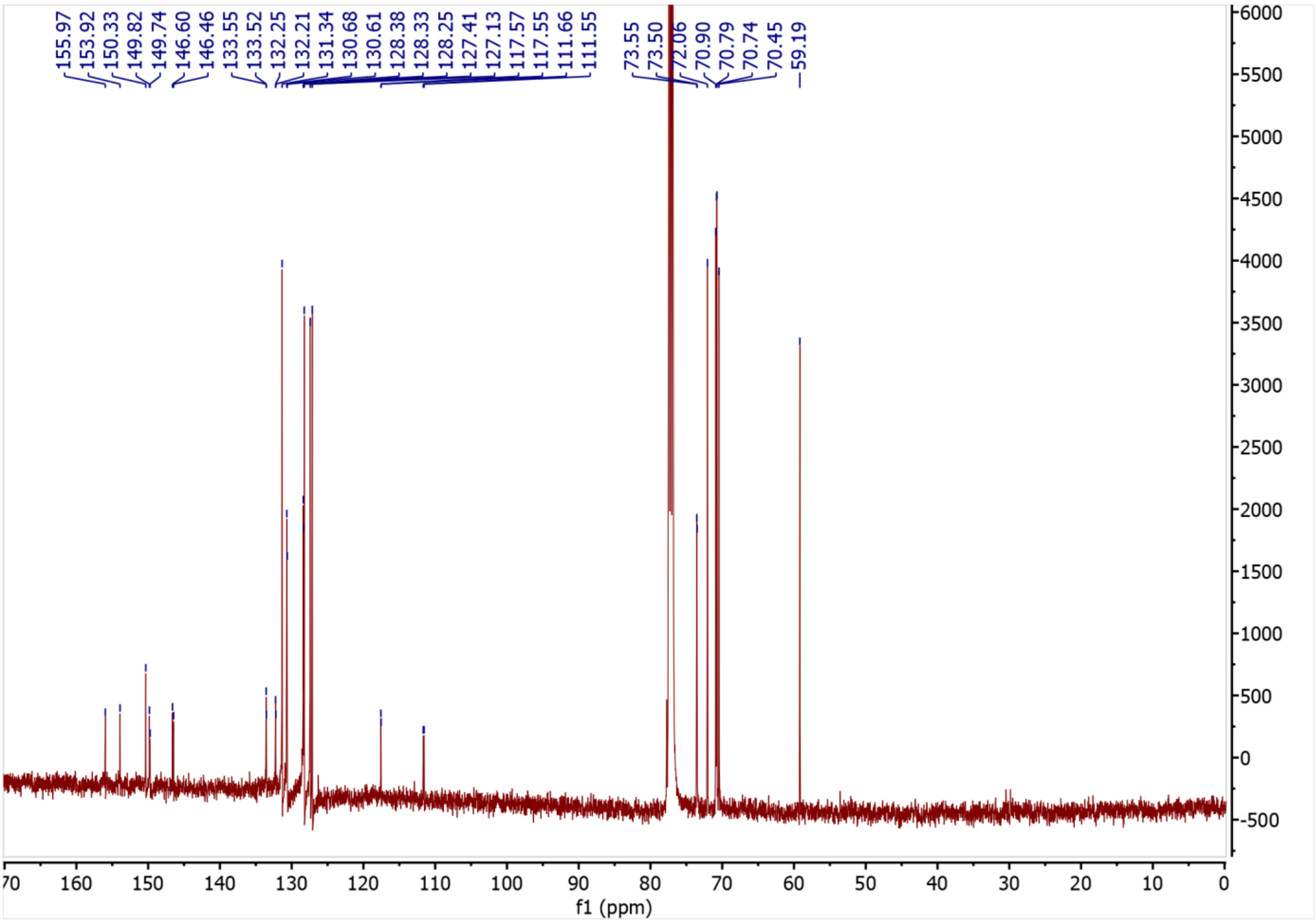

Figure S15: $^{13}C\{^{1}H\}$ NMR of compound **4** at 298 K.

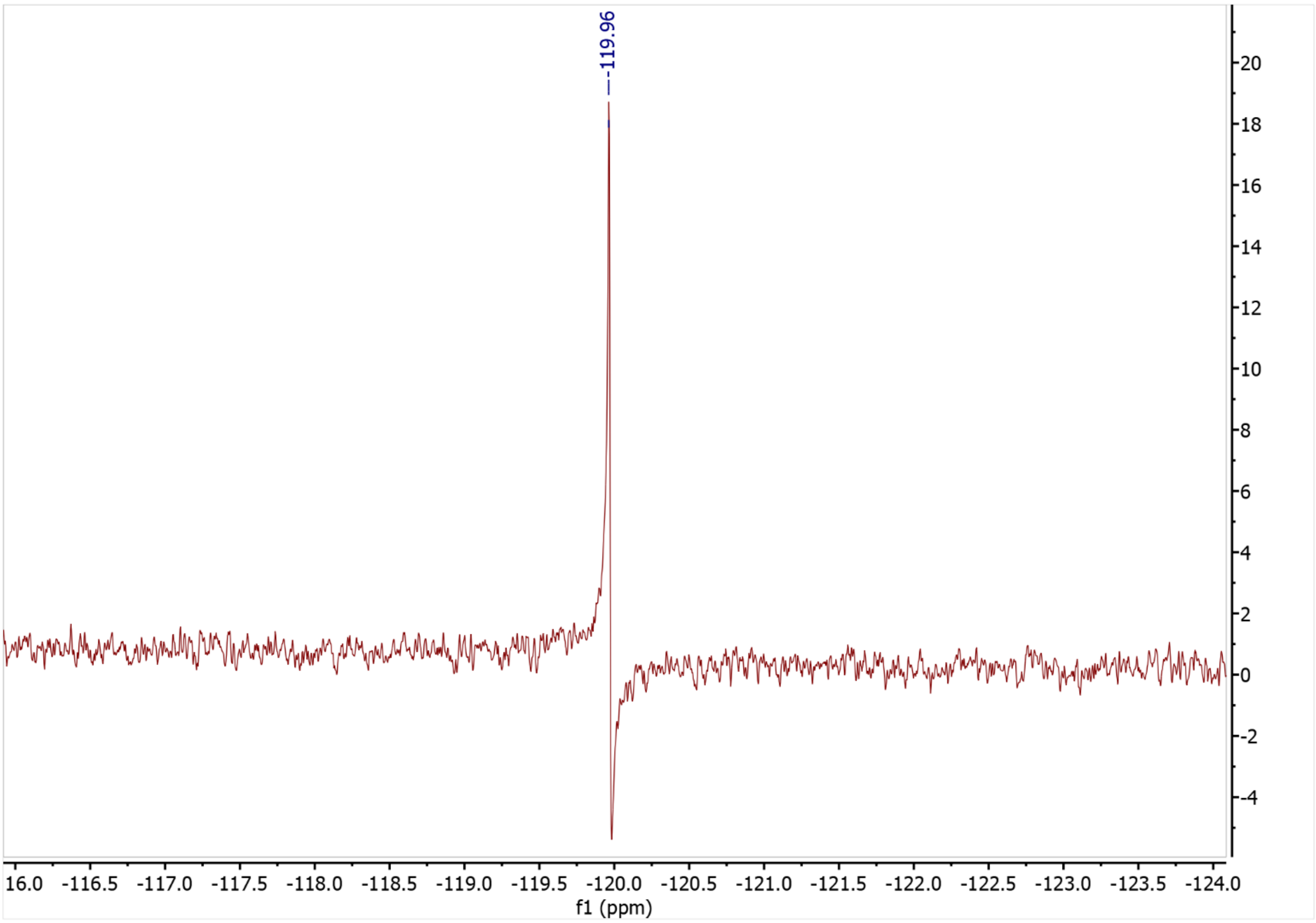

Figure S16: ^{19}F NMR of compound **4** at 298 K.

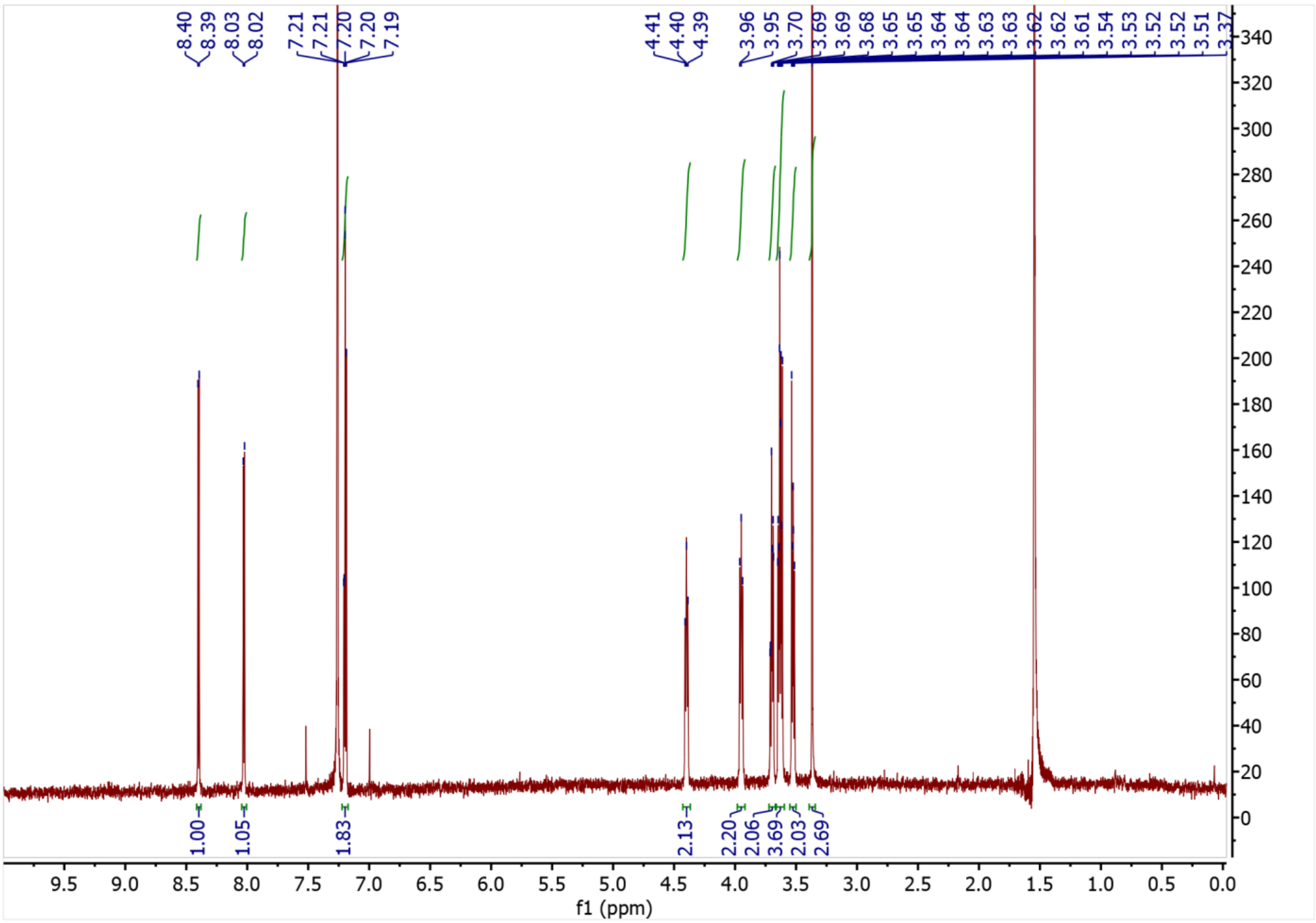

Figure S17: ^{1}H NMR of compound **1** at 298 K.

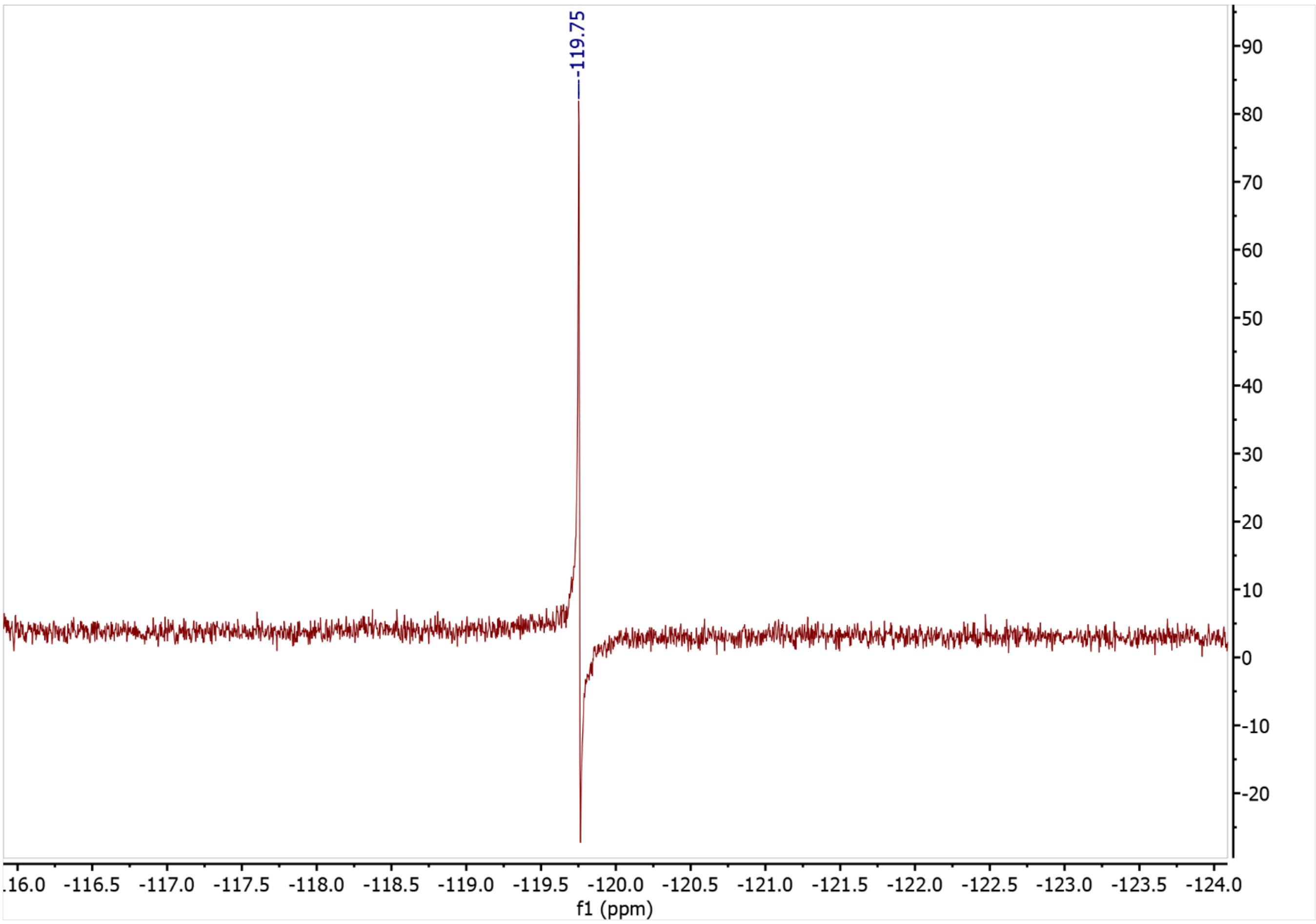

Figure S18: ^{19}F NMR of compound **1** at 298 K.

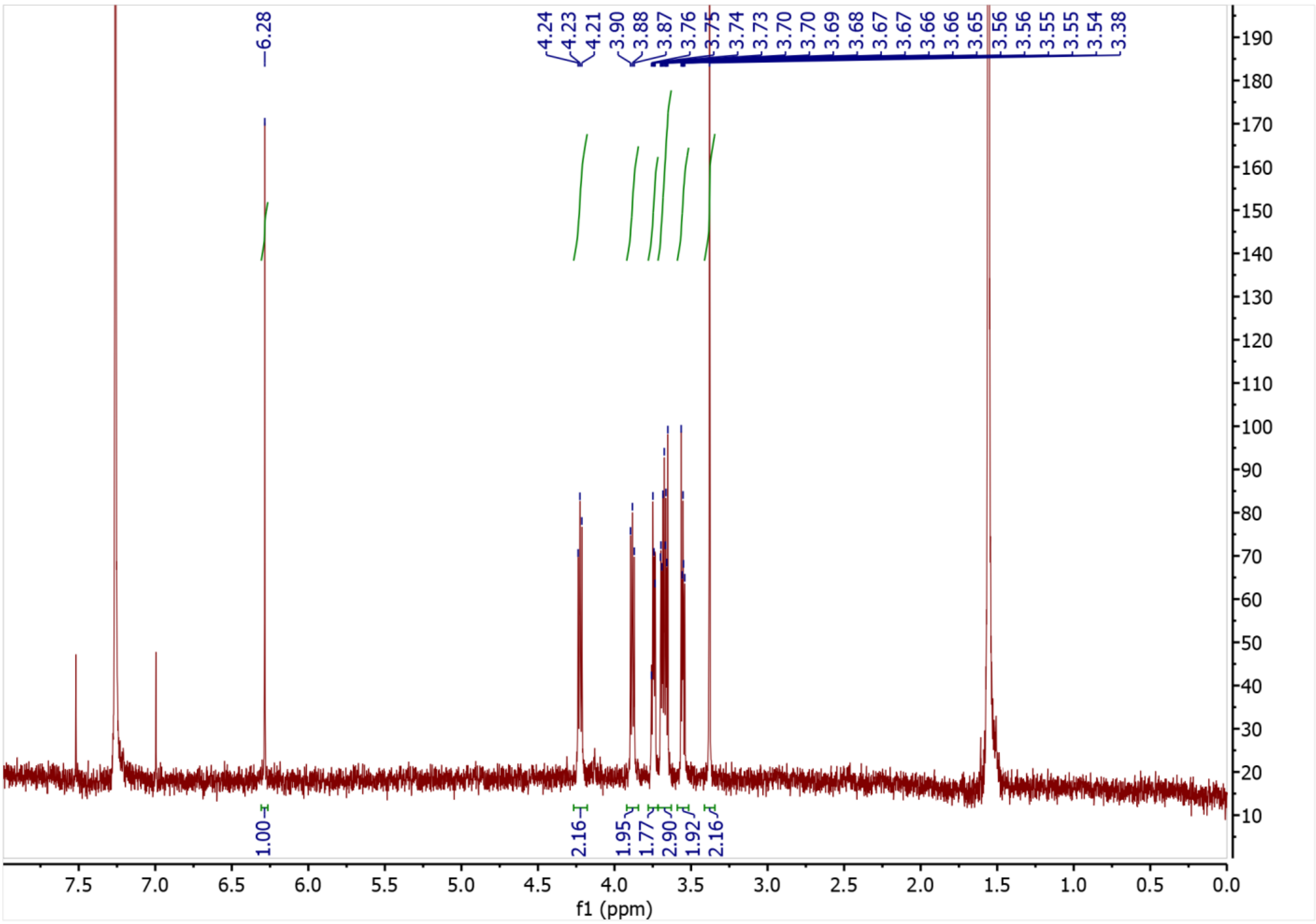

Figure S19: ^{1}H NMR of compound **2** at 298 K.

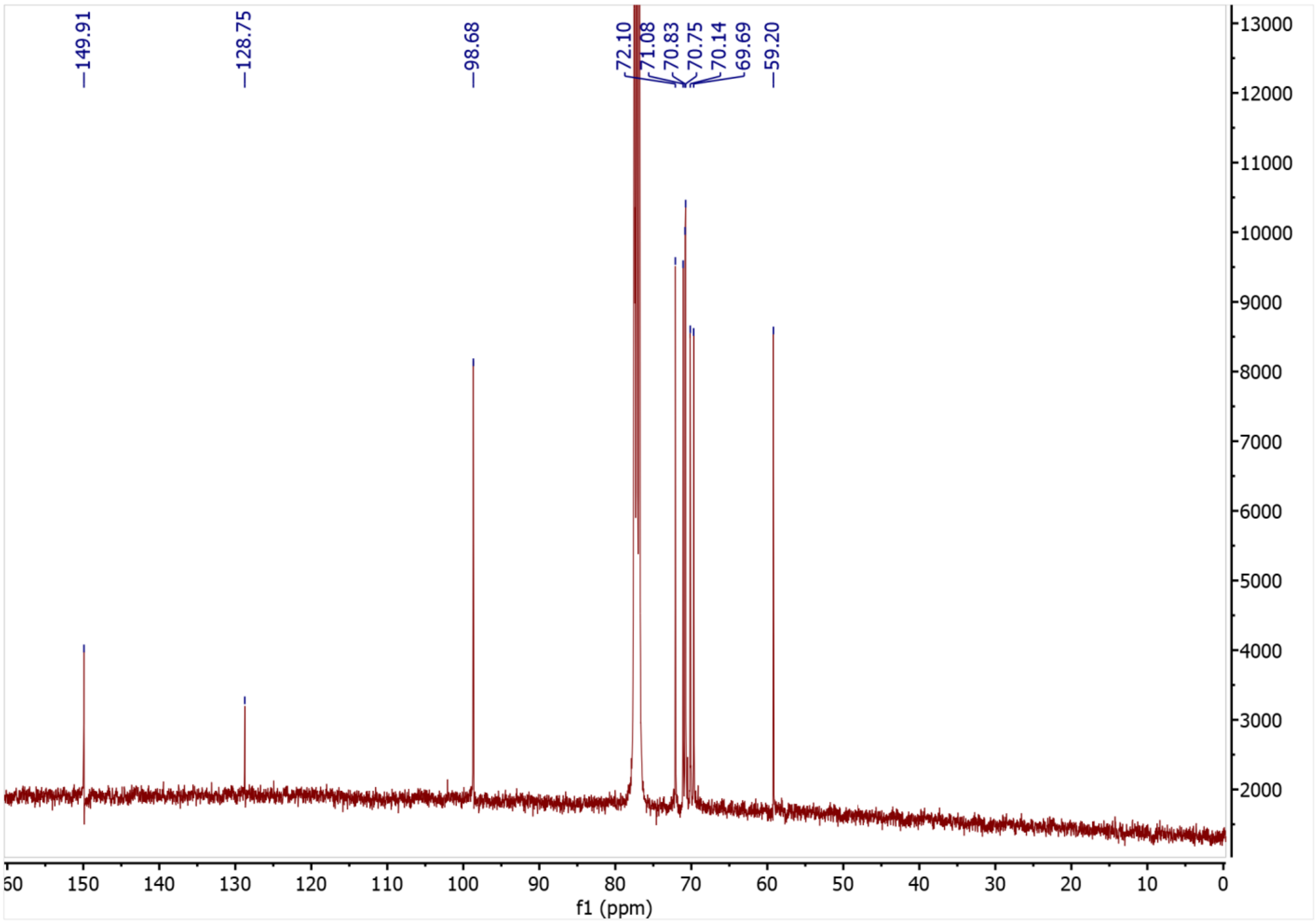

Figure S20: ^{13}C{^{1}H} NMR of compound **2** at 298 K.

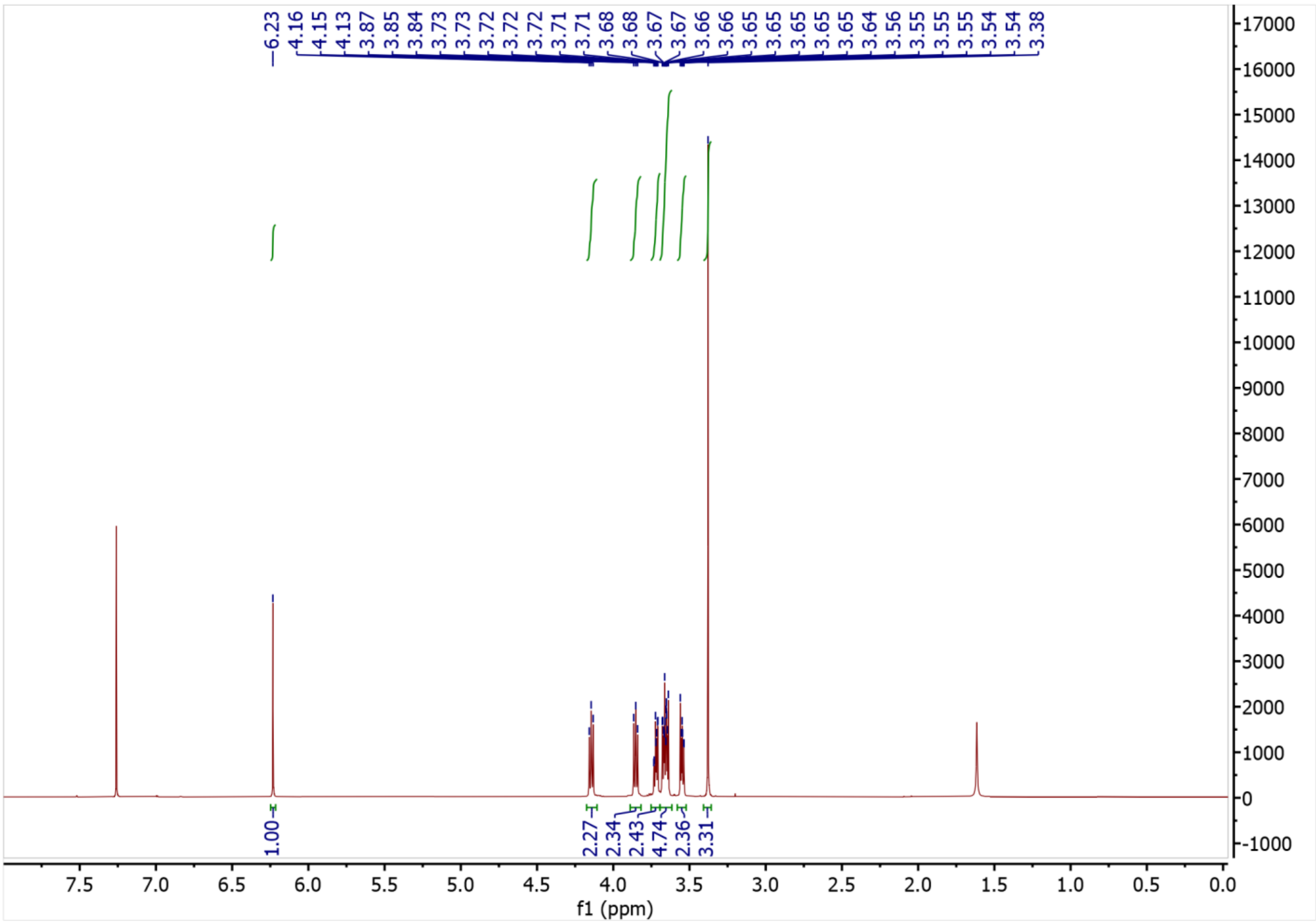

Figure S21: ^{1}H NMR of compound **3** at 298 K.

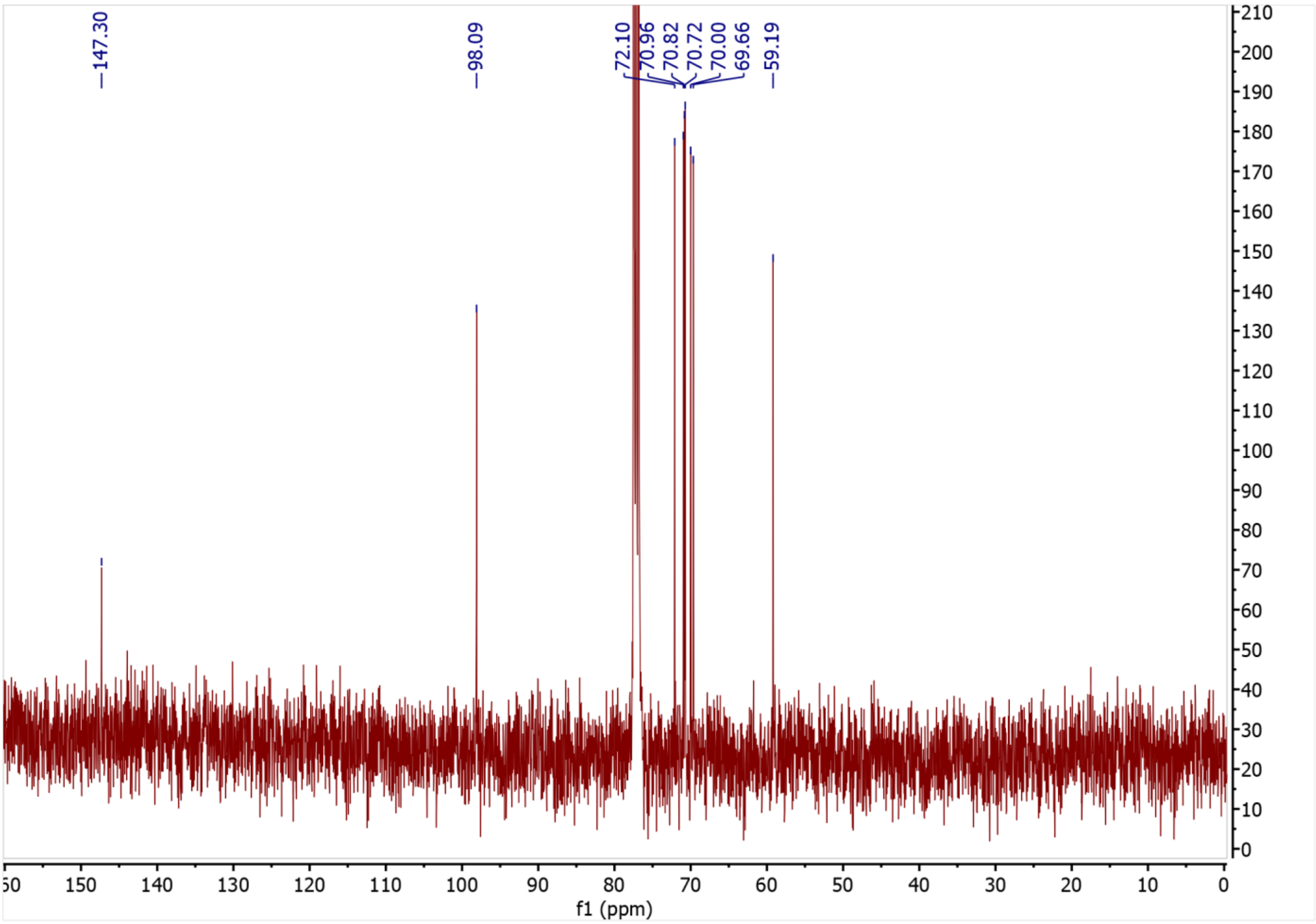

Figure S22: $^{13}C\{^{1}H\}$ NMR of compound **3** at 298 K.

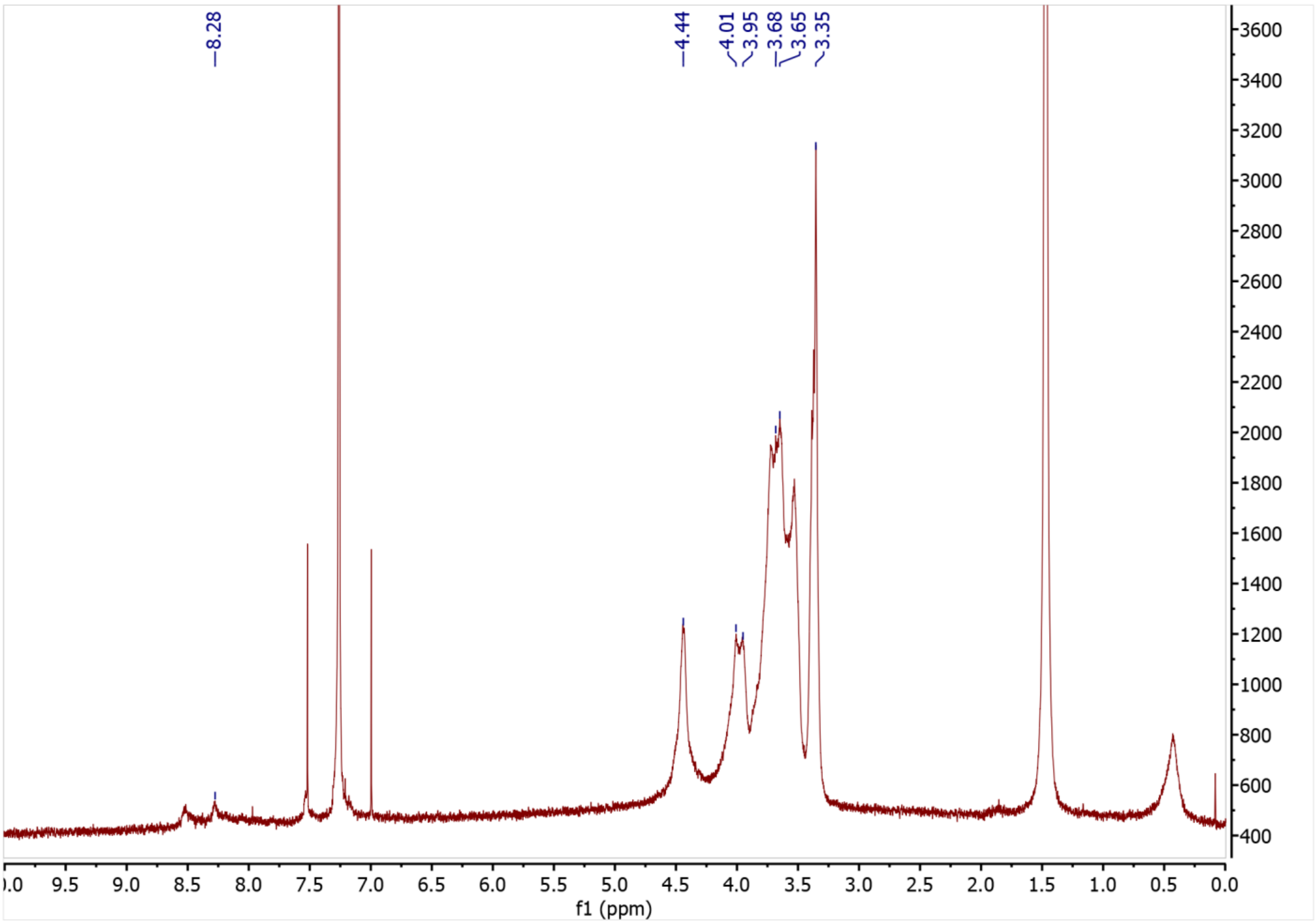

Figure S23: ^{1}H NMR of **PgBT(F)2gT** at 328 K.

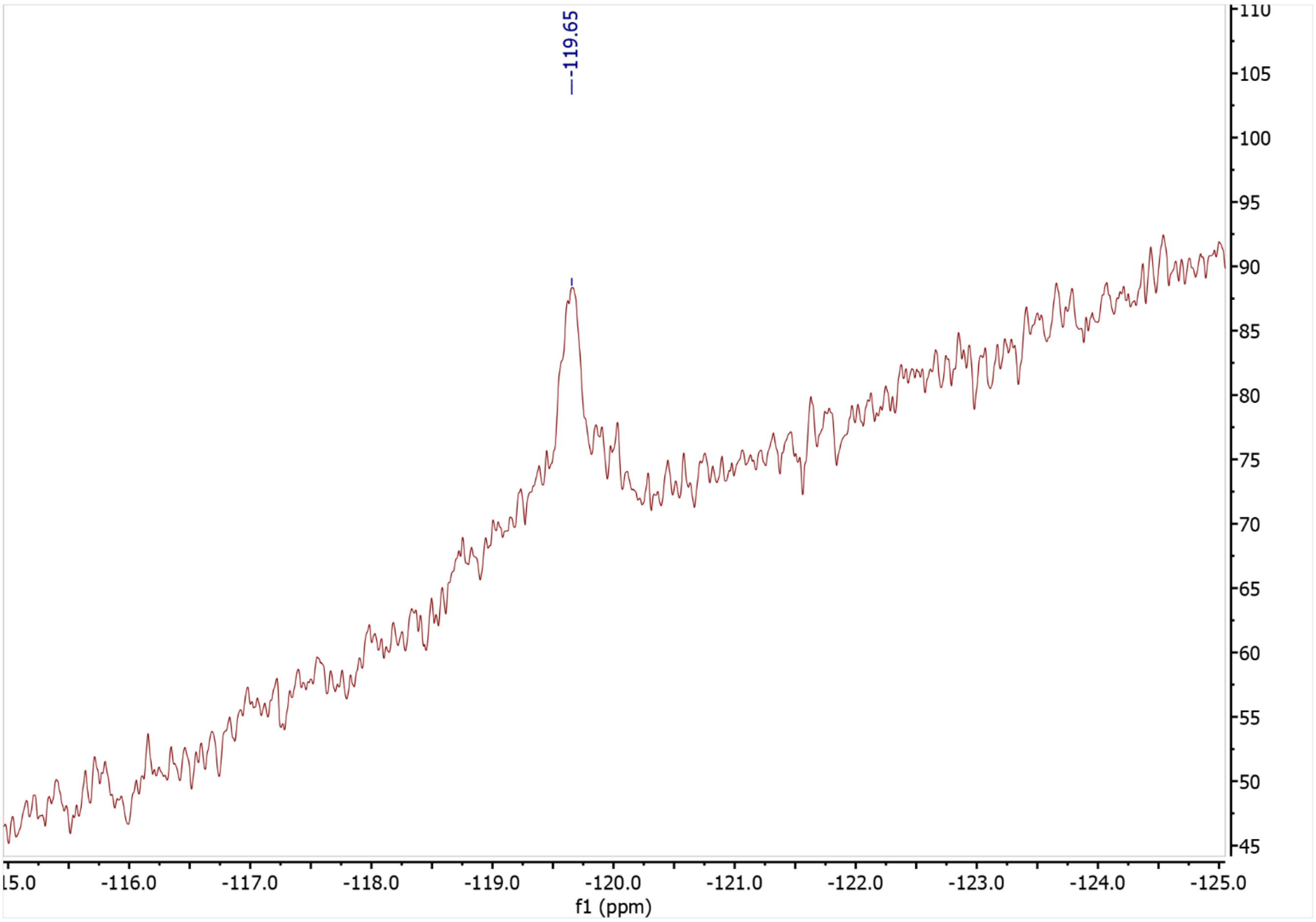

Figure S24: ^{19}F NMR of **PgBT(F)2gT** at 328 K.

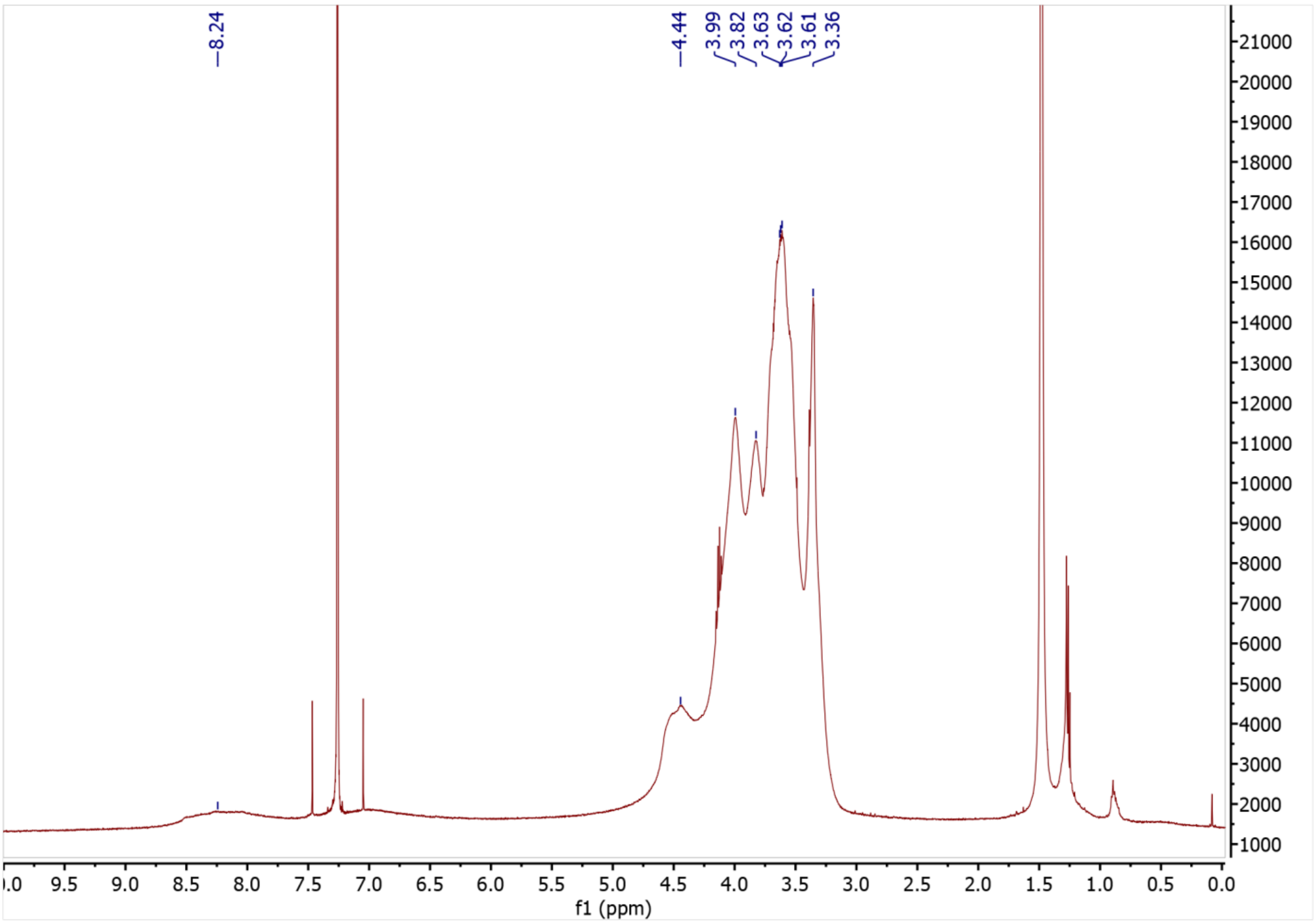

Figure S25: ^{1}H NMR of **PgBT(F)2gTT** at 328 K.

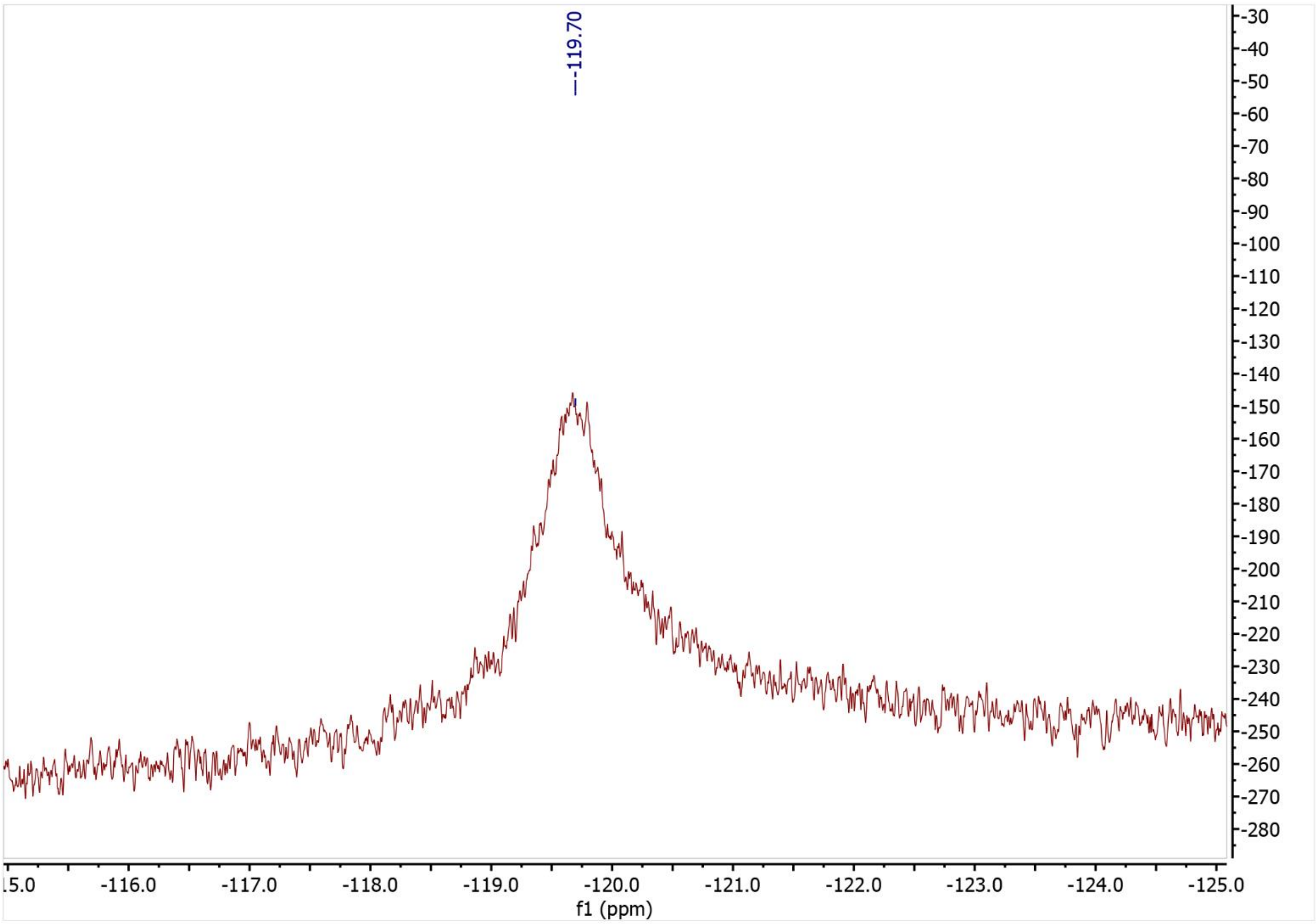

Figure S26: ^{19}F NMR of **PgBT(F)2gTT** at 328 K.

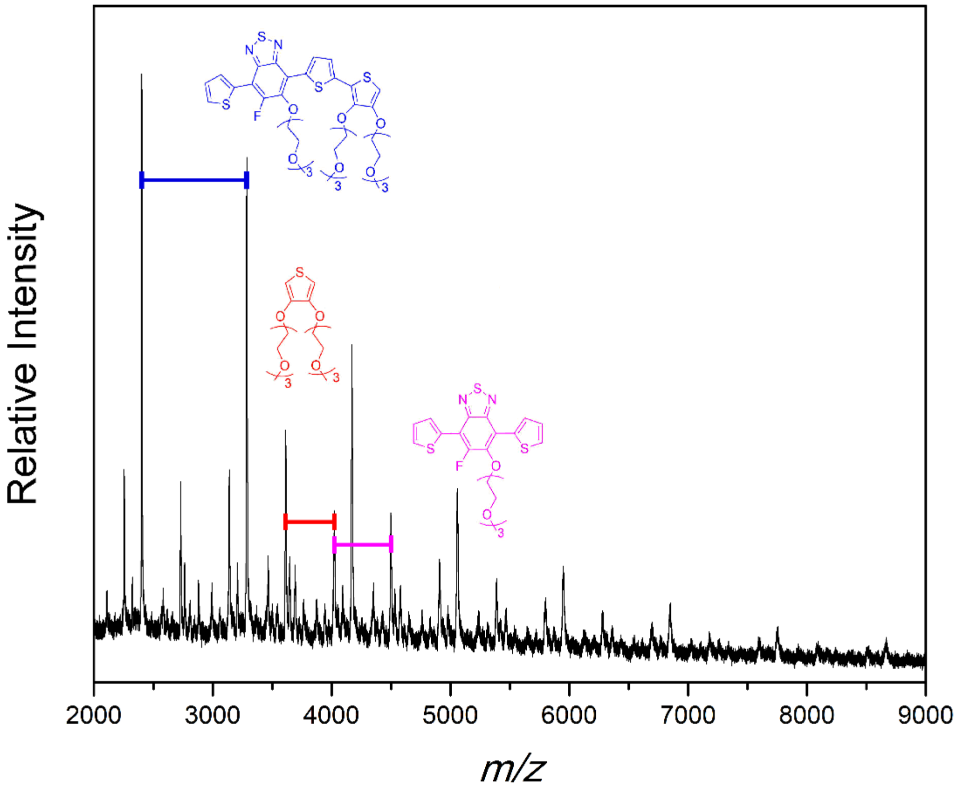

Figure S27: MALDI-ToF of **PgBT(F)2gT**.

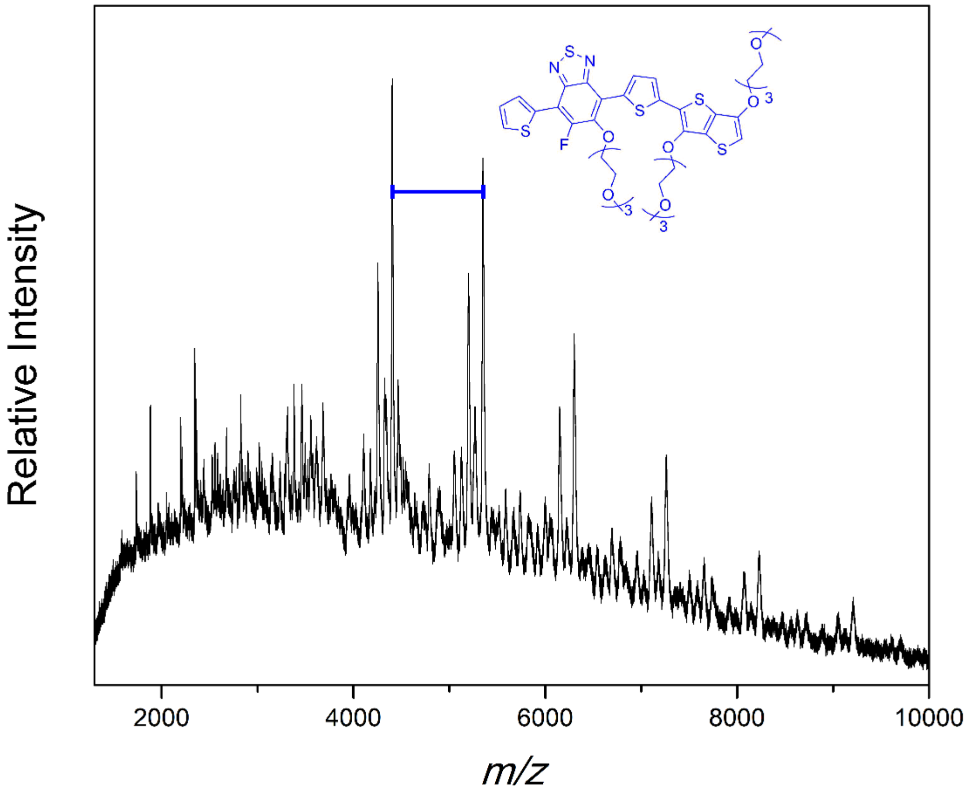

Figure S28: MALDI-ToF of **PgBT(F)2gTT**.

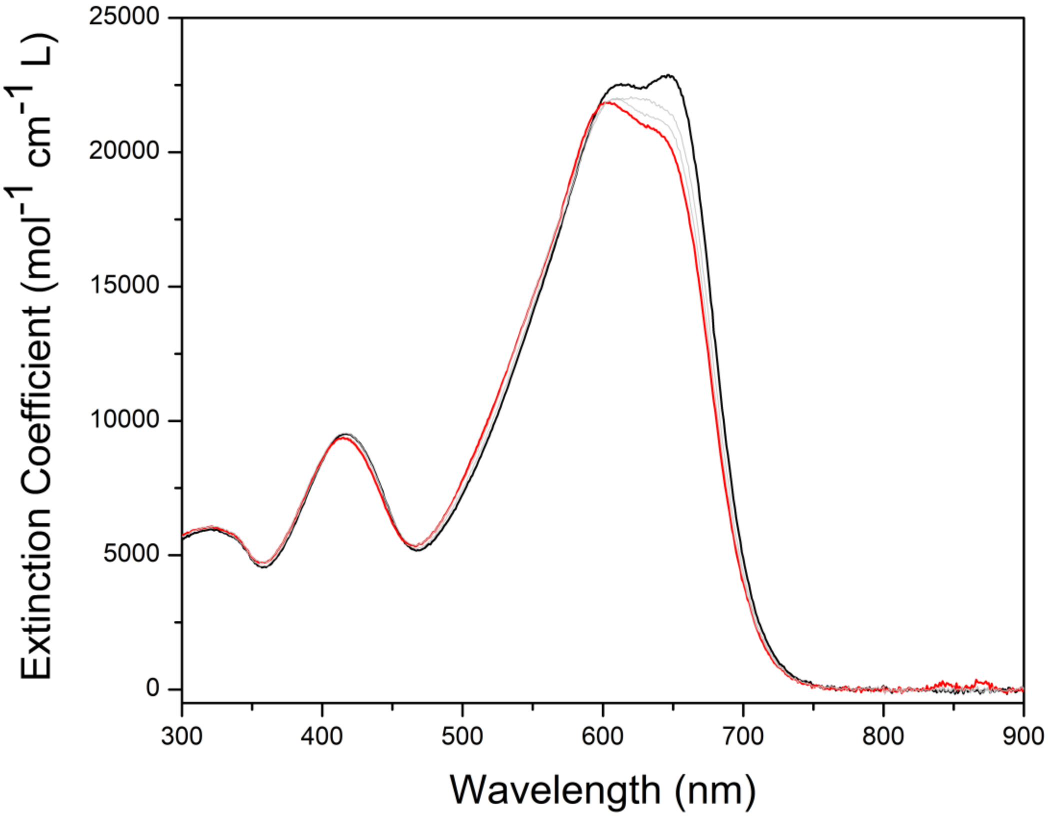

Figure S29: Solution state UV-Vis of **PgBT(F)2gT** in $CHCl_3$ at RT (black) and upon heating to *ca.* 50 °C (red).

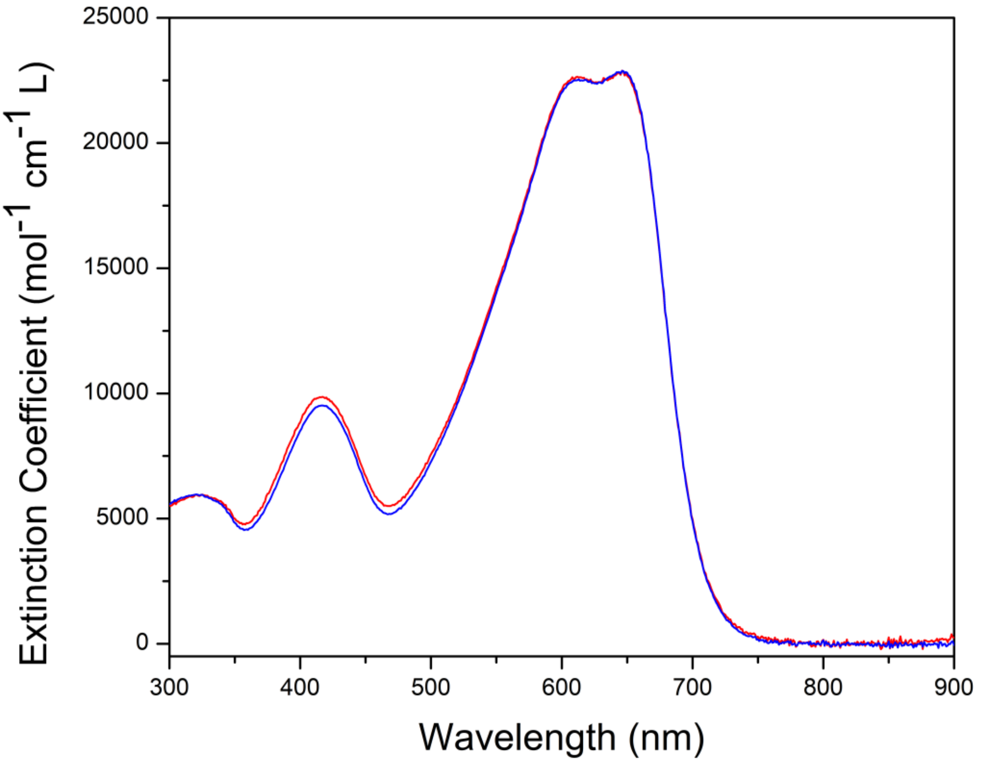

Figure S30: Solution state UV-Vis of **PgBT(F)2gT** in $CHCl_3$ (red), compared to double concentration (blue).

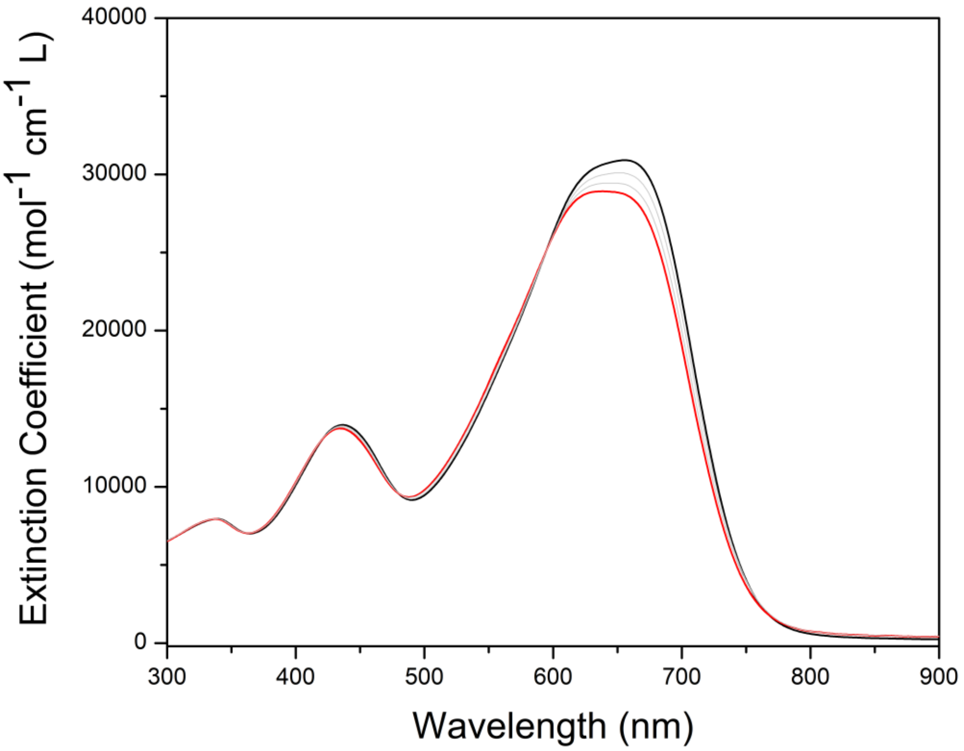

Figure S31: Solution state UV-Vis of **PgBT(F)2gTT** in $CHCl_3$ at RT (black) and upon heating to *ca.* 50 °C (red).

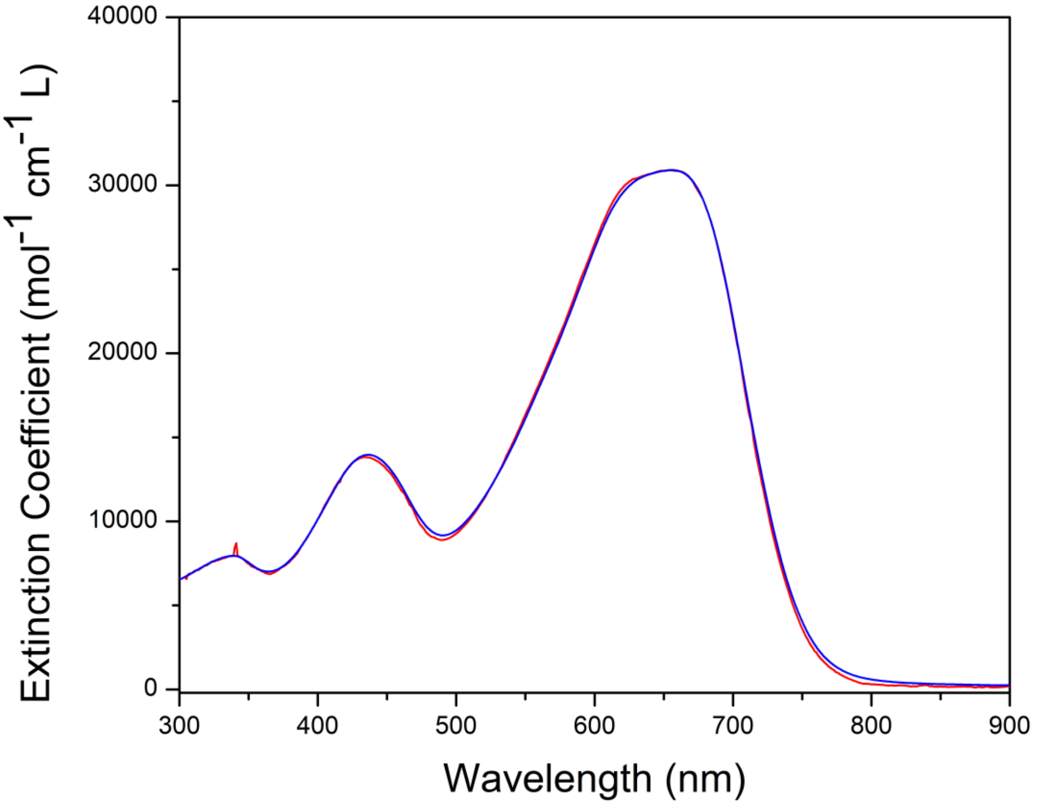

Figure S32: Solution state UV-Vis of **PgBT(F)2gTT** in $CHCl_3$ (red), compared to double concentration (blue).

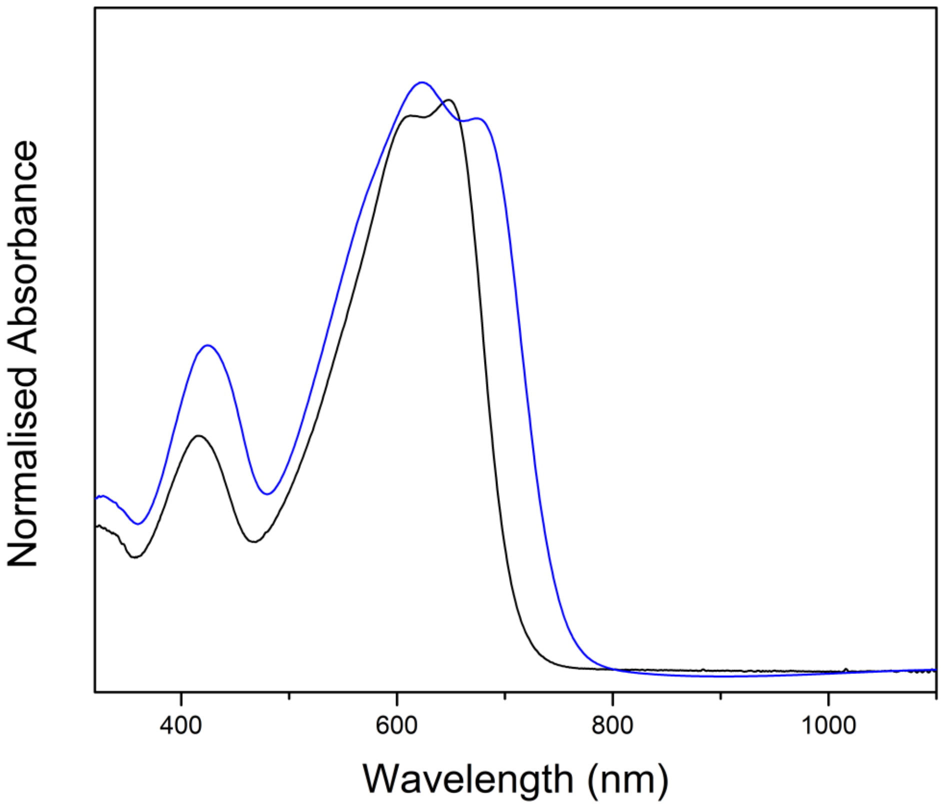

Figure S33: $CHCl_3$ solution state (black) and solid state thin film (blue) UV-Vis of **PgBT(F)2gT**.

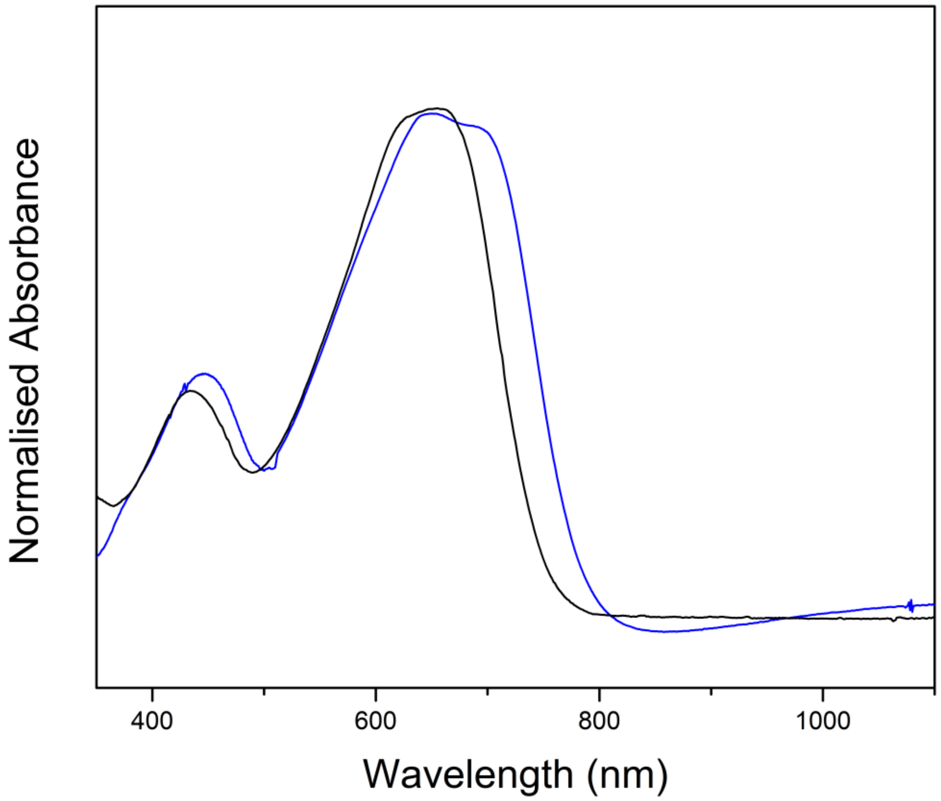

Figure S34: $CHCl_3$ solution state (black) and solid state thin film (blue) UV-Vis of **PgBT(F)2gTT**.

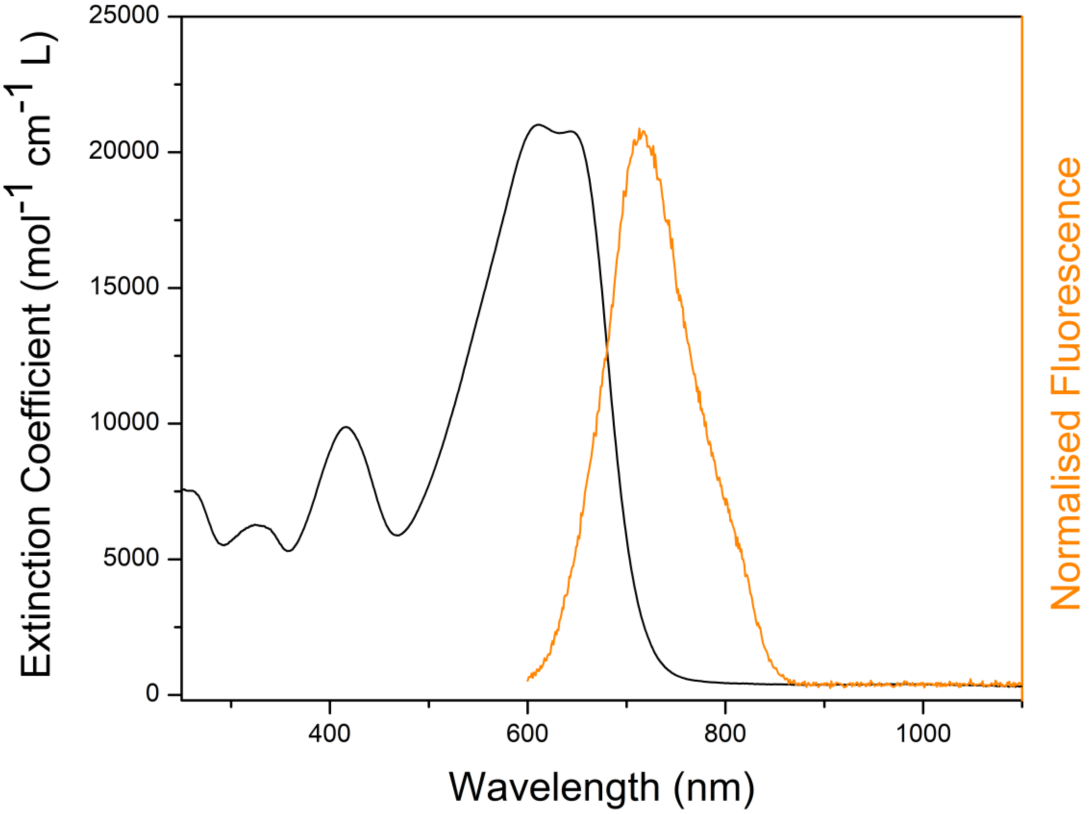

Figure S35: $CHCl_3$ solution state UV-Vis (black) and fluorescence (orange, 590 nm excitation) of **PgBT(F)2gT**.

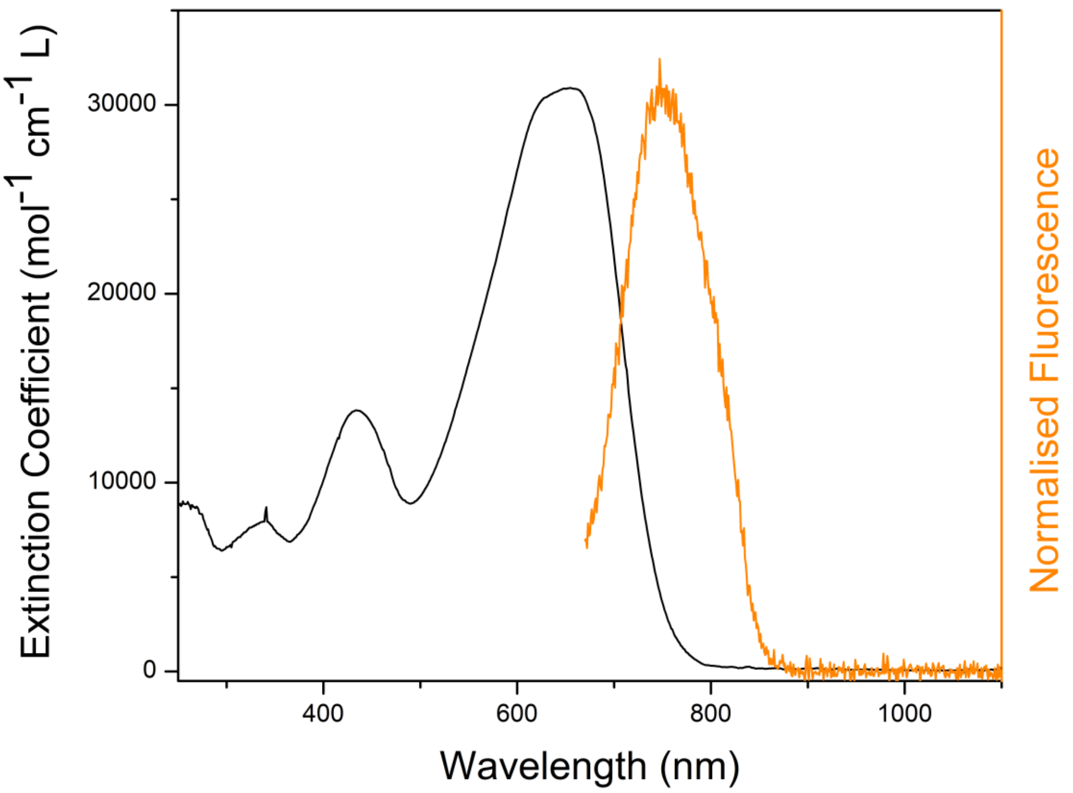

Figure S36: $CHCl_3$ solution state UV-Vis (black) and fluorescence (orange, 660 nm excitation) of **PgBT(F)2gTT**.

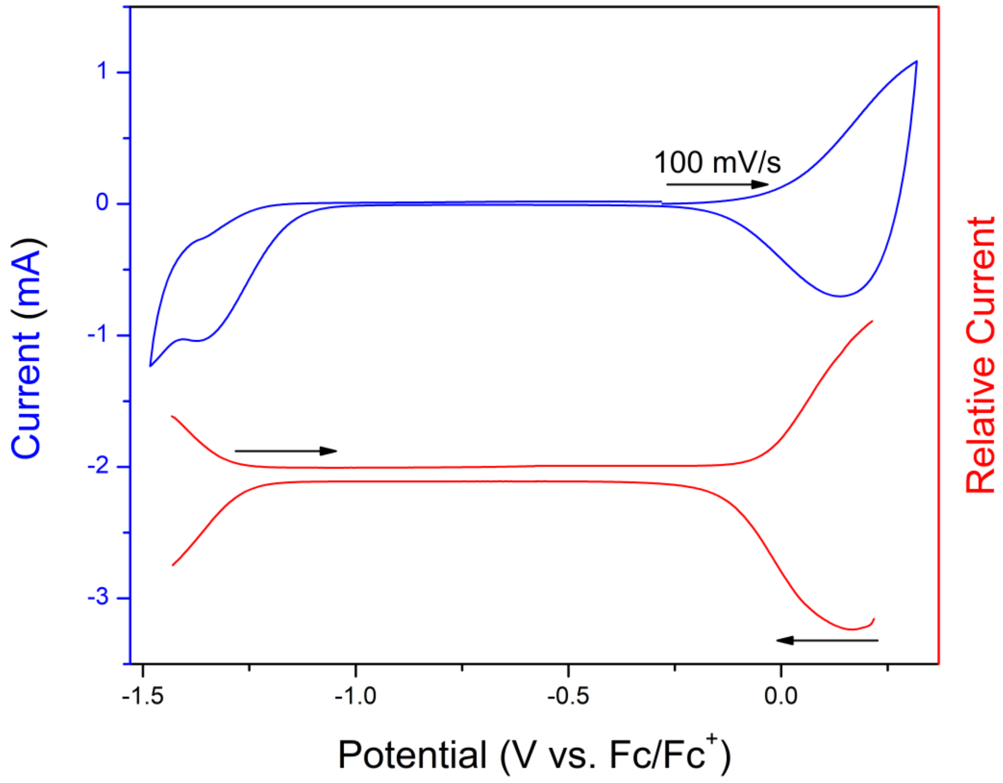

Figure S37: Solid state thin film electrochemistry of **PgBT(F)2gT** in 0.1 M [*n*-Bu_4N]PF_6/MeCN, showing CV (blue) and SQW (red) data. Arrows indicate direction of scans.

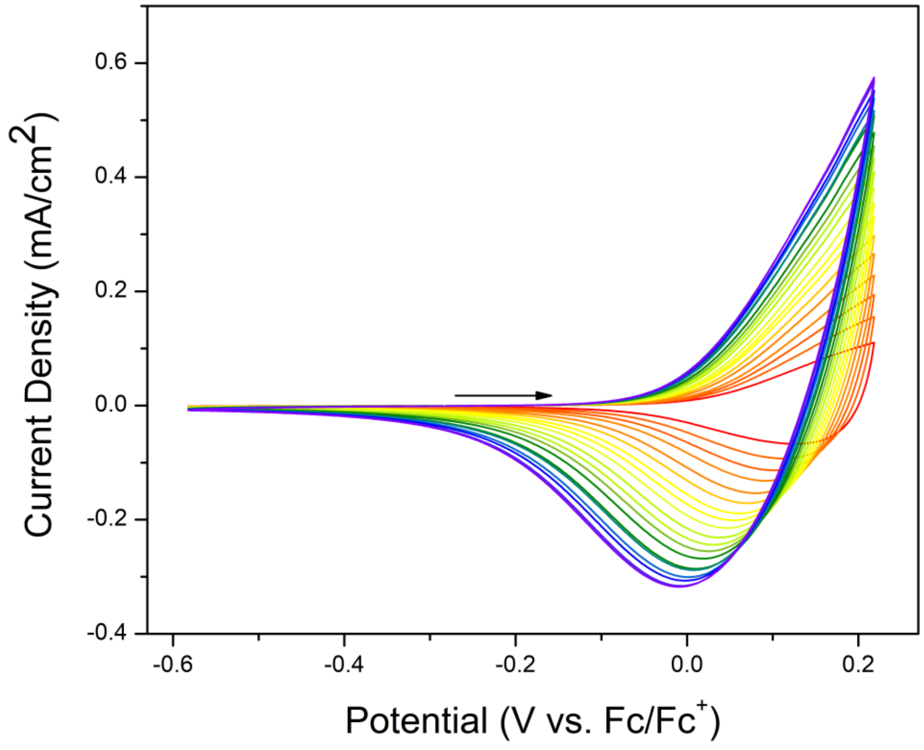

Figure S38: Scan rate dependence CV of **PgBT(F)2gT** thin films in 0.1 M [*n*-Bu_4N]PF_6/MeCN between 20 – 200 mV/s. Arrow indicates direction of scans.

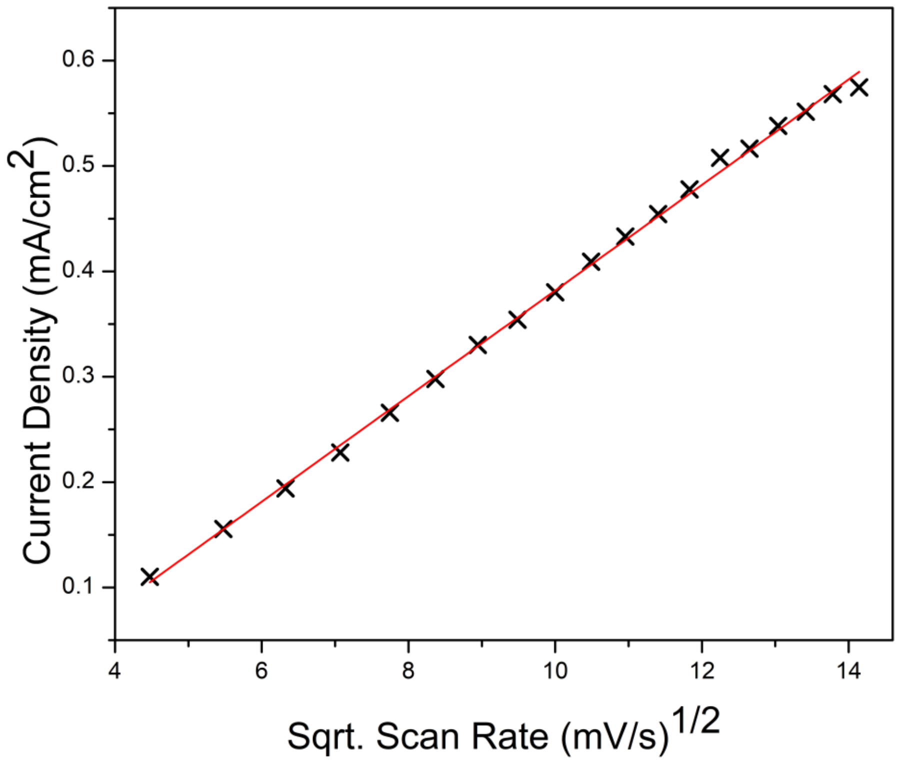

Figure S39: Plot of currents at 0.22 V *vs.* Fc/Fc$^+$ against square roots of their corresponding scan rates, for **PgBT(F)2gT** scan rate dependence CV data presented in Figure S39. Linear correlation was confirmed by regression analysis (red, R^2 = 0.99), suggesting a diffusion limited electrochemical process according to the Randles-Sevcik equation.[2]

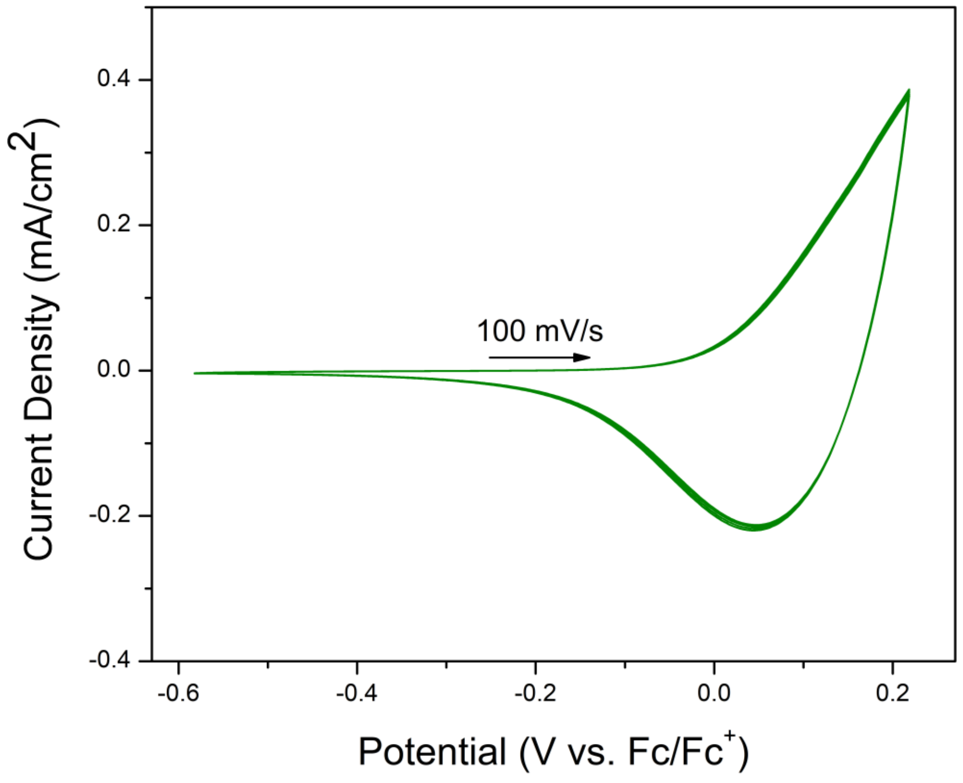

Figure S40: Cycling CV of **PgBT(F)2gT** thin films in 0.1 M [*n*-Bu_4N]PF_6/MeCN (36 cycles). Arrow indicates direction of cyclic scanning.

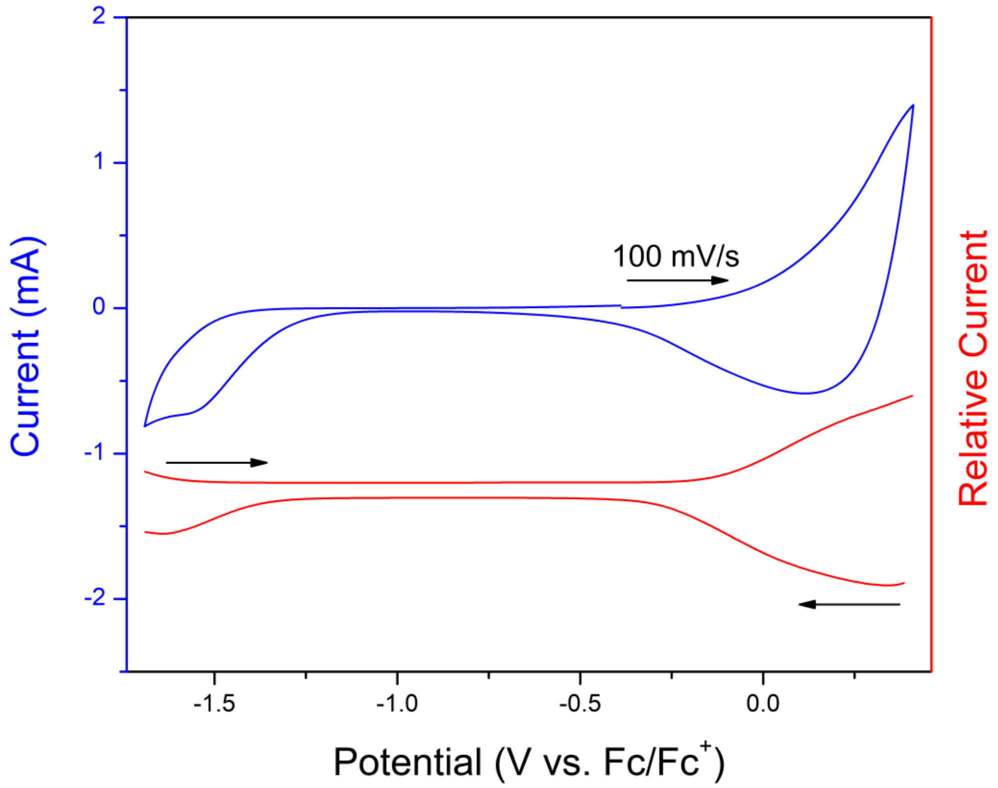

Figure S41: Solid state thin film electrochemistry of **PgBT(F)2gTT** in 0.1 M [*n*-Bu_4N]PF_6/MeCN, showing CV (blue) and SQW (red) data. Arrows indicate direction of scans.

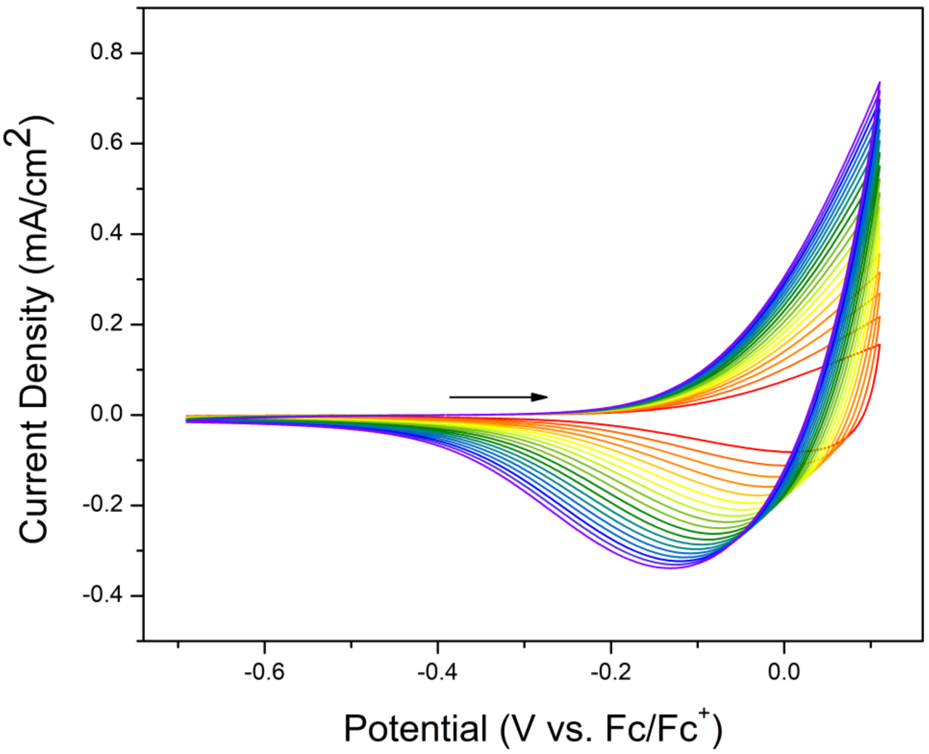

Figure S42: Scan rate dependence CV of **PgBT(F)2gTT** thin films in 0.1 M [*n*-Bu_4N]PF_6/MeCN between 20 – 200 mV/s. Arrow indicate direction of scans.

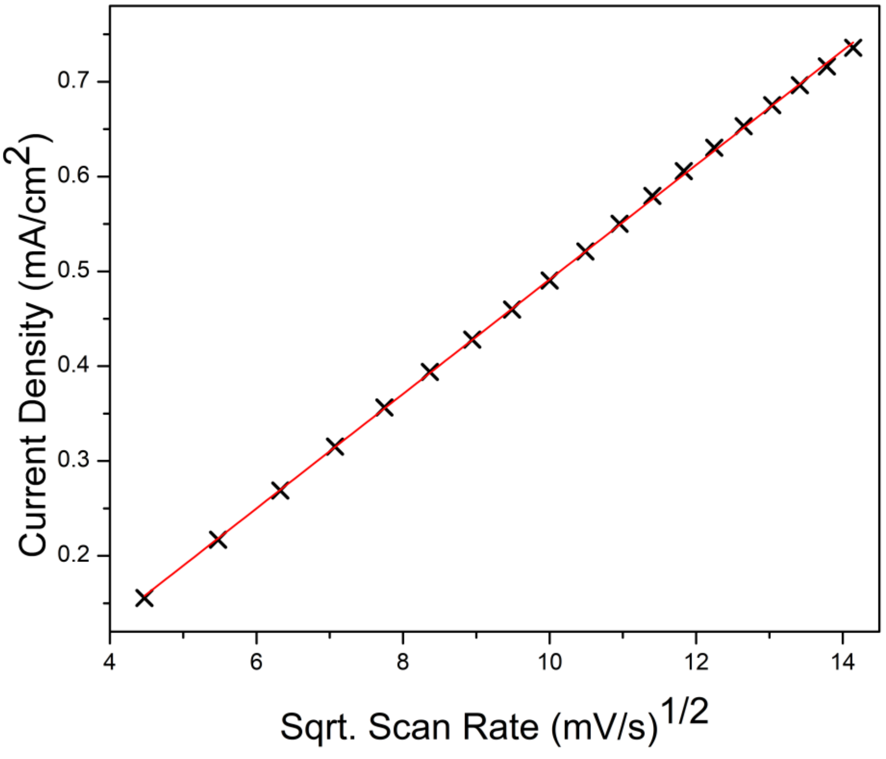

Figure S43: Plot of currents at 0.11 V *vs.* Fc/Fc$^+$ against square roots of their corresponding scan rates, for **PgBT(F)2gTT** scan rate dependence CV data presented in Figure S43. Linear correlation was confirmed by

regression analysis (red, $R^2 = 0.99$), suggesting a diffusion limited electrochemical process according to the Randles-Sevcik equation.[2]

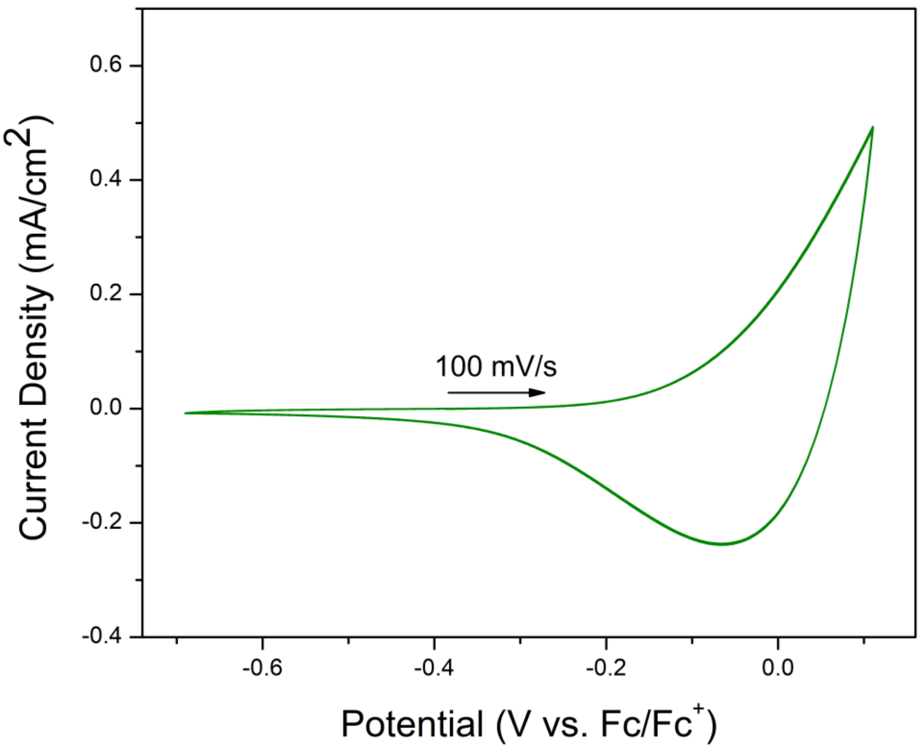

Figure S44: Cycling CV of **PgBT(F)2gTT** thin films in 0.1 M [*n*-Bu_4N]PF_6/MeCN (36 cycles). Arrow indicates direction of cyclic scanning.

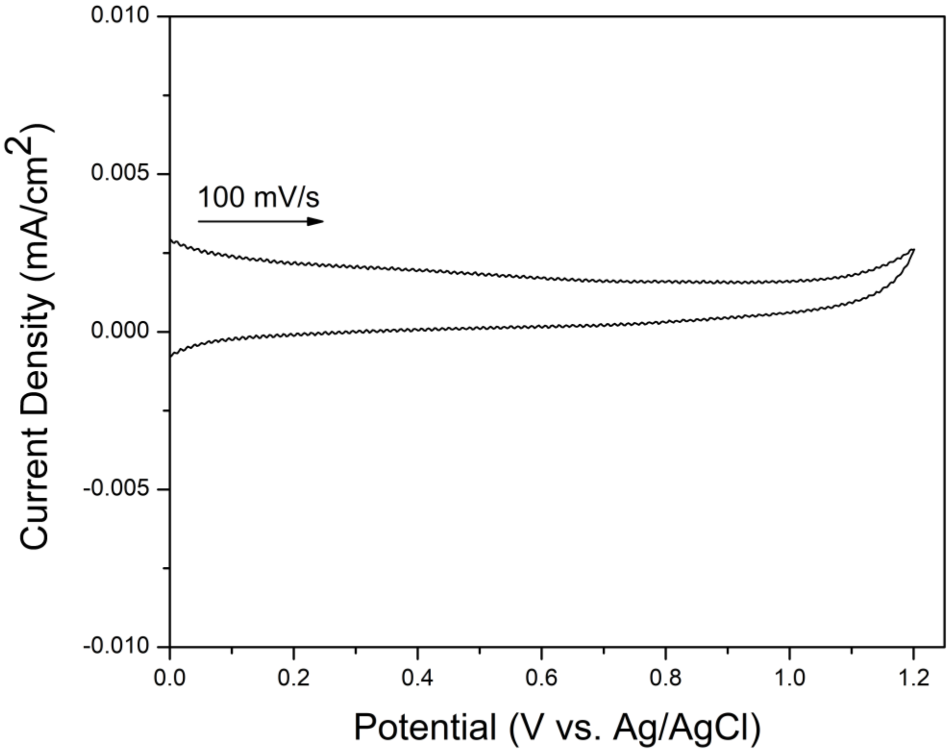

Figure S45: Background CV in 0.1 M KCl/H_2O with FTO working electrode. Arrow indicates scan direction.

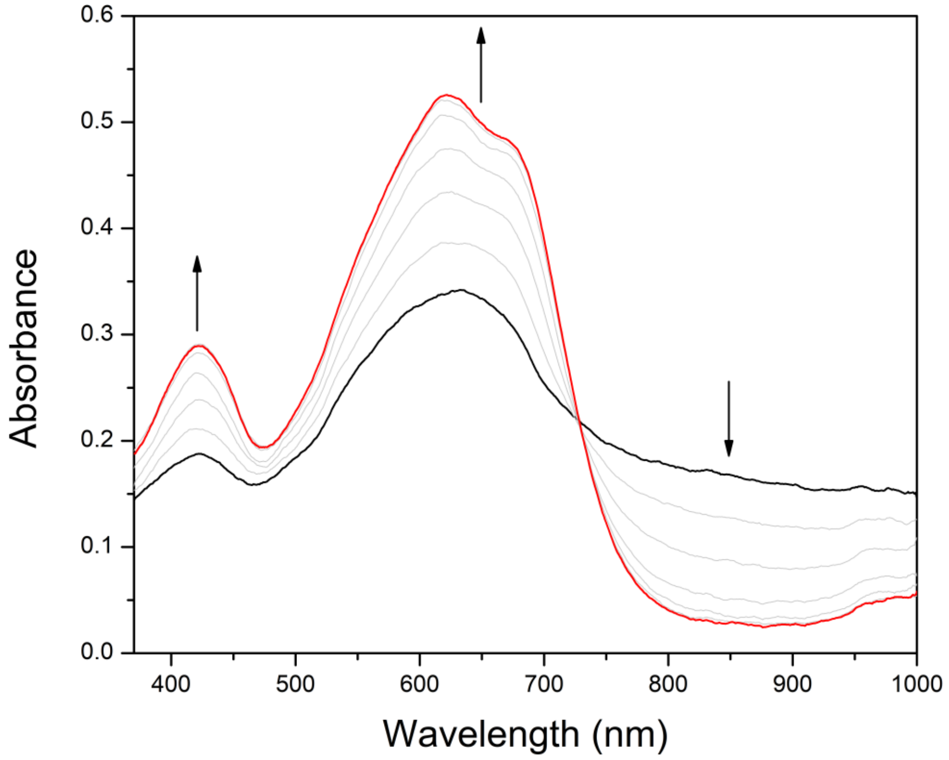

Figure S46: Solid state UV-Vis SEC of **PgBT(F)2gT** thin film in 0.1 M KCl/H_2O, at an applied potential of 0.8 V *vs.* Ag/AgCl (black), and spectral changes (grey) upon incrementally returning the applied potential to 0 V, demonstrating electrochemical reversibility. Arrows indicate spectral progression.

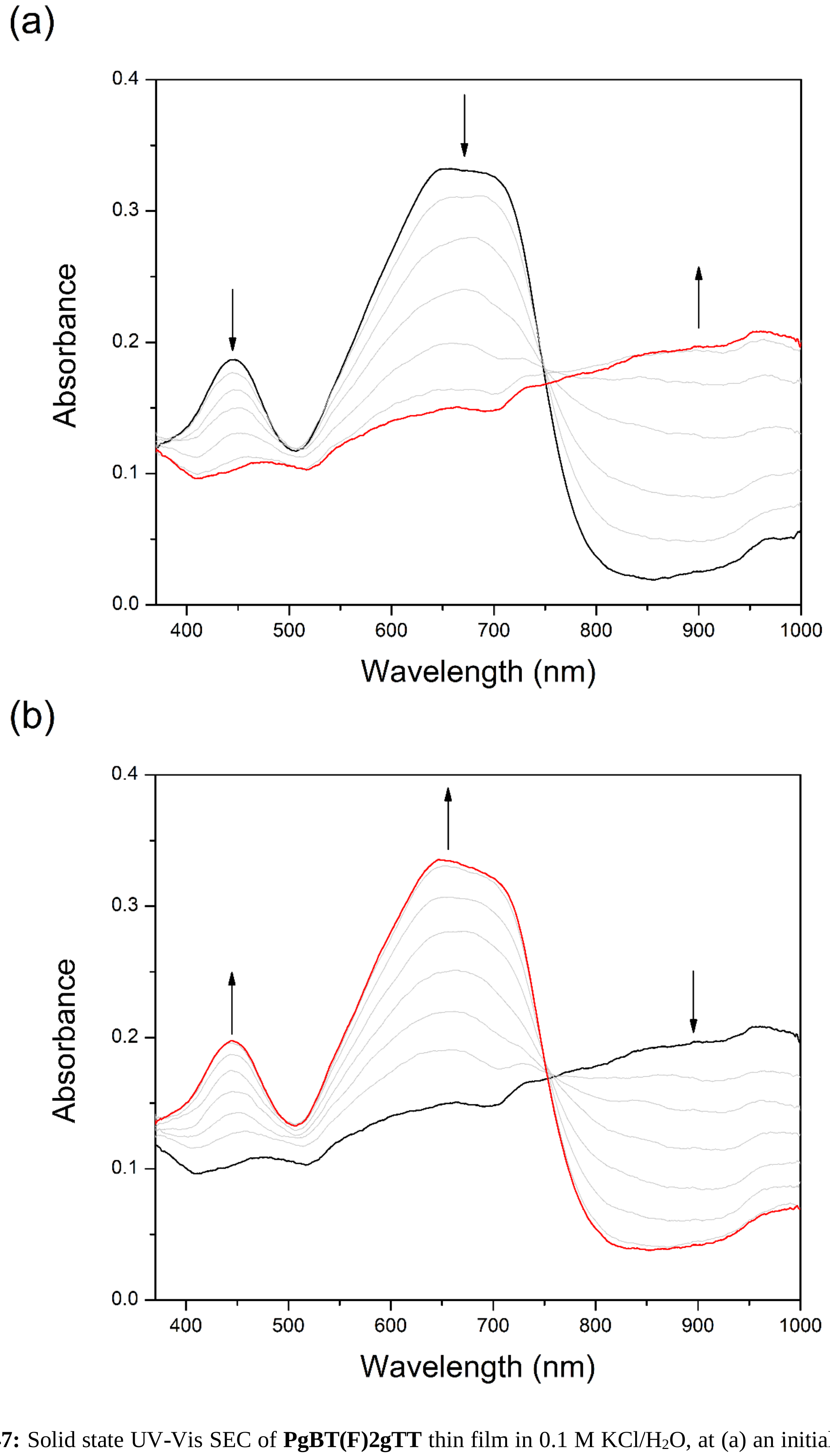

Figure S47: Solid state UV-Vis SEC of **PgBT(F)2gTT** thin film in 0.1 M KCl/H_2O, at (a) an initial applied potential of 0 V *vs.* Ag/AgCl (black), which was incrementally increased (grey) up to 0.8 V (red). Onset of spectral changes indicating polymer oxidation was observed from 0.3 V. From (b) 0.8 V (black), the applied oxidative potential was

then incrementally decreased (grey) back down to 0 V (red), to demonstrate electrochemical reversibility. Arrows indicate spectral progression.

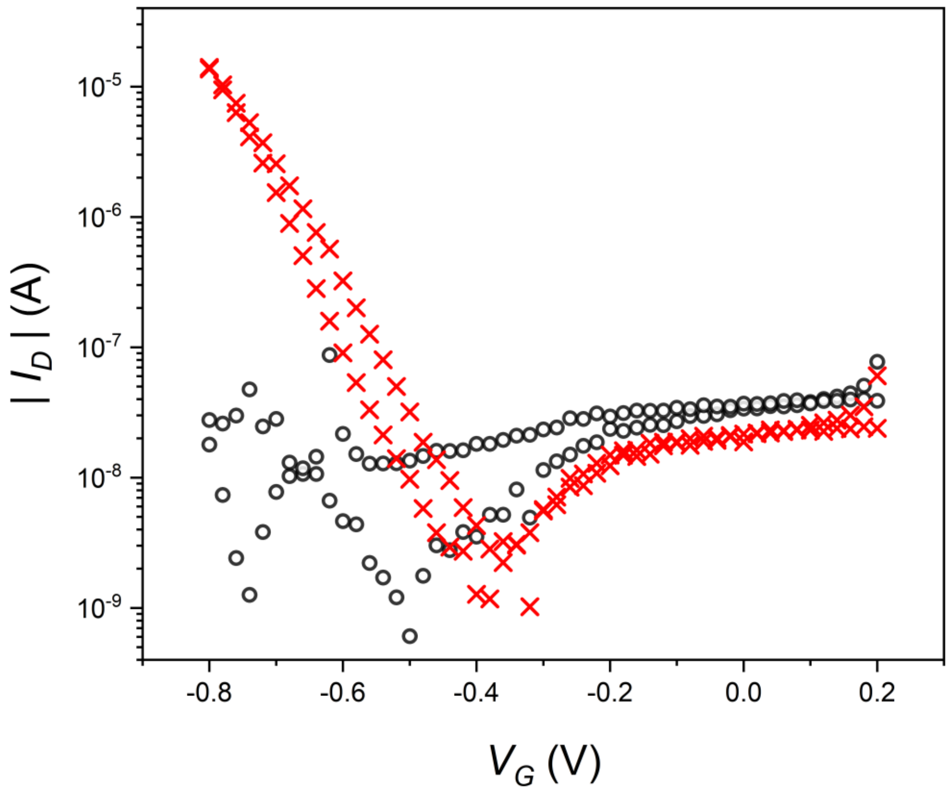

Figure S48: Log scale transfer curve of **PgBT(F)2gT** (red) with gate leakage current (black).

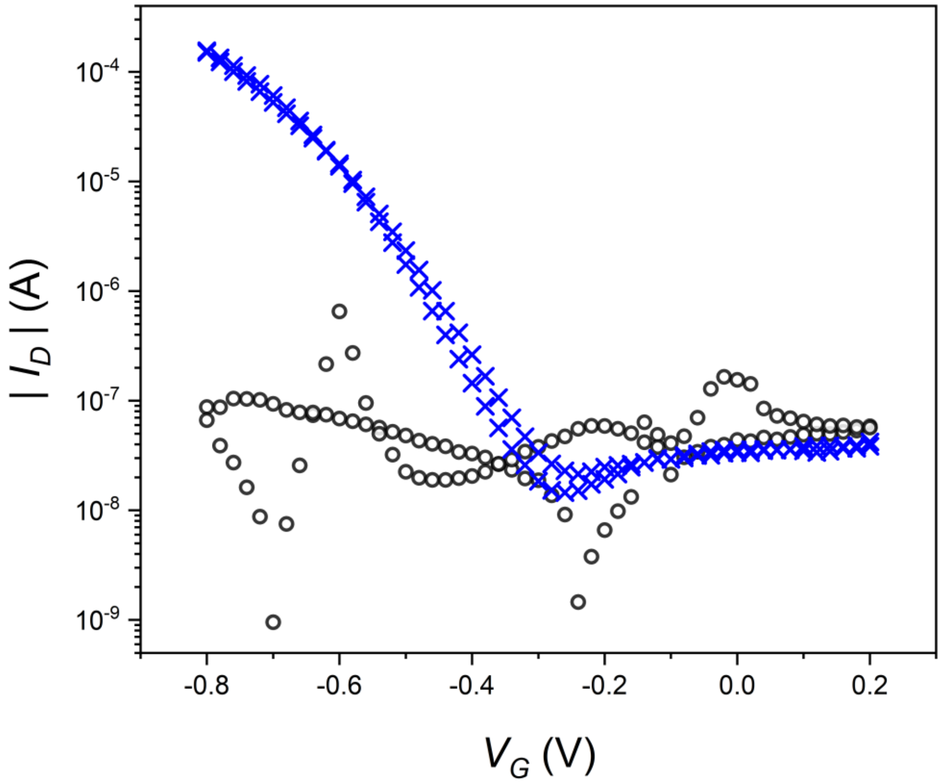

Figure S49: Log scale transfer curve of **PgBT(F)2gTT** (blue) with gate leakage current (black).

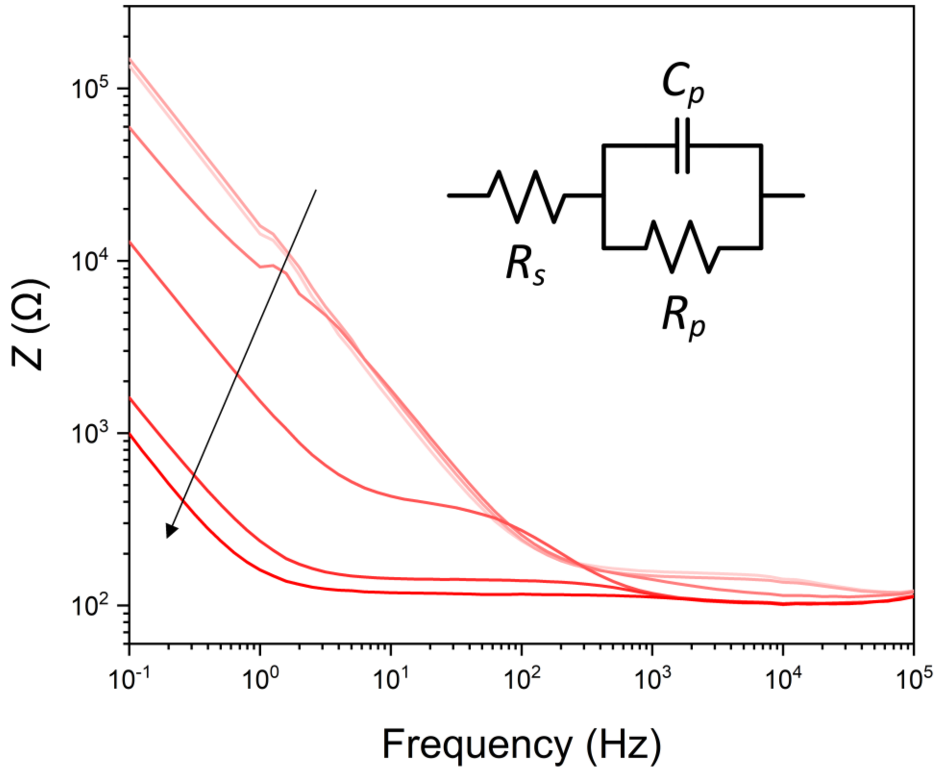

Figure S50: EIS curves for **PgBT(F)2gT** as a function of offset DC voltage, ranging from -0.2 to 0.8 V vs. Ag/AgCl. Arrow indicates data collected at higher DC offset. Inset shows equivalent circuit model applied to model EIS data.

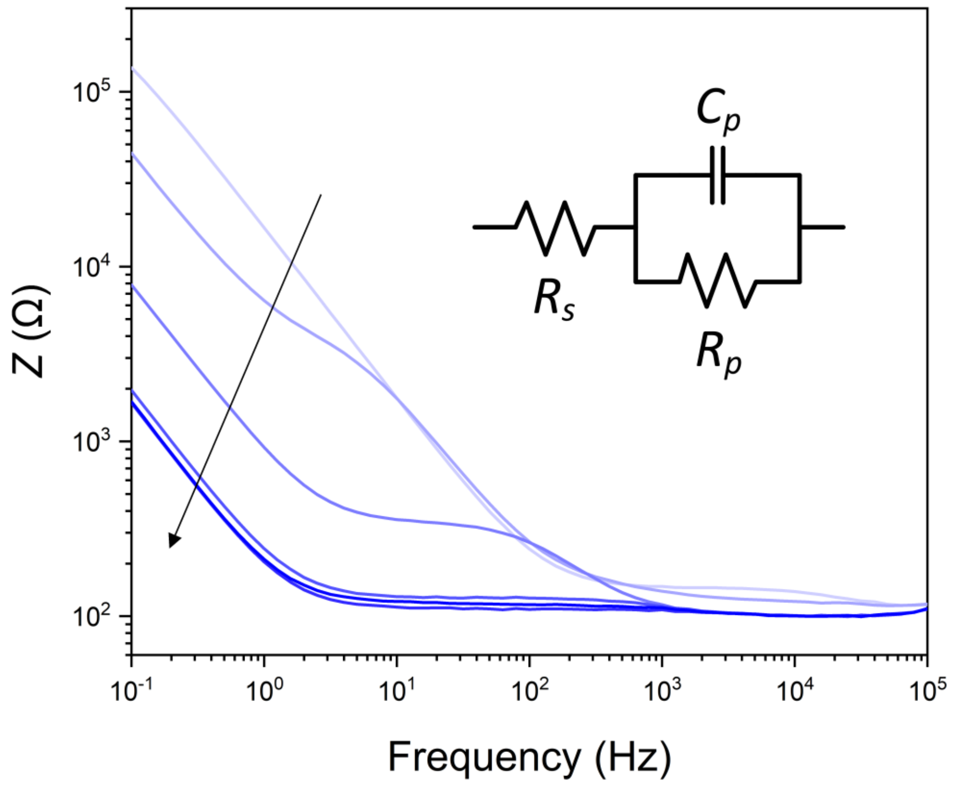

Figure S51: EIS curves for **PgBT(F)2gTT** as a function of offset DC voltage, ranging from -0.2 to 0.8 V vs. Ag/AgCl. Arrow indicates data collected at higher DC offset. Inset shows equivalent circuit model applied to model EIS data.

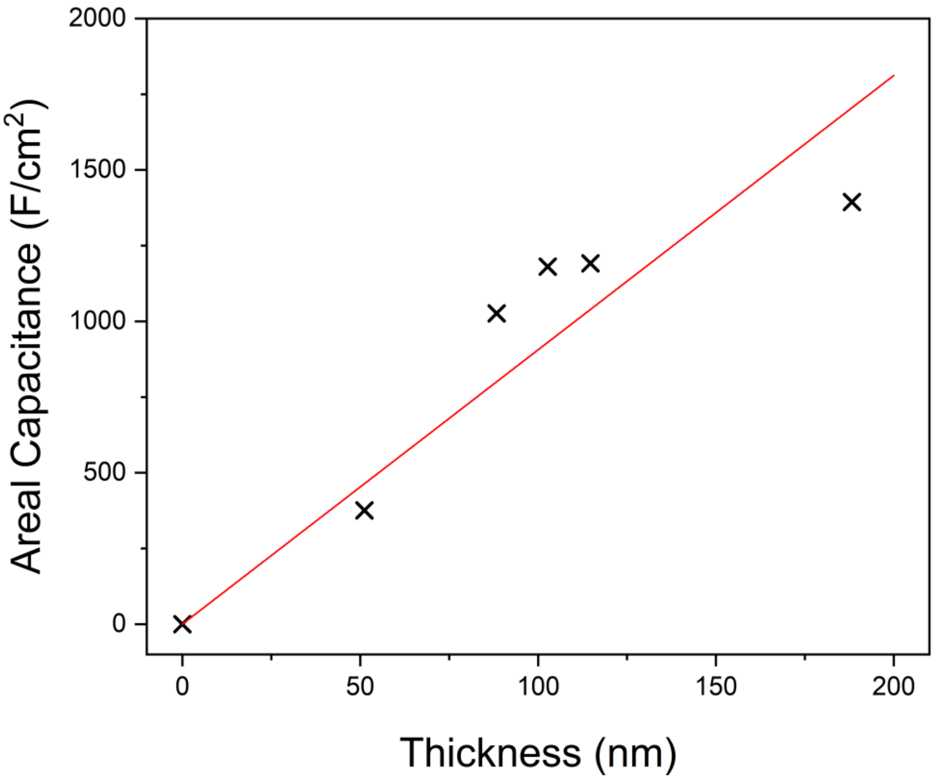

Figure S52: Areal capacitance measured by EIS against thin film thickness for **PgBT(F)2gT**. Linear regression analysis shown in red (R^2 = 0.97).

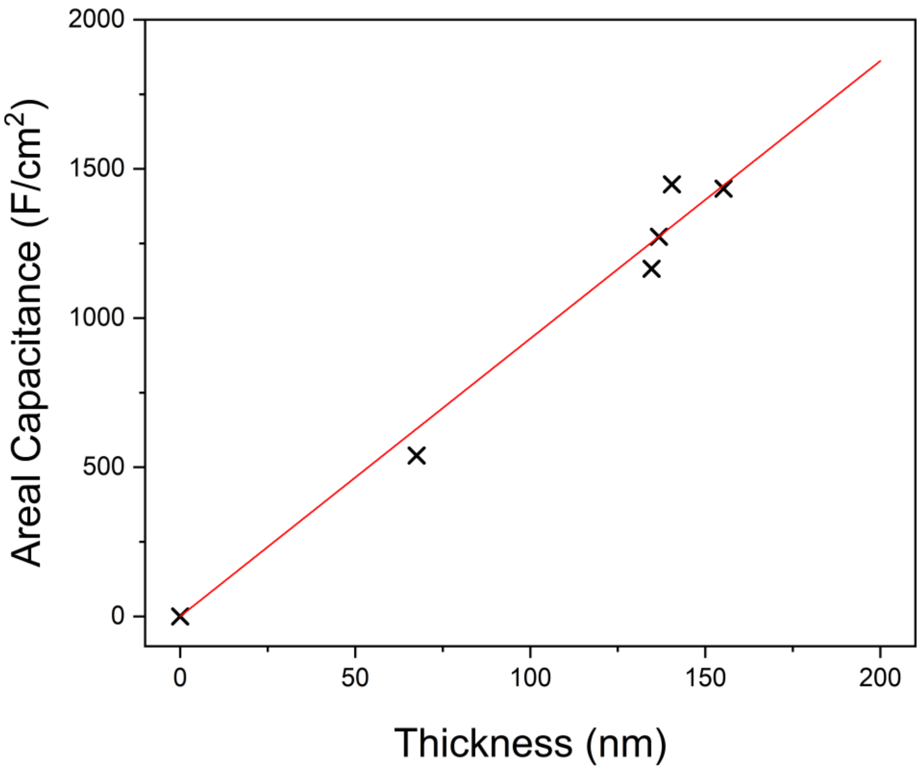

Figure S53: Areal capacitance measured by EIS against thin film thickness for **PgBT(F)2gTT**. Linear regression analysis shown in red (R^2 = 0.99).

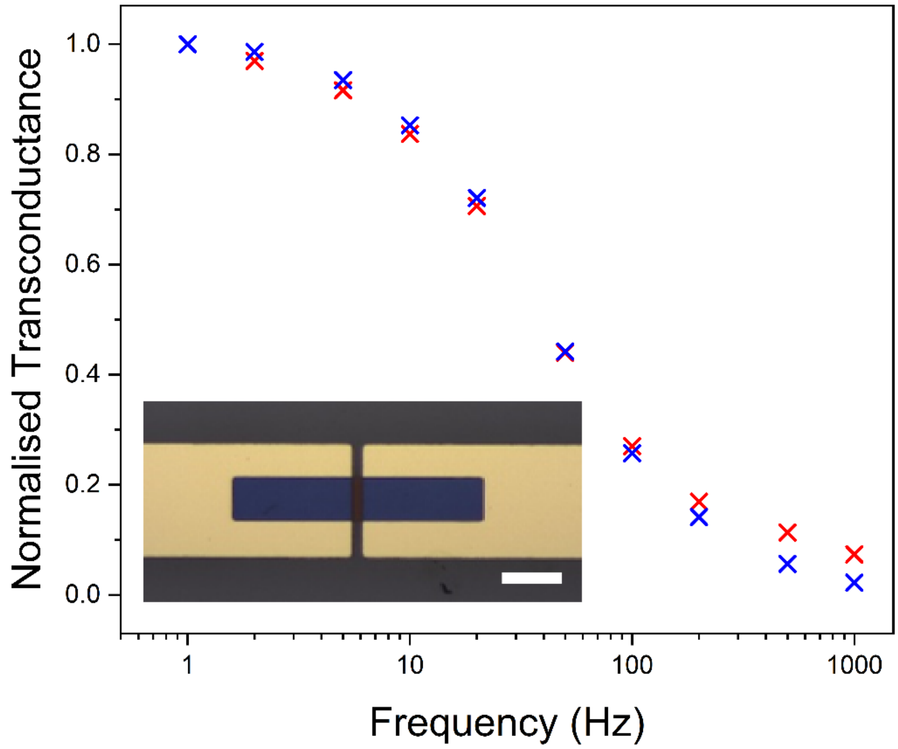

Figure S54: Normalised frequency response of **PgBT(F)2gT** (red) and **PgBT(F)2gTT** (blue). An image of the OECT device taken under microscope examination is shown in the inset (scale bar = 100 μm). Due to marginal area of active layers, cut-off frequency (~30 Hz) is underestimated by *ca.* 4.6 times.

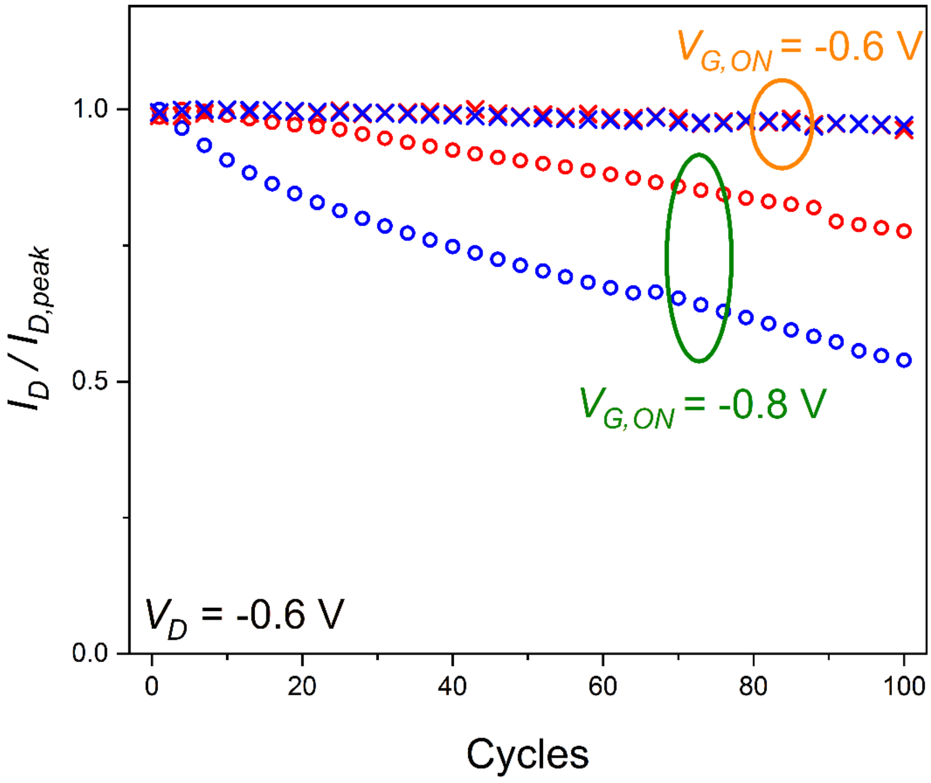

Figure S55: OECT cycling tests for **PgBT(F)2gT** (red) and **PgBT(F)2gTT** (blue) under the turn-on voltage ($V_{G,ON}$) of -0.6 V (cross) and -0.8 V (circle).

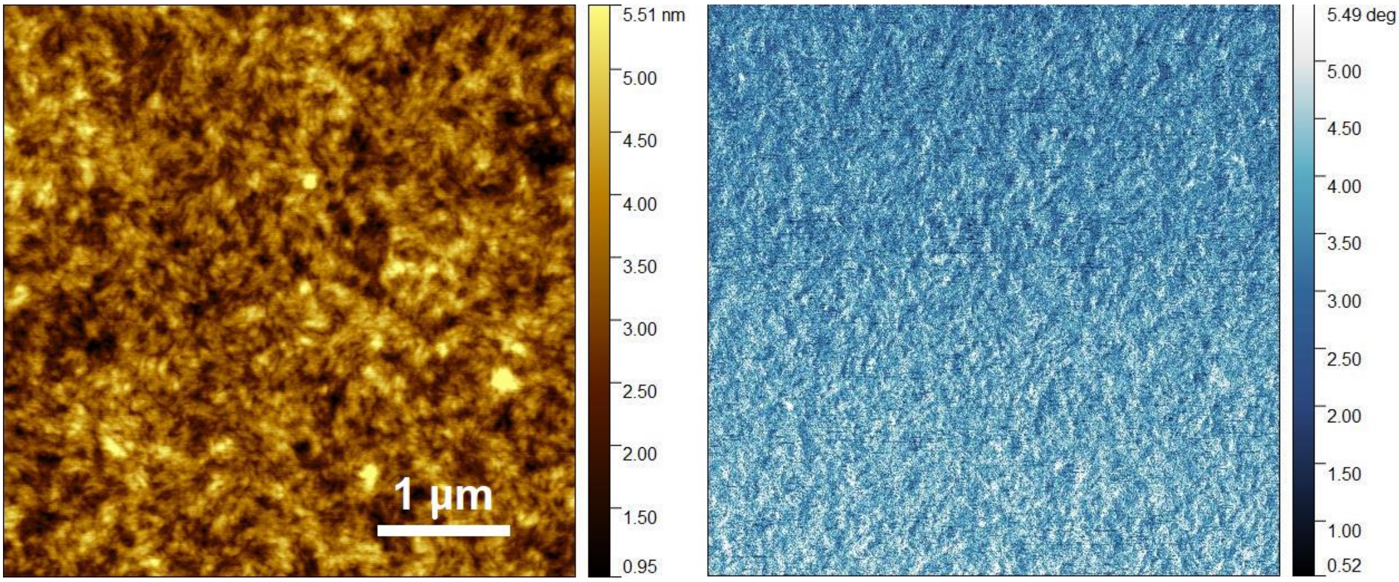

Figure S56: AFM images of **PgBT(F)2gTT**.

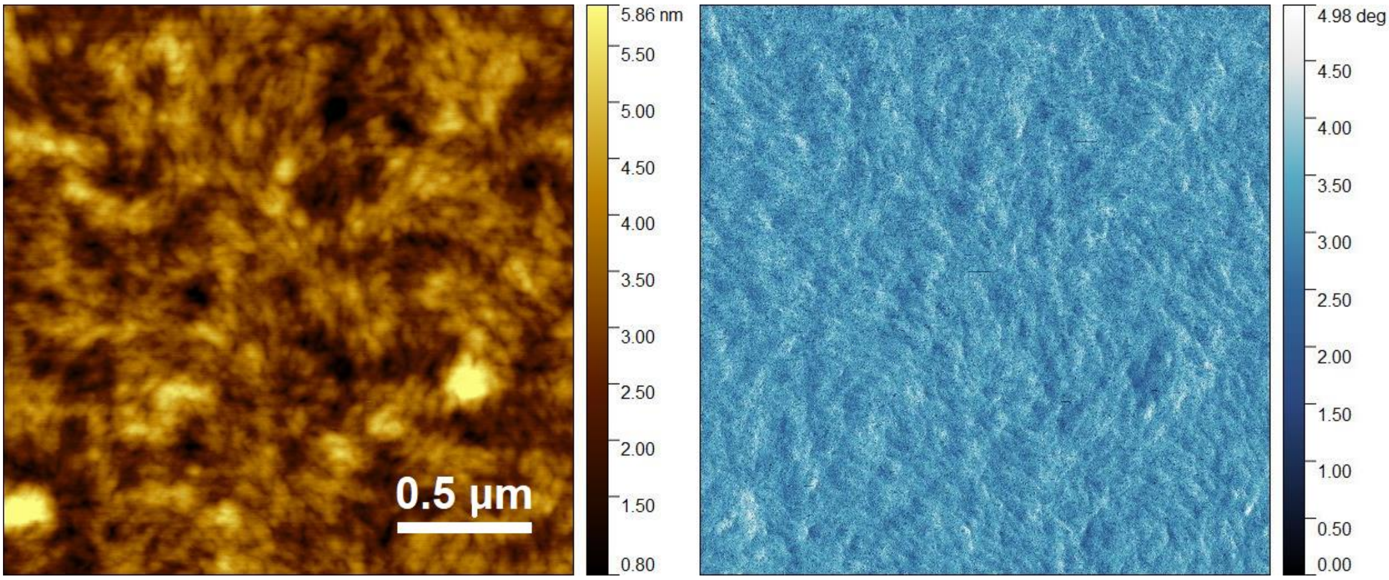

Figure S57: AFM images of **PgBT(F)2gTT** at closer magnification, showing microfibrillar structure.

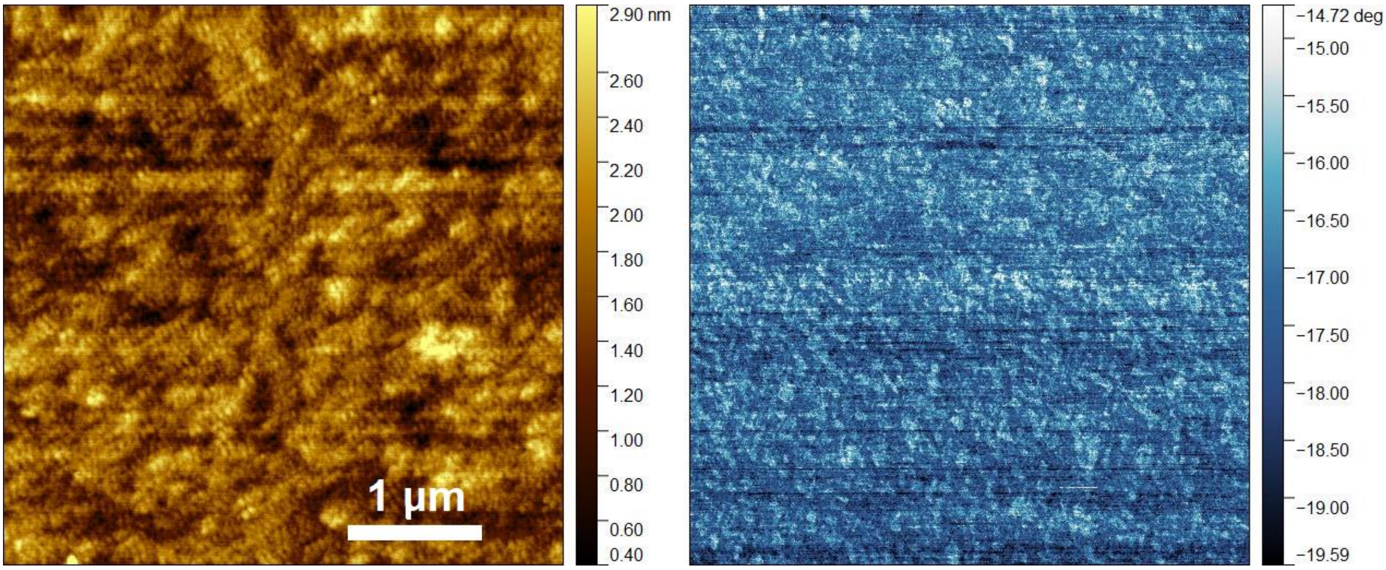

Figure S58: AFM images of **PgBT(F)2gT**.

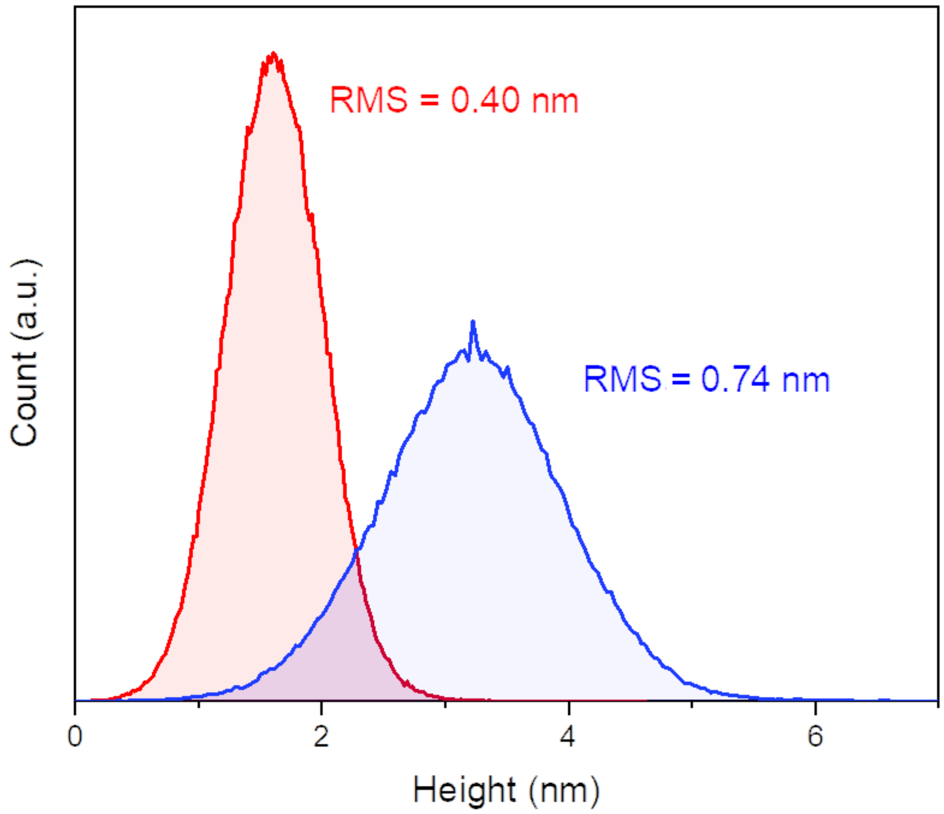

Figure S59: RMS mean surface roughness of **PgBT(F)2gT** (red) and **PgBT(F)2gTT** (blue) thin films.

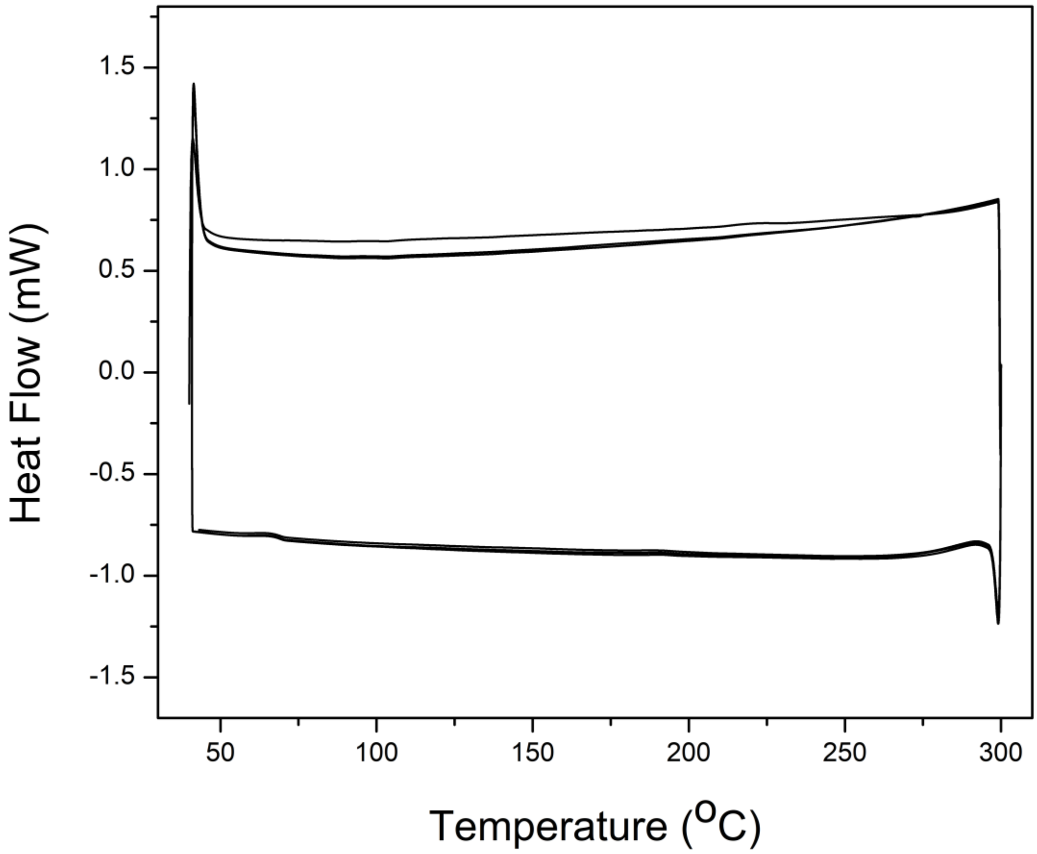

Figure S60: DSC trace (3 cycles) of **PgBT(F)2gT** between 40 – 300 °C.

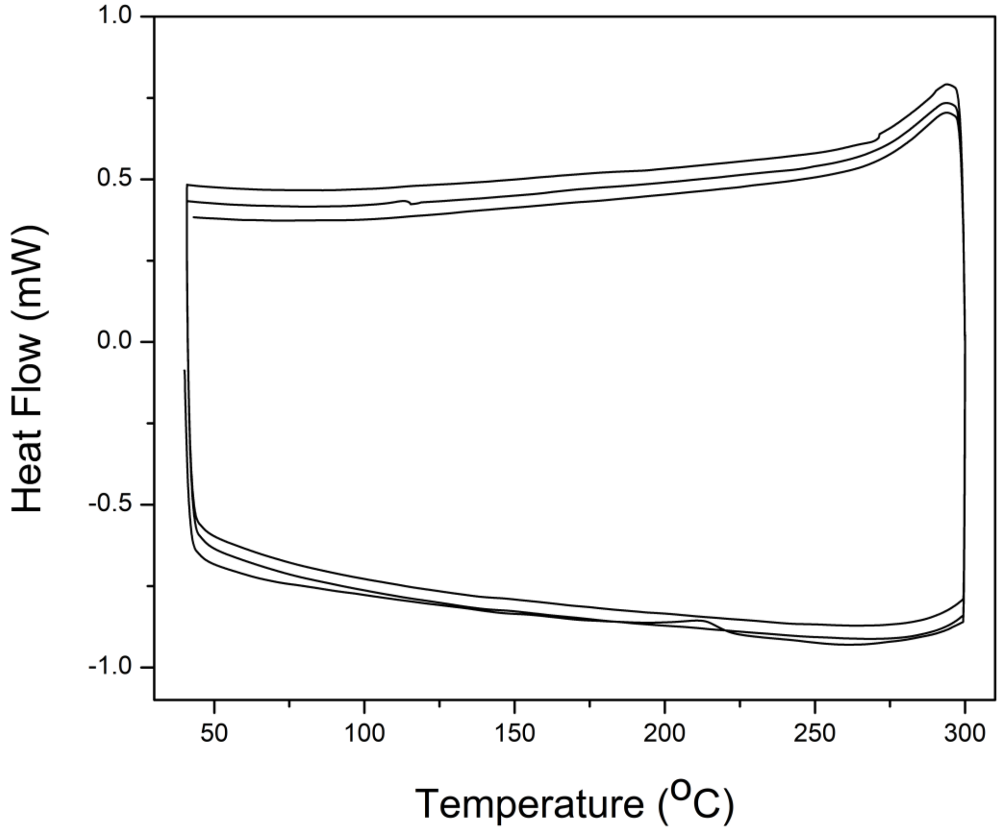

Figure S61: DSC trace (3 cycles) of **PgBT(F)2gTT** between 40 – 300 °C.

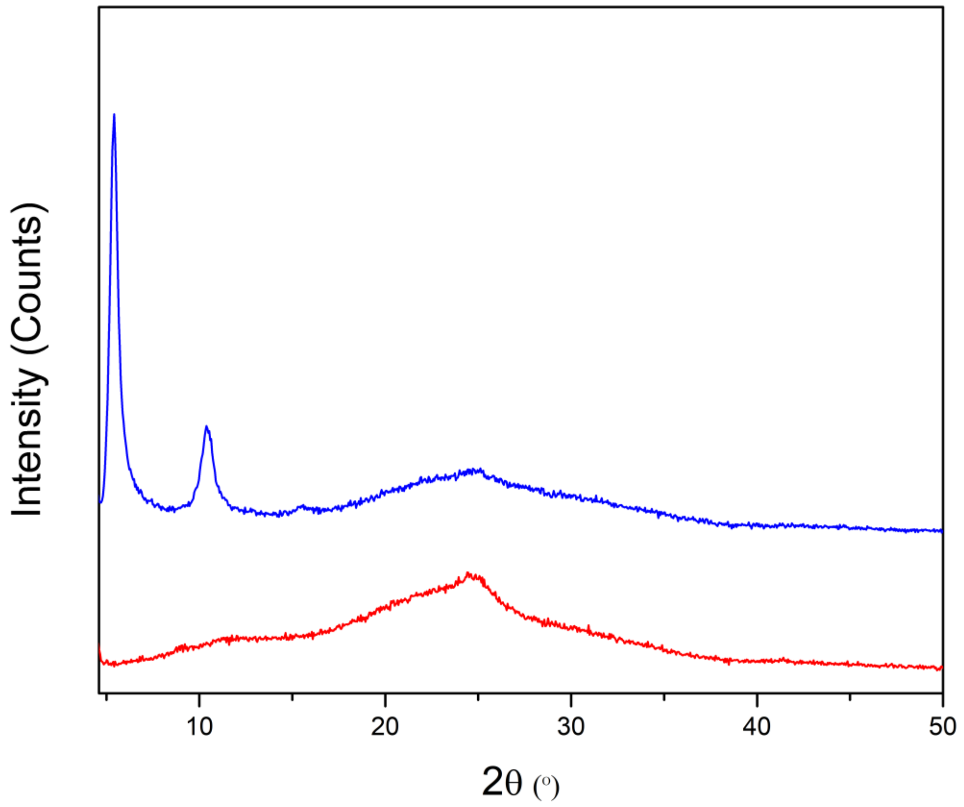

Figure S62: XRD of **PgBT(F)2gT** (red) and **PgBT(F)2gTT** (blue).

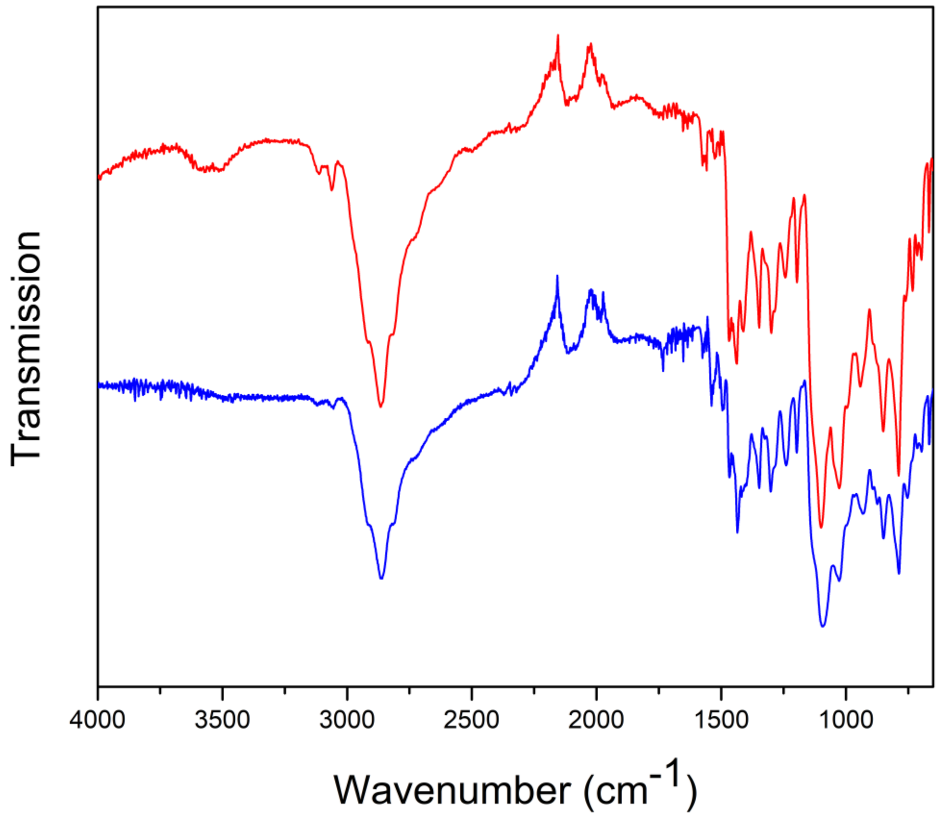

Figure S63: ATR-IR of **PgBT(F)2gT** (red) and **PgBT(F)2gTT** (blue).

REFERENCES

1. Creamer, A.; Casey, A.; Marsh, A. V.; Shahid, M.; Gao, M.; Heeney, M., *Macromolecules* **2017,** *50*, 2736.
2. Lin, S.; Usov, P. M.; Morris, A. J., *Chem. Comm.* **2018,** *54*, 6965.
